# Genetics studies indicate that neural induction and early neuronal maturation are disturbed in autism

**DOI:** 10.3389/fncel.2014.00397

**Published:** 2014-11-19

**Authors:** Emily L. Casanova, Manuel F. Casanova

**Affiliations:** Department of Psychiatry and Behavioral Sciences, School of Medicine, University of LouisvilleLouisville, KY, USA

**Keywords:** neuropathology, neurogenesis, epilepsy, schizophrenia, neocortex, synapse, dendrite

## Abstract

Postmortem neuropathological studies of autism consistently reveal distinctive types of malformations, including cortical dysplasias, heterotopias, and various neuronomorphometric abnormalities. In keeping with these observations, we review here that 88% of high-risk genes for autism influence neural induction and early maturation of the neuroblast. In addition, 80% of these same genes influence later stages of differentiation, including neurite and synapse development, suggesting that these gene products exhibit long-lasting developmental effects on cell development as well as elements of redundancy in processes of neural proliferation, growth, and maturation. We also address the putative genetic overlap of autism with conditions like epilepsy and schizophrenia, with implications to shared and divergent etiologies. This review imports the necessity of a frameshift in our understanding of the neurodevelopmental basis of autism to include all stages of neuronal maturation, ranging from neural induction to synaptogenesis.

## Introduction

Neuroblasts require precise extrinsic and intrinsic signals to acquire their unique, semi-predetermined identities. For instance, the complement of developmental signals that produce a serotonergic neuron in the raphe nucleus are different from those that produce dopaminergic neurons in the substantia nigra, leading genetically similar neuroblasts along divergent paths (Hynes and Rosenthal, 1999). Though signals for growth and differentiation may funnel through similar pathways for all neurons, such as the Wingless Integration Site (Wnt), Hedgehog, and Transforming Growth Factor-β (TGF-β) families, the effectors that regulate these pathways can vary considerably from one brain structure to the next, allowing the creation of distinct boundaries and the refinement of cellular identities (Wolpert and Kerszberg, 2003). From this basic arrangement arise not only variations in morphology but function, and together these different neuronal species commune to produce a finely-tuned, well-coordinated network of cells.

However, in autism spectrum conditions (referred to here collectively as “autism”), neuroimaging and electrophysiology suggest that these networks are prone to disruption and that disparate cognitive modules communicate comparatively asynchronously (Just et al., 2007; Sokhadze et al., 2009). In support, many autistic people, for instance, have difficulties in processing vision and speech simultaneously, two faculties which are normally well-intertwined for most people (Murray et al., 2005; Stevenson et al., 2014). In addition, cognitive tasks that require the coordination of large networks of cells, such as socialization, language, and executive functions, are usually the most impaired in the conditions; meanwhile, those tasks that necessitate smaller networks, skills sometimes associated with savantism, are more frequently spared (Treffert, 2010).

Reflective of the large-network incoordination in autism, neuropathological studies performed over the last several decades indicate that the brains of autistic individuals are characterized by heterogeneous dysgeneses or malformations, ranging from subtler dysplasias affecting lateral cell dispersion within minicolumns, to the more obvious heterotopias which can sometimes be seen on magnetic resonance imaging (MRI) as Unidentified Bright Objects (UBO) (Nowell et al., 1990; Casanova et al., 2002; Wegiel et al., 2010). At the microscopic level, altered cell morphologies (e.g., reduced soma size) are also often noted (Casanova et al., 2002).

Across a series of three separate studies since 1998, various neuropathologists have found direct evidence of neocortical dysgenesis in autism ranging from 92 to 100% of their subjects, indicating that disturbances to early neocortical development are likely fundamental to the conditions (Bailey et al., 1998; Wegiel et al., 2010; Casanova et al., 2013). Wegiel et al. also reported that subcortical, periventricular, hippocampal, and cerebellar heterotopias were present in 31% of cases, meanwhile 62% of their subjects exhibited cerebellar dysgenesis. These types of cortical, subcortical, and periventricular malformations are strongly linked with epileptic susceptibility in the general population and probably explain the high rates of epilepsy in autism (Raymond et al., 1995).

Considerable research energies have been devoted to the study of autism at the level of the neurite and synapse (Auerbach et al., 2011). It therefore becomes a challenge to rectify what appears to be conflicting evidence arising from the fields of neuropathology and genetics. A number of studies have suggested that neurite- and synapse-associated gene products, such as Contactin-associated Protein-like 2 (Cntnap2), Neuroligin-4, X-linked (Nlgn4x), and Neurexin-1-alpha (Nrxn1), may in fact be involved in earlier stages of differentiation than typically acknowledged, resulting in defects of migration and other indications of disturbance to pre-migratory cell fate determination (Peñagarikano et al., 2011; Shi et al., 2013; Zeng et al., 2013). We have therefore considered the possibility that some genes expressed during early stages of neuronal development may continue to have rippling effects on later stages of differentiation, permanently perturbing neuronal maturation when their gene products are impaired. Other genes may also express considerable functional redundancy at numerous stages of cell growth and development, likewise leading to shared disturbances in neurogenesis and neurite extension or synaptogenesis. Thus, we have gathered a core set of idiopathic and syndromic high-risk autism genes in order to perform an in-depth review of their myriad cellular functions in early brain development and determine whether commonalities exist that may explain the broad range of findings in autism.

## Functional overlap of core autism genes

**Figure 1:**
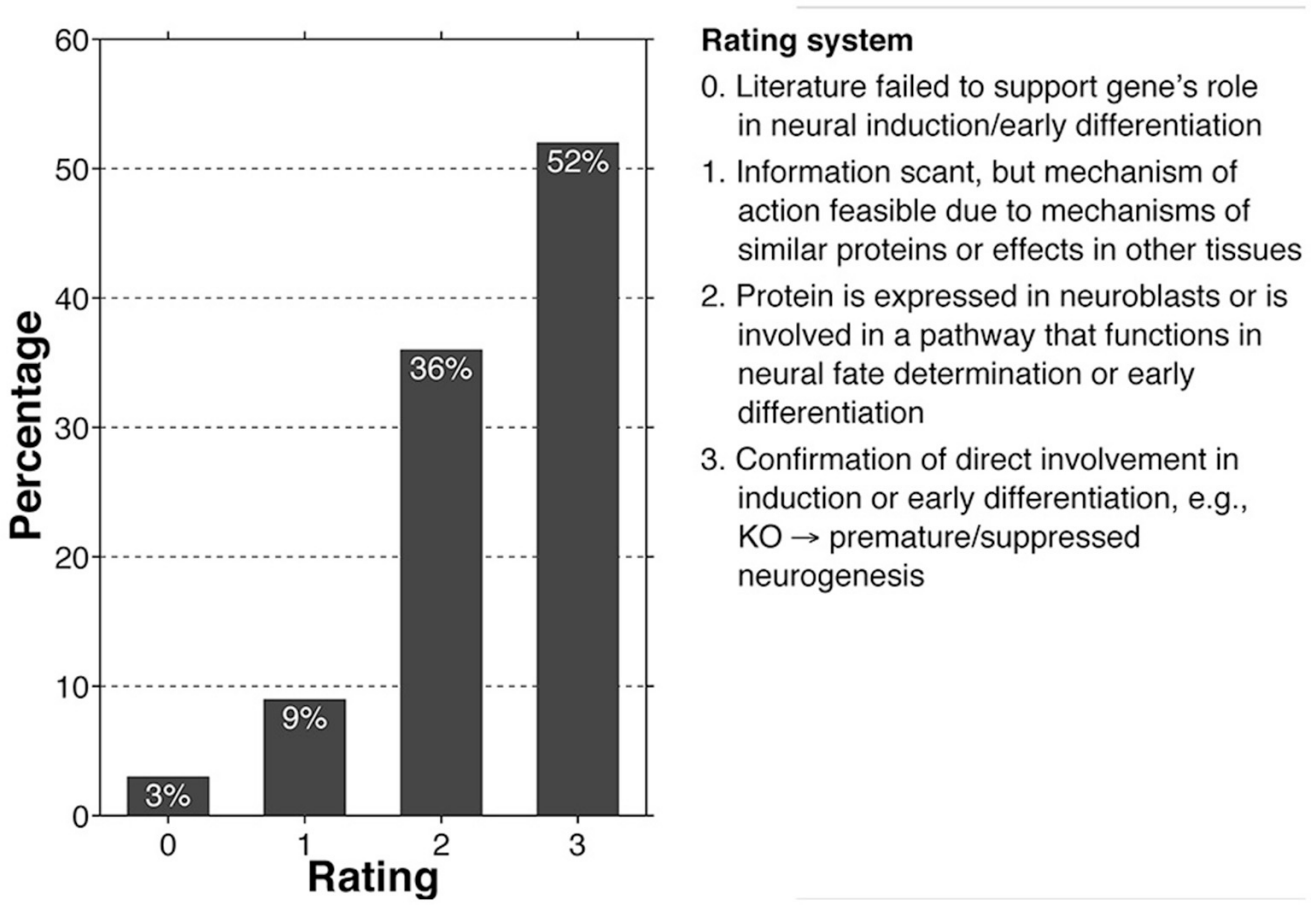
**Bar graph showing the number of high-risk autism core genes that fall within a given rating (0–3), with criteria for the rating system**.

We have performed an in depth review of the literature surrounding a core set of 197 high-risk autism-related genes. Our list was compiled from the *SFARI* and *AutismKB* databases. We selected the “Syndromic,” “High Confidence,” and “Strong Candidate” categories from the *SFARI* database and the core and syndromic datasets (version 2) from the *AutismKB* database in order to derive our final core set (Banerjee-Basu and Packer, 2010; Xu et al., 2012; databases accessed on 6/15/14). The literature was then reviewed in depth, focusing on regulatory roles for each gene in neurogenesis, neural induction, and neuroblast differentiation. In order to better summarize our findings, we applied a semi-quantitative rating system to each gene, ranging from “0” which indicates that there is no known relation between the gene product function and neuroblast development, to “3” in which there is a confirmed direct relationship, the latter most frequently manifest as either premature or delayed neurogenesis (see Figure 1). We also addressed each gene's involvement in either neuritogenesis or synaptogenesis. If abnormal branching, synaptomorphology, or synaptophysiology were reported in human cases, animal models, or *in vitro* studies for a specific gene, this was considered a “hit.”

In addition to reviewing the various functions of each gene product, we also studied their involvements in epilepsy and schizophrenia. As discussed earlier, seizure disorder occurs frequently in autism, and within the general epileptic population dyplasias, heterotopias, and ectopias are often seats of epileptiform activity, suggesting links in the etiologies of autism and epilepsy (Avoli et al., 1999). In order to determine a relationship between epilepsy and a given gene, the literature was searched for indications of the gene's involvement in epileptic susceptibility; modest overlap was considered a “hit.” Likewise, gene overlap between the core set and schizophrenia risk genes was assessed using the *SzGene* database and considered a “hit” if contained therein (Allen et al., 2008).

According to our review, 88% of core genes fell within the 2–3 rating categories, while only 12% fell within the 0–1 rating, suggesting that the vast majority of high-risk autism genes help to regulate neural induction and early neuroblast differentiation (Figure 1; see Supplementary Table 1 for full gene list and justifications for individual ratings). In addition, as was predicted at the outset of our review, 80% of the core set genes influence processes of neuritogenesis, synaptogenesis, and plasticity, primarily indicated by loss-of-function *in vitro* and *in vivo* studies. Most of these neuritic and synaptic genes (86%) likewise overlap with the 2–3 rating category, suggesting that the majority of core genes influence numerous stages of neuronal development and are not limited to singular processes, many of which have probably been exapted for a variety of related uses (Supplementary Table 1).

**Figure 2:**
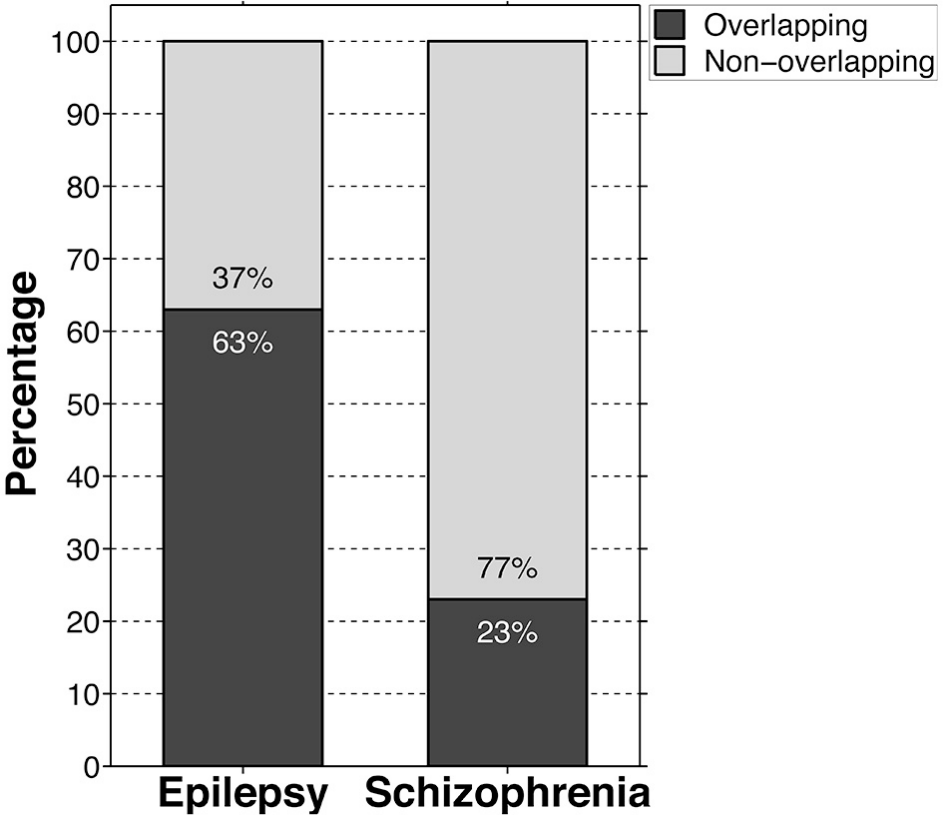
**Bar graph showing the percentage of high-risk autism core genes that are at least modestly cross-indicated in epileptic and schizophrenic etiologies**. Black bars indicate genetic overlap, meanwhile gray bars indicate no evidence of overlap.

Meanwhile, we found that approximately 63% of the core genes overlapped with the molecular etiology of epilepsy, spanning all areas of function (Figure 2; Supplementary Table 2). Within the general autistic population, epilepsy occurs alongside autism in approximately 26% of cases, meanwhile epileptiform activity can be seen in about 60% of cases (Spence and Schneider, 2009; Viscidi et al., 2013). Viscidi et al. also reported, however, that risk of epilepsy increases with severity of autistic symptomology, and therefore considering the high genetic overlap between autism and epilepsy presented here, this suggests that forms of autism with strong genetic backgrounds could prove more severe symptomologically, both in terms of autism and seizures.

In contrast, only approximately 23% of the autism core genes overlapped with schizophrenia risk genes, neurotransmitter and ion transport functions being overrepresented in this group, comprising 42% of the total overlap (Figure 2; Supplementary Table 2). Nevertheless, trophic factors, tumor suppressors, transcriptional regulators, and regulators of signal transduction likewise comprised a sizable minority accounting for 26% of the overlap. The remaining genes largely include products involved in cell-cell adhesion and cytoskeletal regulation (12%), signal transduction and oxidation processes within the mitochondria (4%), and housekeeping pathways (e.g., purine and lipid synthesis) (9%). Though it is difficult to draw inferences about schizophrenia and autism given the paucity of genetic commonality and the range of potential gene validity contained within the *SzGene* database, the heavy preponderance of neurotransmitter-related mutations in the overlap implies that there may be some fundamental differences in the two conditions' etiologies. Of relevance to this topic, though autism is highly comorbid with epilepsy, only approximately 2% of epileptics in a large Danish study were also admitted to hospital and diagnosed with either schizophrenia or a schizophrenia-like psychosis, indicating little in the way of developmental overlap despite that 4/5ths of the overlapping schizophrenia-risk genes are likewise implicated in epilepsy (Qin et al., 2005). Interestingly, as evidenced by the early literature on schizophrenia, scientists originally believed that epilepsy was a protective factor against the development of schizophrenic psychosis (de Meduna, 1936). However, that work was eventually overturned and it is now believed that epilepsy occurs no more frequently in schizophrenia than in the general population, with the exception of schizophrenia-like psychotic features occasionally associated with temporal lobe epilepsy (Qin et al., 2005). This type of psychosis occurs in epilepsy in only approximately 4% of the epileptic population and is strongly associated with idiopathic, rather than organic, forms of the condition (Schmitz and Wolf, 1995). It should be noted once again that, given the scarcity of genetic overlap, caution is warranted when interpreting these observations. However, we hope that further combined neuropathological studies on autism, epilepsy, and schizophrenia may help delineate whether there exists a divergence in their developmental trajectories and how that translates at the cellular and tissue levels.

In summary, although 80% of core autism genes appear to influence post-migratory stages of neuronal differentiation, such as neuritogenesis and synapse development or remodeling, at least 88% of the core set are integral in earlier stages of neural specification, tying together evidence of gross structural neuropathology in autism with reports of neuritic and synaptic dysfunction.

## Convergence due to modularity

### Modularity

DNA houses templates for a vast number of gene products. Surprisingly, though specific regulatory elements vary considerably according to cell type, all cells appear to reuse similar pathways throughout processes of cell growth, division, and differentiation. Examples of some of these pathways mentioned earlier include the Wnt, Hedgehog, and TGF-β families, each of which has been well-studied during embryonic development of numerous tissue types (Wolpert and Kerszberg, 2003). As such, these super-pathways can be considered *modules* which, through variations in their regulatory partners and upstream effectors, lead to the differentiation of one sister cell from another (Schlosser and Wagner, 2004). Rather than recreating the wheel from scratch, evolution has elected to conserve these super-pathways and instead derive new cell-specific regulators of these and similar modules that allow the ever-expanding complexity of different tissue types. The brain itself is an extraordinarily complex array of unique cells, varying extensively in morphology and physiology; in order to create such tissue complexity, many brain-specific regulators must exist to define the numerous boundaries that occur within the adult brain, both in terms of regional diversity and microcircuit specificity.

A *module* is defined as “one of a set of separate parts that can be joined together to form a larger object.” The basic cellular functions of the core gene set reviewed here can also be considered modular and typically funnel through the super-pathways previously mentioned. Though their utilities vary, the functions of these gene products overlap in several key ways, some of which have been individually covered in the related literature (Table 1A; Supplementary Table 3). Proper functioning of all of these processes together orchestrates neural induction and neuronal differentiation: when any one of them is disturbed, fate determination can be altered, as shown in the succeeding examples from the core list of genes. If pervasive, this may lead to disruption within the larger network of cells and to conditions such as autism.

**Table 1:** **Tables summarizing the basic molecular/biological functions of gene products targeted by mutations in the core autism set of genes (A) and larger functional domains into which these can be funneled, leading to disruptions in neuronal induction and differentiation whenever their effectors are impaired (B)**.

**(A) Basic cellular functions targeted in autism**	**(B) Induction and differentiation are:**
Calcium regulation Neurotransmitter/Neuromodulation regulation Vesicular transport Cation channel synthesis and ion transport Purine/Pyrimidine metabolism Regulation of cytoskeleton, cell adhesion, and ciliation GTPase/ATPase activity Transcriptional/Translational regulation Chromatin remodeling Methylation and acetylation Neurotrophic factor activity Intracellular signaling transduction Ubiquitination Mitochondrial regulation/Cell detoxification Immune regulation	Activity-dependent Structure-dependent Product-dependent Stress-sensitive

### Neural induction and differentiation are activity-dependent

Excitation is a requisite for the implementation of all stages of neural development (Spitzer, 2006). Of particular importance to the cortical malformations discussed here, excitation drives progenitor expansion and neurogenesis, as well as later stages of differentiation and plasticity (Kempermann et al., 2000; Ge et al., 2007). This is known as *activity-dependent development*. The categories of calcium regulation, neurotransmitter and neuromodulator regulation, cation channels and transport, and even purine metabolism all converge to produce the necessary excitatory drive that induces neurogenesis and neuroblast maturation. Calcium wave disruption, γ-Aminobutyric Acid (GABA) signaling reduction, A-type potassium channel suppression, and disturbances in purine metabolism in neural progenitors each leads to deviation in the timing and success of neurogenesis (Weissman et al., 2004; Cancedda et al., 2007; Lin et al., 2007; Schaarschmidt et al., 2009). These examples, reflective of some of the cellular dysfunctions seen in autism, converge to produce neurons whose maturations are breached either by precocious induction and differentiation or the suppression thereof. As such, these neurons can display a gambit of features ranging from the malformation and/or misplacement of cells which may or may not retain neural stem cell (NSC) markers such as nestin or vimentin, to morphologically and migratorally normal populations that may nevertheless ectopically express characteristics atypical of their specific identities, promoting aberrant physiologies and larger network disturbances.

Another important example of the ectopic expression of a progenitor-like marker relevant to autism, epilepsy, and schizophrenia is the chloride importer, Na-K-Cl Cotransporter-1 (NKCC1) (Palma et al., 2006). Upregulated NKCC1 mRNA expression and increased intracellular chloride concentrations are typical of neural progenitors, neuroblasts, and Cajal-Retzius cells, however they are downregulated in mature pyramidal neurons, leading to the hyperpolarizing inhibitory nature of GABAergic signaling in the mature central nervous system (Pozas et al., 2008; Young et al., 2012). In resected tissues of drug-resistant temporal lobe epilepsy, for instance, mRNA levels of NKCC1 are unusually upregulated while levels of the exporter, Potassium-chloride Transporter Member 5 (KCC2), are downregulated, suggesting that subpopulations of neurons have either failed to mature properly or have regressed (Huberfeld et al., 2007). This effect ultimately leads to GABA-induced depolarization and seizures. The drug, bumetanide, which specifically antagonizes the NKCC1 chloride importer, has not only found favor in the treatment of some epilepsies, but has also been met with success in the treatment of symptoms of autism in early drug trials and is leading to exciting new developmental theories regarding the molecular pathology of autism (Dzhala et al., 2005; Ben-Ari et al., 2012; Lemonnier et al., 2012). It has also been found that downregulation of KCC2 and upregulation of NKCC1 are common reactions to neuronal stress or injury, suggesting a means of action in symptom onset in regressive forms of autism (Nabekura et al., 2002; Kim et al., 2011).

It is clear that an exaggerated excitatory propensity in pyramidal cells appears to be a risk factor for autism and epilepsy. One of the clearest examples lies in the condition known as Timothy Syndrome (TS), which exhibits at least 60% comorbidity rate with autism, the highest penetrance for syndromic autism to date (Splawski et al., 2004). The primary cause of TS is due to mutations within exon 8 of the L-type voltage-gated calcium channel gene, *CACNA1C*, which results in intracellular calcium overload in affected cells, including central nervous system (OMIM, 2011). Splawski et al. (2004) found that this exaggerated influx was due to the loss of channel inactivation and suggested, because of the high comorbidity rate, that calcium signaling was therefore strongly implicated in autism etiology. In support of this, a form of X-linked intellectual disability (XLMR) associated with congenital stationary night blindness, caused by gain-of-function mutations in another L-type voltage-gated calcium channel gene, *CACNA1F*, also exhibits comorbidity with autism. Unfortunately, this disorder is extremely rare, with most of the evidence stemming from study of a single multiplex family in which three of the five individuals affected had comorbid autism (Hemara-Wahanui et al., 2005). However, mutations in *CACNA1F* have also occurred in idiopathic autism, supporting its role as a risk gene in the condition (AutismKB, 2012).

Defects in neuronal migration are a common feature reported in idiopathic autism (Wegiel et al., 2010). Interestingly, both the chloride exporter, KCC2, and calcium signaling are vital in normal migration and may be implicated when it goes awry. Bortone and Polleux (2009) reported that expression of KCC2 was necessary for the halt in migration of interneuronal neuroblasts, achieved through the reduction in membrane potential via decreases in calcium transients. In this instance, it appears as though the chloride exporter, through the suppression of excitation, behaves as a “motogenic stop signal.” Thus, one might fathom how changes to the level of excitatory signaling implicated in autism could easily lead to the cerebral ectopias and heterotopias seen in the condition.

Calcium and glutamate activities are also tightly linked within the central nervous system, therefore it may come as little surprise that impairment in these pathways can result in phenotypic overlap. As example, loss-of-function mutations in the gene, *Glutamate Receptor, Ionotropic, AMPA 3* (*GRIA3*), lead to an XLMR, with frequent comorbid seizure disorder and autistic features (OMIM, 2014). Though sparse murine knockout studies fail to implicate Gria3 in seizure susceptibility in mouse, in human its involvement is clear given seizure comorbidity (Beyer et al., 2008). Further study is still necessary, however, to understand the physiological dysfunction resultant from *GRIA3* mutations that lead to this form of seizure- and autism-susceptible XLMR.

The process of neural excitation is a modular one, composed of numerous subunits, each of which is a potential target for disruption. As such, different genetic mutations may converge to produce an overlapping phenotype. In this instance, calcium and glutamate signaling each play important complementary roles in development and when disturbed appear to influence autism susceptibility, as well as susceptibility to seizure disorder and intellectual disability.

### Neural induction and differentiation are structurally-dependent

Many of the gene products not directly involved in cellular excitation are instead upstream, parallel to, or downstream from it. Downstream of excitatory signaling lie cytoskeletal and cell-cell adhesion complexes, whose activities grant not only the capacity for migration, but also orchestrate nuclear movements during induction and the asymmetric localization of membranous factors that stipulate that induction and further differentiation (Fields and Itoh, 1996; Hu et al., 2008). The cellular cytoskeleton is comprised of networks of microfilaments (e.g., actin), microtubules, and intermediate filaments, each of which are highly GTPase-dependent (Mammoto and Ingber, 2009). Actin provides for cellular locomotion and extension. Microtubules likewise can play roles in certain types of cilial extensions, but also provide the overall shape for the cell, supply a network of molecular tracks with which to shuttle vesicles to specific locations, and form the centrosome, anchoring the cytoskeleton to the nucleus and helping to direct nuclear movements vital for aspects of cell polarity. Meanwhile, intermediate filaments provide tensile strength for the cell (Steinert and Roop, 1988; Rodriguez et al., 2003). Each of these cytoskeletal networks makes contacts with various adhesion complexes and cell-cell junctions, relationships which are often calcium-sensitive, if not calcium-dependent (Hirano et al., 1987, for example). The larger relationship between coordination of the cytoskeleton and neurogenesis can be inferred in a number of studies. Chilov et al. (2011), for instance, showed that when the formation of the centrosome is prevented in radial glial cells, precocious neurogenesis occurs, resulting in early depletion of the progenitor pool and reduced neuron production. Likewise, Rašin et al. (2007) reported that maintenance of the apical endfoot of radial glia at the ventricular surface, maintained by cadherins in a Numb- and Numb-like-dependent fashion, is necessary for the retention of the progenitor pool, whose disturbance can lead to either an early loss or prolonged maintenance of the same.

But cytoskeletal dynamics play an even more fundamental role in cell proliferation and fate switching. Chen et al. (1997) discovered in a series of ground-breaking experiments that solely by altering the basic shape of endothelial cells through varied patterning of an extracellular matrix-coated substrate, cell fate could be altered. By designing a substrate that decreased in size thereby restricting cellular extension (i.e., “cell spreading”), cells switched from growth to apoptosis, regardless of the type of matrix protein used or the antibody to integrin that was mediating adhesion. Cell fate can also be altered by adjusting the mechanical compliance of the substrate itself, ultimately affecting cell-generated traction forces which in turn communicate with the internal millieu of the cell, fine-tuning pathways involved in fate determination (Mammoto and Ingber, 2009). In summary, it appears that partial suppression of cell spreading switches many epithelial cell types from a state of proliferation to differentiation; meanwhile, complete prevention of cell spreading often leads to apoptosis (Mammoto and Ingber, 2009).

Consider then how impairment in a single cytoskeletal-related protein or RNA could irreparably alter neuronal fate in autism. In our core set of genes, there are many whose functions are directly linked with cytoskeletal dynamics. For instance, Aarskog-Scott Syndrome is an XLMR that is estimated to occur in 1/25,000 births, noted for its faciogenital dysplasia and mild neurobehavioral features. Assumpcao et al. (1999) reported autistic symptomotology as an additional feature in a subset of patients, making it a syndromic form of autism. This condition is due to a variety of mutations within the *FYVE, RhoGEF and PH Domain Containing 1* (*FGD1*) gene, which codes for a cell signaling protein that regulates the actin cytoskeleton by activating Cell Division Cycle 42 (Cdc42), a small Rho-GTPase vital for cell dynamics (Estrada et al., 2001).

Another cytoskeletally-related syndromic form of autism is Opitz G/BBB Syndrome, estimated to occur in 1–9/100,000 births. This syndrome appears to be due primarily to mutations in the *Midline 1* (*MID1*) gene, whose gene product has both structural and enzymatic functions. Structurally, it forms homodimers that associate with microtubules within the cytoplasm, acting as an anchor point for the microtubule network. Mid1 appears to be a necessary player throughout the development of the central nervous system: it's expressed within neural crest cells and is necessary for neural tube closure, it's expressed within the proliferating ventricular zone of the brain, and is even necessary for axonal guidance (Berti et al., 2004; Thomas et al., 2008; Lu et al., 2013).

There are many more genes in the core set, both syndromic and non-syndromic, necessary for proper cytoskeletal function in the developing nervous system. As we have reviewed here, one can begin to see the complex interplay between the structural network and cell fate determination. Not only does the cytoskeletal network help to carry out changes in cell fate, it also provides continuous feedback and can easily divert cellular development down a different path. Thus, though their roots may diverge, different gene mutations may ultimately overlap to produce similar disturbances in neuronal development and neurobehavioral symptomotology, as seen in both Aarskog-Scott and Opitz G/BBB Syndromes.

### Neural induction and differentiation are product-dependent

Additional effectors of induction and differentiation involve the regulation of neurotrophic factor activity, intracellular signaling transduction, chromatin remodeling, gene transcription, and protein translation. Each of these groups of molecules plays important roles in the regulation of what is known as *neural competency* or the readiness of a neural progenitor to undergo neurogenesis and eventually to differentiate into a mature neuron (Storey, 2003). Some of these factors are expressed in cell-specific manners, while others are universal. Brain Derived Neurotrophic Factor (BDNF) is an excellent example of a growth factor that is widely used throughout the central nervous system and the gene is also present within the core autism list studied here. When overexpressed, in conjunction with Epidermal Growth Factor (EGF) availability, BDNF promotes an increase in neurogenesis in a Phosphatidylinositol-4,5-bisphosphate 3-kinase (PI3K)/Akt-dependent manner *in vitro* (Zhang et al., 2011). Likewise, its ectopic expression in radial glia *in vivo* leads to premature neurogenesis and laminar maldistribution (Ortega and Alcántara, 2009). Overexpression of the core gene, *NTF4*, which codes for Neurotrophic Factor 4, results in further neural lineage commitment by NSCs, an effect which, like BDNF, lies upstream of PI3K/Akt activity (Shen et al., 2010). Akt also phosphorylates and sequesters Glycogen Synthase Kinase 3-β (GSK3β) away from β-catenin, allowing upregulation of the canonical Wnt pathway and the normal procession of neurogenesis. Similarly, the autism- and schizophrenia-associated intracellular signaling molecule, Disrupted in Schizophrenia 1 (DISC1), inhibits GSK3β activity through its N-terminal domain, thereby upregulating the Wnt pathway (Ming and Song, 2009). Interestingly, loss-of-function mutations in either of the core genes, *Chromodomain Helicase DNA Binding Protein 7* (*CHD7*) or *CHD8*, leads to derepression of Wnt; meanwhile, *Phosphatase and Tensin Homolog* (*PTEN*) and *Tuberous Sclerosis 1/2* (*TSC1/2*) mutations lead directly to the upregulation of PI3K/Akt pathway activity (Nishiyama et al., 2012; Chen et al., 2014). Together, this evidence suggests that regulation of cell growth, well-timed mitosis, and maturation are important factors in many forms of autism risk.

While neurotrophic factors lie upstream of gene activation, the epigenome regulates the shape and therefore the accessibility of target genes for transcription. In order for transcription to occur, the euchromatin must adopt an open conformation such that it may be competent to interact with the machinery necessary for transcription initiation (Pazin and Kadonaga, 1997). Without such open conformations, target genes remain unavailable regardless of the neurotrophic or transcription factors present. Thus, without the well-timed cooperation of the epigenome, changes to transcription, such as those that underlie the shift to neurogenesis, cannot occur properly (Ballas et al., 2005, for example). Chromatin regulators present within the core set include not only the *CHD7* and *CHD8* genes as mentioned above, but also other notable syndromic autism genes such as *AT Rich Interactive Domain 1B* (*ARID1B*) and *Methyl-CpG Binding Domain Protein 5* (*MBD5*) which are associated with autosomal dominant forms of intellectual disability, *Methyl CpG Binding Protein 2* (*MECP2*) whose loss-of-function mutations predispose toward Rett's Syndrome, *Nipped-B Homolog* (*NIPBL*) which is associated with Cornelia de Lange Syndrome type 1, and *Nuclear Receptor Binding SET Domain Protein 1* (*NSD1*), the causal gene in Sotos Syndrome (see OMIM database).

Ubiquitination of proteins is also a vital element in epigenetic regulation. In particular, polyubiquitination of the master regulator and repressor, Re-1 Silencing Transcription Factor (REST), leads to the de-repression of neuronal genes and subsequently to neural induction and differentiation (Stegmüller and Bonni, 2010). In its unubiquitinated state, REST associates with Mammalian Sin3 (mSin3) and REST Corepressor 1 (CoREST), recruiting the chromatin-associated proteins, Histone Deacetylases (HDAC), to neural-specific genes. Thus, when ubiquitination is impeded such as in a disorder of the same, REST may continue to suppress neuronal genes, leading to delayed induction (Stegmüller and Bonni, 2010). A perfect example of this lies in Angelman Syndrome (AS). AS is a form of intellectual disability that often presents with autistic symptomology. Maternally-inherited mutations in the *Ubiquitin Protein Ligase E3A* (*UBE3A*) gene, whose gene product is an integral part of the ubiquitin-induced protein degradation system, are the primary cause of this condition: in 25% of cases, mutation to the *UBE3A* gene itself are causal, meanwhile the vast majority of patients display larger chromosomal deletions that encompass *UBE3A* in addition to other genes (OMIM, 2012). While research is unfortunately sparse on the topic of neurogenesis in AS, one study by Mardirossian et al. (2009) did show that, while neuronal proliferation in the hippocampus did not appear to be affected in mouse models of the same, expression of mature neuronal markers (e.g., NeuN) was markedly reduced, indicating perturbations to successful maturation. Other research suggests that *UBE3A* plays an important role at the centrosome, with its levels peaking at the stage of mitosis, and is vital in chromosomal segration and nuclear kinesis (Singhmar and Kumar, 2011). As will be discussed later, models of AS also exhibit disturbances to neurite extension and plasticity.

The overall regulation of transcription and translation is a prime means to maintain homeostasis within a cell. Therefore, in order to induce change, target transcription and translation must occur (Storey, 2003). For transcription, not only must target genes be made conformationally available through epigenomic changes as discussed, the transcription factors themselves must be produced and readily available within the nucleus in order to bind to their targets. Thus, gene mutations that target transcription or function of these factors can lead to similar dysfunctions as noted in disorders of acetylation or methylation, ultimately leading to impaired transcription of target genes (Miller et al., 1999; Nishiyama et al., 2012). However, aside from transcription, it has more recently been acknowledged that the regulation of translation is vital for maintaining cell homeostasis. Eukaryotic Initiation Factors (eIF), for instance, are highly conserved positive and negative regulators of translation, and their behaviors are often dependent upon specific binding partners. While eIF4AII is a key inducer of neural competence in the embryonic neuroectoderm in *Xenopus* via the upregulation of translation, eIF4E in humans binds with both Fmr1 and Cyfip1 to form a complex that instead inhibits protein translation (Storey, 2003; Napoli et al., 2008). These regulators behave in a graded fashion such that when a complex suppresses translation, the cell requires greater numbers of neural inducing factors in order to overcome that suppression and acquire a neuronal fate (Storey, 2003). Therefore, in a condition such as FXS, in which *Fragile X Mental Retardation* (*FMR1*), *EIF4E*, or *Cytoplasmic FMR1-interacting Protein 1* (*CYFIP1*) are mutated, the complex's capacity for translation suppression is reduced and precocious induction and differentiation occur due to the lower threshold requirement that gradients of neural inducing factors must achieve (Storey, 2003; Castrén et al., 2005). As such precocious induction leads to disturbed maturation in FXS and increased risk for autism.

### Neural induction and differentiation are stress-sensitive

A novel concept, termed *hormesis*, has emerged over the last few decades that deals with the refractory or compensatory nature of cellular growth following stress or insult (Naviaux, 2014). Such stresses tend to produce mismatch between resource availability and metabolic requirements, and are rapidly followed by increased purinergic signaling and the production of reactive oxygen species (ROS) and Krebs cycle intermediates. The cell responds with the activation of anti-inflammatory and regenerative pathways, the latter which appears to include the downregulation of the KCC2 chloride exporter in neurons and the reacquisition of GABA once again as an excitatory neurotransmitter (Kim et al., 2011; Naviaux, 2014).

Several syndromic forms of autism are rooted in oxidative and mitochondrial dysfunction. Pyridoxine-dependent epilepsy (EPD), for instance, is due to mutations in the *Aldehyde Dehydrogenase 7 Family, Member A1* (*ALDH7A1*) gene whose gene product metabolizes methyl donors and various aldehydes thereby protecting against oxidative stress (GeneCards, 2014a). EPD is characterized by a variety of seizure types, all typically resistant to anticonvulsant therapy but responsive to pyridoxine, a form of vitamin B6. The vast majority of those with EPD also exhibit developmental delay, including psychomotor and language retardation. A minority of children also develop autism which, like the epilepsy, often responds to pyridoxine treatment in effected individuals (Mills et al., 2010). Interestingly, as a group those with idiopathic autism have increased rates of mitochondrial mutations, an indicator of mitochondrial stress and ROS-mediated damage (Napoli et al., 2013).

To understand what such metabolic and ROS-mediated dysfunction may be doing to the developing brain, one need only look to the bourgeoning literature on the subject. For instance, mitochondrial disturbances have clear effects on adult neurogenesis within the hippocampus, suppressing proliferation and reducing total neuroblast numbers (Calingasan et al., 2008). Similarly, inflammation, driven by microglia, astrocytes, and macrophages, can have a range of consequences on cell growth, proliferation, and repair depending upon the precise cocktail of pro-inflammatory and anti-inflammatory factors released into the local environment. A recent article by Le Belle et al. (2014) illustrates this well: the authors exposed wildtype mouse pups on embryonic day 9 to lipopolysaccharide (LPS), a molecule normally present on the surfaces of gram-negative bacteria that elicits a strong immune response in animals. Not only did the pups display postnatal megalencephaly as compared to vehicle-exposed pups, cortical thickness was increased and greater numbers of neocortical Nestin+ progenitors incorporated bromodeoxyuridine, indicating increased rates of proliferation. The authors went on to perform the identical experiment on a mouse model for autism, the *Pten* heterozygous knockout. Results were similar in trend to the wildtype-exposed mice, however the *Pten* response was exponentially exaggerated as compared to wildtype-exposed mice and *Pten* mice exposed to vehicle alone. Not only does this indicate the potential importance of prenatal infection in the development of a phenotype linked with autism (i.e., cerebral hyperplasia), it also shows how genetic susceptibility (e.g., *Pten* knockout), together with environmental exposure can supply an exponential, not just additive, effect on outcome.

In general, acute inflammation tends to have a stimulatory effect on neurogenesis, meanwhile, chronic inflammation suppresses it (Whitney et al., 2009). One therefore might wonder whether prenatal disturbances to the different arms of the cellular stress pathways could lead to overlapping phenotypes, resulting in autism in a subset of patients. Hopefully future research will address this question. This area of research, in particular, also holds considerable hope for treatment intervention and prevention given that the nature of the causal influences may be partly environmental.

### How perturbations in neuronal maturation may affect neural networks in autism

Studies utilizing MRI have reported a range of findings in autism, from largescale underconnectivity to local overconnectivity, as well as other mixed results (Maximo et al., 2014). *In vitro* and animal models of autism, both syndromic and non-syndromic alike, also display an array of findings dependent upon the model studied, including various alterations to neuritic length, branching complexity, and synaptic density (Ramocki and Zoghbi, 2008). While we can't address in this paper how disturbances to early neuronal maturation might lead to such a broad range of connectivity patterns and ultimately how those patterns overlap to produce the neurobehavioral phenotype known as autism, we can however provide evidence that illustrates how neuronal induction and differentiation are tightly linked processes.

As reviewed earlier, higher ratios of the chloride importer, NKCC1, to that of the exporter, KCC2, are features common to neural progenitors and neuroblasts. In addition, higher levels of NKCC1 postnatally may also be an indicator of pathology in both autism and epilepsy, leading to GABA-induced depolarization and poorly-restrained pyramidal cell excitation. Knockdown of NKCC1 in neural progenitors in the subventricular zone of mice results in reduced GABA_*A*_-induced depolarization and significant decreases in the number of proliferative Ki67+ progenitors and neuronal density, indicating the importance of excitatory activity in the progenitor population and their progeny (Young et al., 2012). Knockdown in the same cells also produces neurons with truncated dendritic arbors at the time of synaptic integration. Though by 6 weeks Young et al. report partial recovery in dendritic complexity, dendritic length was permanently altered. This suggests that neuritic elongation is a separate though overlapping process from that of branching and is comparably less plastic, which previous research has shown to be the case (Goldberg, 2004). This study also suggests that early cell fate determination may have a cascading effect on later stages of neuronal development. This is also complicated by the fact that many gene products are reused throughout various stages; therefore, when a single gene is mutated targeting some or all of its transcript variants, the effects may reverberate throughout the life of the neuron.

Another good example to illustrate how induction and early differentiation are linked is a form of XLMR caused by loss-of-function mutations in the *Ubiquitin Specific Peptidase 9, X-linked* (*USP9X*) gene. Though the condition appears quite rare, Homan et al. (2014) reported that 1/3rd of the individuals studied displayed distinctive autistic features, potentially making it a significant form of syndromic autism. The gene product itself is a deubiquitinase involved in protein degradation and turnover, as well as regulating pathway activities dependent upon monoubiquitination signals (GeneCards, 2014b). As such, it affects a wide array of cellular processes at all stages of development. Jolly et al. (2009), for instance, found that when Usp9x was overexpressed in neural progenitors in mouse, self-renewal was enhanced leading to a fivefold increase in progenitor and neuronal numbers. In contrast, Homan et al. studied *Usp9x* murine knockout and though they failed to address disturbances in neurogenesis, they did however find that knockout resulted in a significant reduction in axonal growth and severe neuronal migrational disturbances. Once again, this highlights the linkage between neurogenesis and neuritogenesis and how impairment in one, such as occurs in autism, often coincides with impairment in the other.

Dual-specificity Tyrosine-(Y)-phosphorylation Regulated Kinase 1A (Dyrk1A) is a protein kinase that targets serine and threonine residues for phosophorylation, and though its exact functions are not yet well understood, its substrates include a variety of transcription factors, splicing factors, and even eukaryotic initiation factors (Park et al., 2009). Most importantly, its overexpression is the most frequent cause of Down's Syndrome and comorbid syndromic autism and, like Ups9x, is involved in numerous stages of neuronal development. Yabut et al. (2010) report that the protein's overexpression leads to inhibition of neural progenitor proliferation and induces premature neurogenesis. Studies in *Drosophila melanogaster* likewise concur that it is an essential effector in postembryonic neurogenesis in the fruitfly (Park et al., 2009). And, in keeping with the topic of this review, Dyrk1A overexpression leads to disturbances in neuronal morphogenesis: while fetuses and newborns with Down's display apparently normal or even increased dendritic branching, by adulthood that branching complexity is severely reduced (Martinez de Lagran et al., 2012).

Like Down's Syndrome, those with idiopathic autism show a reduction in the overall size of the corpus callosum, the tract of white matter connecting the two cerebral hemispheres (Teipel et al., 2003; Casanova et al., 2011). This finding is also tightly positively correlated with gyral window size, the aperture through which all cortical efferent and afferent fibers pass (Casanova et al., 2009). These findings agree well with the largescale underconnectivity reported in autism. Specifically, MRI reports of disturbances to connectivity in the condition indicate abnormalities in the axonal compartment of neurons, as current imaging techniques are incapable of acquiring information on the unmyelinated dendritic comparment. At the cellular level one would therefore expect to see correlates reflective of these disturbances and a number of studies suggest that this is indeed the case (Choi et al., 2008; Tessier and Broadie, 2008; de Anda et al., 2012, for examples). Surprisingly, however, the dendritic compartment has received much more research attention in spite of the MRI studies. Results of molecular and cellular research suggest that all neuritic compartments, both axonal and dendritic, are affected across the broad spectrum of autism, though specific phenotypes may vary according to causation (Ramocki and Zoghbi, 2008, for examples).

The above examples cover a wide breadth of disturbances, from chloride importer/exporters to deubiquitinases to intracellular signaling molecules, yet they nevertheless converge to produce neurogenic and neuritogenic disturbances across the different forms of autism. This can be seen both at the cellular and macroscopic levels and includes: megalencephaly/microcephaly, signs of disturbed neurogenesis, altered cortical thickness, gyral distortions, dysplastic formations, ectopias and heterotopias, changes to white matter volume and functional connectivity, and altered neurite elongation and branching complexity (Fombonne et al., 1999; Hardan et al., 2006; Just et al., 2007; Williams et al., 2012). Ultimately, this broad range of disturbances leads to autistic symptomology in a significant subset of individuals. Yet why penetrance for genes with even the strongest of associations fails to produce autism in all cases is still a partial mystery, and undoubtedly reflects the complex etiology of the condition. However, though there are still many mysteries to solve in autism, hopefully we have illustrated how neural induction and differentiation are closely linked processes that are both highly activity-dependent, structurally-dependent on the dynamism of the underlying cytoskeleton and adhesive complexes, and heavily product-dependent in terms of transcription initiation and translation of those factors that promote maturation (Table 1B). In addition, they are also stress-sensitive, as can be seen with some mitochondrial and immune disorders or prenatal infections. Ultimately, each of these larger modules converge to produce a healthy, mature neuron—or, in the case of autism, neurons whose maturations have been hindered or redirected to an atypical phenotype, potentially preventing their proper integration into a larger coherent network of cells.

## Discussion

*“The overlap of neurodevelopmental and psychiatric phenotypes (such as mental retardation, epilepsy, autism, and other abnormal behaviours) that results from either loss or gain of the same proteins or RNA molecules supports an emerging theme that normal cognition and behaviour depend on tight neuronal homeostatic control mechanisms”* (Ramocki and Zoghbi, 2008, *p. 916*).

The above quote touches on the unusual fact that even though conditions such as FXS, AS, and Rett's Syndrome (RTT) overlap neurobehaviorally, their morphological and physiological phenotypes can be distinctly divergent. For instance, while FXS neurons exhibit normal dendrites and increased density of dendritic spines, AS neurons have normal dendrites and decreased density of dendritic spines; meanwhile, RTT neurons display a reduction in both dendrites and spines (Ramocki and Zoghbi, 2008). Yet, curiously, each of these conditions is often comorbid with autism. And as Ramocki and Zoghbi suggest, what these phenotypes may share in common is the impairment in neural network integration. Other authors of late have touched on similar themes: Auerbach et al. (2011), for instance, reported that mouse models of FXS and Tuberous Sclerosis (TSC) exhibit synaptic dysfunction that falls at opposite ends of a physiological spectrum, displaying divergent trends in longterm synaptic depression (LTD)-related protein synthesis in hippocampus. The authors conclude that their “findings reveal that even genetically heterogeneous causes of [autism] and intellectual disability may produce similar deficits by bidirectional deviations from normal on a common functional axis” (p. 67).

While the present authors would hesitate to use the term *homeostasis* as proposed by Ramocki and Zoghbi (2008) to describe the above events, there does appear to be a *common threshold of vulnerability* that is surpassed in each of these syndromic cases. What that physiological and/or morphological threshold may be is unknown, although disturbances in cell identity, migration, neuritic, and synaptic morphology and physiology all indicate that the ways in which these cells communicate with one another is probably markedly impaired. How that impairment leads specifically to autism symptomology requires further study however.

Yet we would extend Ramocki and Zoghbi's (2008) framework beyond the neurite and synapse to all stages of neuronal development and ground this work in neuropathological data common to both the idiopathic and syndromic forms. Though it is likely true that behavioral symptoms are ultimately manifest from disturbances in network communication, as it is the relationship amongst cells that defines brain function, the root of dysfunction is not necessarily relegated to the communicative arbors alone but appears to reach back well into the early development of the newborn neuron and is susceptible to disturbances at numerous points within a vast molecular network. The capacity for maturational regression of neuronal populations also suggests that all neurons, under select circumstances, have the propensity to develop a similar phenotype to those whose developments were perturbed earlier in prenatal development, though further work on regression is required in order to better understand these underlying pathological processes. But, ultimately, within an organ that contains incredible regional diversity, innumerable structural and molecular boundaries, and considerable circuit specificity, small effects to fundamental regulators of cellular identity can lead to pervasive malformation and extensive physiological dysfunction.

In this review, we have summarized evidence indicating that the core set of idiopathic and syndromic autism risk genes functionally overlaps at stages of neural induction and early maturation of the neuron. The majority of these same gene products, however, continue to serve roles in later stages of neuronal differentiation, linking common findings in the cytoarchitectural neuropathology of the conditions with molecular and functional studies that indicate disturbances to connectivity and synapse function. Because each developmental stage lies foundational to the next, modules, such as calcium signaling, cytoskeletal remodeling, and translation regulation, can target numerous downstream stages of growth and differentiation. As such, when disturbances are observed in one aspect of neuronal development, such as branching complexity, it may be reflective of a deeper fundamental disturbance to neuronal maturation. Thus, the researcher may need to look to earlier stages of the neuron's history to better understand the origin of the observed phenotype.

This review also highlights the genetic relationship, as well as the neuropathological one, between autism and epilepsy. We find that 63% of the core set shares at least modest overlap with known risk genes for epilepsy. In addition, not only do approximately 26% of cases of autism display comorbid seizure disorder, the two conditions share similar dysgenic underpinnings in the form of dysplasias, heterotopias, and ectopias. Finally, they both express molecular indicators of neuronal immaturity by way of progenitor-like ratios of chloride importer/exporters. Taken together, this evidence suggests that autism and epilepsy share very similar origins.

The genetics of schizophrenia show comparatively less overlap with the autism core set; however, the percentage is still considerable at 23%. Interestingly, although the majority of overlapping genes are likewise indicated in seizure etiology, there appears to be little in the way of diagnostic comorbidity between schizophrenia and epilepsy. However, in spite of the dearth of evidence, schizophrenic brains nevertheless exhibit similar markers of neuronal immaturity (e.g., NKCC1) within neocortex and hippocampus as seen in autism and epilepsy (Hyde et al., 2011). Walton et al. (2012) reported additional features of immaturity within the dentate gyrus in schizophrenia, including increased numbers of calretinin-positive progenitors, decreased calbindin-positive neurons, and increased neurogenesis, all indicators of the perturbation of neuronal maturation. At the cytoarchitectural level, decreased cortical thickness has been reported in the more severe childhood- and adolescent-onset forms of schizophrenia, a finding that is reminiscent of the dysplastic cortical thinning seen in autism and epilepsy as well (White et al., 2003; McDonald et al., 2008; Casanova et al., 2013). Therefore, although schizophrenia and epilepsy are rarely comorbid, they do appear to share similarities at the developmental level. Further research may help us understand both the convergent and divergent features of autism, epilepsy, and schizophrenia, providing better criteria by which to differentiate their origins as well as to understand shared risk factors.

We believe this is the first publication to view the broad spectrum of autism genetics through the lens of neuropathological data. As such, we hope that it provides a framework in which an extensive range of research interests may finally find common ground. The culmination of research to date suggests that, though later stages of differentiation are indeed disturbed in autism, encompassing neurite and synapse formation and function, the genesis of these features is generally rooted in even earlier stages of neuronal development and are the result of deviations in cellular identity. Therefore, investigative efforts focusing more holistically on all stages of neuronal development in autism, from progenitor expansion to plasticity, may prove more fruitful than the developmental and morphological compartmentalization that is currently en vogue.

## Author contributions

As primary author, Emily L. Casanova wrote the manuscript and provided background in genomics and embryology. Manuel F. Casanova provided expertise and guidance in understanding the neuropathologies of autism, epilepsy, and schizophrenia.

### Conflict of interest statement

The authors declare that the research was conducted in the absence of any commercial or financial relationships that could be construed as a potential conflict of interest.
