# Supplementary material for: Genetics studies indicate that neural induction and early neuronal maturation are disturbed in autism
Source: Front Cell Neurosci. 2014 Nov 19;8:397. doi: 10.3389/fncel.2014.00397 (PMC4237056; doi:10.3389/fncel.2014.00397)
Supplement: Supplementary file 1 [file Table1.PDF]

Supplementary Materials
(Suppl. Tables 1-3)

Supplementary Table 1. Citations for justification of ratings for each high-risk autism gene.

Aut Gene	Rating	Refs for Rating	Refs for Rating	Function in Neurite or Synapse
ACSL4	3	Cho (2012)	Zhang et al. (2009)	Meloni et al., (2009)
ADNP	3	Helsmoortel et al. (2014)		Pacual & Guerri (2007)
ADSL	2	Leivo et al. (2005)	Ng et al. (2009)	
AFF2	3	Wittwer et al. (2001)		Gu & Nelson (2003)
AGTR2	3	Li et al. (2007)	Park & Zambidis (2009)	Maul et al. (2008)
AHI1	3	Weng et al. (2013)		Doering et al. (2008)
ALDH5A1	3	Tozuka et al. (2005)		Vardya et al. (2010)
ALDH7A1	2	Moreb (2008)	Mills et al. (2010)	
AP1S2	2	Montpetit et al. (2008)	Kametaka et al. (2012)	Boehm & Bonifacino (2002)
ARHGEF6	2	Förster (2014)		Nodé-Langlois et al. (2006)
ARID1B	3	GeneCards (2014a)		Vogel-Ciernia et al. (2013)
ARX	3	Friocourt et al. (2008)		Yoshihara et al. (2005)
ASMT	2	Baka et al. (2010)		Melke et al. (2008)
ATP13A4	2	Vallipuram et al. (2010)		
ATRX	3	Bérubé et al. (2005)	Bérubé et al. (2002)	Shioda et al. (2011)
AUTS2	3	Oksenberg et al. (2013)		
AVPR1A	1	GeneCards (2014b)		Pagani et al. (2014)
BCL2	3	Zhang et al. (1996)		Rolland & Conradt (2010)
BDNF	3	Ahmed et al. (1995)		An et al. (2008)
BRAF	3	Raabe et al. (2011)	Maddodi et al. (2010)	Pfeiffer et al. (2013)
BTD	2	Valenciano et al. (2002)		Pindolia et al. (2012)
C4B	2	Shinjyo et al. (2009)		
CACNA1C	3	Paşca et al. (2011)		Moosmang et al. (2005)
CACNA1E	2	Gu & Spitzer (1997)		
CACNA1F	2	Gu & Spitzer (1997)		Thibault et al. (2001)
CACNA1H	2	Gu & Spitzer (1997)		Chemin et al. (2002)
CADM1	2	Ito et al. (2011)	Pietri et al. (2008)	Hagiyama et al. (2009)
CASK	2	Najm et al. (2008)	Pietri et al. (2008)	Wang et al. (2008)
CDH10	1	Takeichi (1991)		Barber et al. (2011)
CDH9	1	Takeichi (1991)		Williams et al. (2011)
CDKL5	3	Valli et al. (2012)		Zhu et al. (2013b)

CEP290	2	Senocak et al. (2010)		Coppieters et al. (2010)
CHD7	3	Layman et al. (2009)		Melicharek et al. (2010)
CHD8	3	Nishiyama et al. (2012)	Otero et al. (2004)	
CHRNA4	2	Mohapel et al. (2005)		Didier et al. (1995)
CHRNB2	2	Mohapel et al. (2005)		Sun et al. (2008)
CNTN4	2	Hansford et al. (2003)		Mercati et al. (2013)
CNTNAP2	3	Peñagarikano et al. (2011)	Strauss et al. (2006)	Fujita et al. (2012)
COMT	2	Rowe et al. (1993)	Laifenfeld et al. (2002)	Forero et al. (2006)
CREBBP	3	Sharma et al. (2010)		Sharma et al. (2010)
CYFIP1	3	Napoli et al. (2008)	Castrén et al. (2005)	Napoli et al. (2008)
DBH	2	Rowe et al. (1993)	Laifenfeld et al. (2002)	Gustafson & Moore (1987)
DCX	3	Brown et al. (2003)	Jin et al. (2010)	Shmueli et al. (2001)
DHCR7	3	Yu & Patel (2005)	Whitehead & Cobo (2008)	Yu et al. (2005)
DISC1	3	Mao et al. (2009)		Kamiya et al. (2006)
DMD	3	Deng et al. (2009)		Montañez et al. (2004)
DMPK	1	Marteyn et al. (2011)	Tang et al. (2012)	Schulz et al. (2003)
DNAH5	1	Olbrich et al. (2002)		
DRD3	2	Rowe et al. (1993)		Andersen & Sonntag (2014)
DRD4	2	Rowe et al. (1993)		Kwon et al. (2008)
DYRK1A	3	Yabut et al. (2010)		Martinez de Lagran et al. (2012)
EHMT1	2	GeneCards (2014c)	Thambirajah et al. (2012)	Kramer et al. (2011)
EIF4E	3	GeneCards (2014d)		Hashimoto & Ishima (2010)
EN2	2	Sarnat et al. (2002)	Cosgaya et al. (1998)	Brunet et al. (2005)
FBXO33	1	Guo et al. (2014)		
FEZF1	3	Shimizu et al. (2010)		Watanabe et al. (2009)
FEZF2	3	Shimizu et al. (2010)		Rouaux & Arlotta (2010)
FGD1	3	Pasteris & Gorski (1999)	Cappello et al. (2006)	
FGFR2	3	Maric et al. (2007)		Schüller et al. (2008)
FHIT	2	Huber & Weiske (2008)		
FMR1	3	Castrén et al. (2005)	Moro et al. (2006)	Tucker et al. (2006)
FOXG1	3	Hanashima et al. (2004)		Polleux et al. (2007)
FOXP1	2	Rousso et al. (2012)		Palmesino et al. (2010)
FOXP2	3	Rousso et al. (2012)		Vernes et al. (2011)
FTSJ1	2	Freude et al. (2004)	Luo et al. (2010)	
GABRA4	3	Tozuka et al. (2005)		Duveau et al. (2011)
GABRA5	3	Tozuka et al. (2005)		Duveau et al. (2011)
GABRB1	3	Tozuka et al. (2005)		Duveau et al. (2011)
GABRB3	3	Tozuka et al. (2005)		Duveau et al. (2011)

GABRG3	3	Tozuka et al. (2005)		Duveau et al. (2011)
GAD1	3	Tozuka et al. (2005)		Chattopadhyaya et al. (2007)
GAMT	3	Ducray et al. (2007)		Braissant et al. (2001)
GATM	3	Ducray et al. (2007)		Braissant et al. (2001)
GRIA3	3	Suzuki et al. (2006)	Maric et al. (2000)	Sprengel (2006)
GRIK2	3	Joo et al. (2007)		Motazacker et al. (2007)
GRIN2B	3	Joo et al. (2007)		Dimassi et al. (2013)
GRIN3B	3	Joo et al. (2007)		Hensen et al. (2010)
GRIP1	3	Joo et al. (2007)		Hoogenraad et al. (2005)
GRPR	3	Walton et al. (2014)		Walton et al. (2014)
GSE1	0			
GSTM1	2	Richter & Kass (1991)	Salim et al. (2006)	Sobczak et al. (2014)
GUCY2D	0			
HLA-DRB1	0			
HOXA1	3	Martinez-Ceballos & Gudas (2008)		del Toro et al. (2001)
HRAS	3	Paquin et al. (2009)		Fivaz et al. (2008)
IGF2	3	Bracko et al. (2012)		Schmeisser et al. (2012)
IL1RAPL1	2	Gambino et al. (2007)	Deisseroth et al. (2004)	Valnegri et al. (2011)
IMMP2L	2	Xiao et al. (2013)	Maestrini et al. (2010)	Fogel et al. (2012)
IQSEC2	2	GeneCards (2014e)		Shoubridge et al. (2010)
ITGB3	3	Ma et al. (2010)	Azmitia (2001)	Whyte (2014)
KCND2	3	Schaarschmidt et al. (2009)		Kim & Hoffman (2012)
KCNMA1	2	Zhang et al. (2014)		Hebert et al. (2014)
KDM5C	3	Poeta et al. (2013)		Rosenfelder (2009)
KIAA0100	2	Bain et al. (2000)		
KIAA2022	2	Magome et al. (2013)		Van Maldergem et al. (2013)
KRAS	2	Uhrbom et al. (2002)		Stornetter & Zhu (2011)
L1CAM	3	Mikulak et al. (2012)		Schäfer & Frotscher (2012)
L2HGDH	1	Krell et al. (2011)		
LAMP2	1	Yasin et al. (2013)		
LRFN5	2	GeneCards (2014f)		Mah et al. (2010)
LRP1	3	Zilberberg et al. (2004)	Otero et al. (2004)	Yoon et al. (2013)
MACROD2	2	Corda & Di Girolamo (2003)	Sharifi et al. (2013)	
MAOA	2	Rowe et al. (1993)		Lee et al. (2005)
MAP2K1	3	Pang et al. (1995)	Jori et al. (2005)	GeneCards (2014g)
MAPK1	3	Qiu & Green (1992)		Sakai et al. (1999)

MAPK3	3	Qiu & Green (1992)		Song et al. (2005)
MARK1	2	Moroni et al. (2006)		Wu et al. (2011)
MBD5	2	Talkowski et al. (2011)		Camarena et al. (2014)
MECP2	3	Peddada et al. (2006)		Degano et al. (2009)
MED12	3	Wang et al. (2009)		Wang et al. (2009)
MEF2C	3	Li et al. (2008)		Barbosa et al. (2008)
MEGF11	1	Kay et al. (2012)		
MET	3	Kato et al. (2004)		Maina et al. (1997)
MID1	2	Mnayer et al. (2006)	Dal Zotto et al. (1998)	Lu et al. (2013)
MKKS	2	Kim et al. (2005)		
MSNP1AS	2	Kerin et al. (2012)	Zhu et al. (2013c)	Paglini et al. (1998)
NDP	3	McNeill et al. (2013)		
NF1	3	Nishi et al. (1991)		Patrakitkomjom et al. (2008)
NFIX	3	Heng et al. (2014)		Wang et al. (2010)
NHS	2	Sharma et al. (2009)		
NIPA1	2	Tsang et al. (2009)	Ueberham & Arendt (2013)	Chen & Cheng (2009)
NIPBL	2	Pistocchi et al. (2013)		
NLGN3	1	Shi et al. (2013)		Budreck & Scheiffele (2007)
NLGN4X	3	Shi et al. (2013)		Shi et al. (2013)
NPHP1	2	Jauregui et al. (2008)	Chizhikov et al. (2007)	Eley et al. (2008)
NRXN1	3	Zeng et al. (2013)		Nussbaum et al. (2008)
NRXN2	1	Zeng et al. (2013)		Craig & Kang (2007)
NSD1	3	Berdasco et al. (2009)		
NTF3	3	Fariñas et al. (1996)	Ghosh & Greenberg (1995)	Dijkhuizen et al. (1997)
NTF4	3	Shen et al. (2010)		Cohen et al. (1994)
OCRL	3	Ramirez et al. (2012)		Ramirez et al. (2012)
OPHN1	2	Rocas et al. (2013)		Fauchereau et al. (2003)
OTC	2	Sautin et al. (2007)		Qureshi et al. (1998)
OTX1	3	Frantz et al. (1994)		Weimann et al. (1999)
OXTR	3	Bakos et al. (2013)	Jafarzadeh et al. (2014)	Sala et al. (2011)
PAFAH1B1	3	Pawlisz et al. (2008)		Hirotsune et al. (1998)
PAH	3	Liss & Grümer (1966)	Hörster et al. (2006)	Li et al. (2010)
PCDH19	3	Biswas (2012)	Gao et al. (2001)	Garrett (2009)
PHF6	3	Zhang et al. (2013)	Wang et al. (2013a)	Zhang et al. (2013)
PHF8	3	Qiu et al. (2010)		Asensio-Juan et al. (2012)
PITX1	2	Heldring et al. (2012)		
POGZ	2	GeneCards (2014h)		
POMGNT1	2	Hehr et al. (2007)	Verrotti et al. (2010)	Abbott et al. (2006)
POMT1	2	Beltrán-Valero de Bernabé et al. (2002)	Verrotti et al. (2010)	Judas et al. (2009)

PQBP1	2	Li et al. (2013)	Sheen et al. (2010)	Wang et al. (2013b)
PRKCB	1	NCBI (2014)		
PRKX	3	Blaschke et al. (2000)		Blaschke et al. (2000)
PRSS12	1	Wolfer et al. (2001)		Mitsui et al. (2013)
PTCHD1	3	Ericson et al. (1997)	Schneider et al. (2001)	So et al. (2006)
PTEN	3	Lachyankar et al. (2000)		Lachyankar et al. (2000)
PTPN11	3	Gauthier et al. (2007)		Rosário et al. (2007)
RAB39B	2	Giannandrea et al. (2010)		Giannandrea et al. (2010)
RAI1	2	Seranski et al. (2001)		
RAPGEF4	3	Shi et al. (2006)		Penzes et al. (2011)
RBFOX1	3	Fogel et al. (2012)		Gehman et al. (2011)
RELN	3	Kim et al. (2002)	Tabata & Nakajima (2002)	Nichols & Olson (2010)
RIMS3	0			Kumar et al. (2010)
RNF135	2	Yoshiga et al. (2013)	Douglas et al. (2007)	
RPE65	2	Métrailler et al. (2013)		Koenekoop (2003)
RPGRIP1L	3	Spassky et al. (2008)		
RPL10	2	Dugani et al. (2010)		Chiocchetti et al. (2011)
RPS6KA3	3	Dugani et al. (2010)		Ammar et al. (2013)
SATB2	3	Gyorgy et al. (2008)	Savarese et al. (2009)	Zhang et al. (2012)
SBF1	2	Chaerkady et al. (2011)		Firestein & Cleary (2001)
SCN1A	2	Okamura & Shidara (1990)	Barber et al. (2011)	Reid et al. (2014)
SCN2A	1	Okamura & Shidara (1990)		
SEMA5A	3	Zhu et al. (2013a)		Schwamborn et al. (2004)
SEZ6L2	1	Anderson et al. (2012)		Kumar et al. (2008)
SGSH	3	Nurcombe et al. (1993)	Israsena et al. (2004)	Hantas-Ambroise et al. (1987)
SHANK2	2	Kim et al. (2009)		Grabrucker et al. (2011)
SHANK3	2	Schuetz et al. (2004)	Wang et al. (2014)	Grabrucker et al. (2011)
SLC22A9	1	Volk (2014)		
SLC25A12	2	Richter & Kass (1991)	Legagnol-Bestel et al. (2008)	Legagnol-Bestel et al. (2008)
SLC6A4	3	Page et al. (2009)		Daubert & Condron (2010)
SLC6A8	3	Ducray et al. (2007)		Braissant et al. (2001)
SLC9A6	2	Zhang-James et al. (2011)	Bergmann et al. (1991)	Ouyang et al. (2013)
SLCO1C1	3	Schlosser et al. (2002)	Honegger & Lenoir (1980)	Leonard & Farwell (1997)
SMC1A	2	Horsfield et al. (2007)	Frank & Tsai (2009)	
ST8SIA2	3	Amoureux et al. (2000)	Satin & Kitajima (2013)	Lee et al. (2011)
SUV420H1	3	Völkel & Angrand (2007)	Yu et al. (2009)	
SYN1	2	Horsfield et al. (2007)	Cai et al. (2003)	Paonessa et al. (2013)
TBR1	3	Bedogni et al. (2010)		Huang et al. (2014)
TBX1	3	Raft et al. (2004)		Forstner et al. (2013)

TRIO	2	Seipel et al. (1999)		Estrach et al. (2002)
TSC1	3	Meikle et al. (2007)	Sun et al. (2010)	Floricel et al. (2007)
TSC2	3	Soucek et al. (1998)		Nie et al. (2010)
TTN	1	Qi et al. (2008)		
TUBA1A	3	Fallet-Bianco et al. (2008)	Poirier et al. (2007)	Veldman et al. (2010)
TUBGCP5	2	Fallet-Bianco et al. (2008)		Baas & Joshi (1992)
UBE3A	3	Mardirossian et al. (2009)	Mishra et al. (2009)	Greer et al. (2010)
UPF3B	3	Jolly et al. (2013)		Jolly et al. (2013)
UPP2	2	GeneCards (2014j)	Silei et al. (2000)	Rooslid et al. (2011)
VIP	3	Pincus et al. (1990)		White et al. (2000)
VPS13B	2	Budisteanu et al. (2010)	Baker (2013)	
YWHAE	2	Toyo-oka et al. (2003)		Toyo-oka et al. (2003)
ZNF674	0			
ZNF81	0			

References for Supplementary Table 1

Abbott, K. L., Troupe, K., Lee, I., and Pierce, M. (2006). Integrin-dependent neuroblastoma cell adhesion and migration on laminin is regulated by expression levels of two enzymes in the O-mannosyl-linked glycosylation pathway, PomGnT1 and GnT-Vb. Exp. Cell Res. *312*, 2837-2850.

Acosta, R., Montañez, C., Fuentes-Mera, L., Gonzalez, E., Gómez, P., Quintero-Mora, L., Mornet, D., Alvarez-Salas, L. M., and Cisneros, B. (2004). Dystrophin Dp71 is required for neurite outgrowth in PC12 cells. Exp. Cell Res. *296*, 265-275.

Ahmed, S., Reynolds, B. A., and Weiss, S. (1995). BDNF enhances the differentiation but not the survival of CNS stem cell-derived neuronal precursors. J. Neurosci. *15*, 5765-5778.

Ammar, M. R., Humeau, Y., Hanauer, A., Nieswandt, B., Bader, M. F., and Vitale, N. (2013). The Coffin-Lowry syndrome-associated protein RSK2 regulates neurite outgrowth through phosphorylation of phospholipase D1 (PLD1) and synthesis of phosphatidic acid. J. Neurosci. *33*, 19470-19479.

Amoureux, M. C., Cunningham, B. A., Edelman, G. M., and Crossin, K. L. (2000). N-CAM binding inhibits the proliferation of hippocampal progenitor cells and promotes their differentiation to a neuronal phenotype. J. Neurosci. *20*, 3631-3640.

An, J. J., Gharami, K., Liao, G. Y., Woo, N. H., Lau, A. G., Vanevski, F., Torre, E. R., Jones, K. R., Feng, Y., Lu, B., et al. (2008). Distinct role of long 3’ UTR BDNF mRNA in spine morphology and synaptic plasticity in hippocampal neurons. Cell *134*, 175-187.

Andersen, S. L., and Sonntag, K. C. (2014). Juvenile methylphenidate reduces prefrontal cortex plasticity via D3 receptor and BDNF in adulthood. Front. Synaptic Neurosci. *6*, 1.

Anderson, G. R., Galfin, T., Xu, W., Aoto, J., Malenka, R. C., and Südhof, T. C. (2012). Candidate autism gene screen identifies critical role for cell-adhesion molecule CASPR2 in dendritic arborization and spine development. Proc. Natl. Acad. Sci. USA *109*, 18120-18125.

Asensio-Juan, E., Gallego, C., Martínez-Balbás, M. A. (2012). The histone demethylase PHF8 is essential for cytoskeleton dynamics. Nucleic Acids Res. *40*, 9429-9440.

Azmitia, E. C. (2001). Modern views on an ancient chemical: serotonin effects on cell proliferation, maturation, and apoptosis. Brain Res. Bull. *56*, 413-424.

Baas, P. W., and Joshi, H. C. (1992). Gamma-tubulin distribution in the neuron: implications for the origins of neuritic microtubules. JCB *119*, 171-178.

Bain, G., Mansergh, F. C., Wride, M. A., Hance, J. E., Isogawa, A., Rancourt, S. L., Ray, W. J., Yoshimura, Y., Tsuzuki, T., Gottlieb, D. I., et al. (2000). ES cell neural differentiation reveals a substantial number of novel ESTs. Funct. Integr. Genomics *1*, 127-139.

Baka, M., Uyanikgil, Y., Ateş, U., and Kültürsay, N. (2010). Investigation of maternal melatonin effect on the hippocampal formation of newborn rat model of intrauterine cortical dysplasia. Childs Nerv. Syst. *26*, 1575-1581.

Baker, K. (2013). Syndromes not yet proven to be ciliopathies. In Ciliopathies: A Reference for Clinicians. T. D. Kenny and P. L. Beales, eds. (Oxford: Oxford University Press), pp-262-274.?

Bakos, J., Strbak, V., Paulikova, H., Krajnakova, L., Lestanova, Z., Bacova, Z. (2013). Oxytocin receptor ligands induce changes in cytoskeleton in neuroblastoma cells. J. Mol. Neurosci. *50*, 462-468.

Barba, C., Parrini, E., Coras, R., Galuppi, A., Craiu, D., Kluger, G., Parmeggiani, A., Pieper, T., Schmitt-Mechelke, T., Striano, P., et al. (2014). Co-occurring malformations of cortical development and SCN1A gene mutations. Epilepsia *55*, 1009-1019.

Barber, J. C., Huang, S., Bateman, M. S., and Collins, A. L. (2011). Transmitted deletions of medial 5p and learning difficulties; does the cadherin cluster only become penetrant when flanking genes are deleted? Am. J. Med. Genet. A. *155A*, 2807-2815.

Barbosa, A. C., Kim, M. S., Ertunc, M., Adachi, M., Nelson, E. D., McAnally, J., Richardson, J. A., Kavalali, E. T., Monteggia, L. M., Bassel-Duby, R., et al. (2008). MEF2C, a transcription factor that facilitates learning and memory by negative regulation of synapse numbers and function. Proc. Natl. Acad. Sci. USA *105*, 9391-9396.

Bedogni, F., Hodge, R. D., Elsen, G. E., Nelson, B. R., Daza, R. A., Beyer, R. P., Bammler, T. K., Rubenstein, J. L., and Hevner, R. F. (2010). Tbr1 regulates regional and laminar identity of postmitotic neurons in developing neocortex. Proc. Natl. Acad. Sci. USA *107*, 13129-13134.

Beltrán-Valero de Bernabé, D., Currier, S., Steinbrecher, A., Celli, J., van Beusekom, E., van der Zwaag, B., Kayserili, H., Merlini, L., Chitayat, D., Dobyns, W. B., (2002). Mutations in the O-mannosylatransferase gene POMT1 give rise to the severe neuronal migration disorder Walker-Warburg syndrome. Am. J., Hum. Genet. *71*, 1033-1043.

Berdasco, M., Ropero, S., Setien, F., Fraga, M. F., Lapunzina, P., Losson, R., Alaminos, M., Cheung, N. K., Rahman, N., and Esteller, M. (2009). Epigenetic inactivation of the Sotos overgrowth syndrome gene histone methyltransferase NSD1 in human neuroblastoma and glioma. Proc. Natl. Acad. Sci. USA *106*, 21830-21835.

Bergmann, M., Lahr, G., Mayerhoffer, A., and Gratzl, M. (1991). Expression of synaptophysin during the prenatal development of the rat spinal cord: correlation with basic differentiation processes of neurons. Neuroscience *42*, 569-582.

Bérubé, N. G., Jagla, M., Smeenk, C., De Repentigny, Y., Kothary, R., and Picketts, D. J. (2002). Neurodevelopmental defects resulting from ATRX overexpression in transgenic mice. Hum. Mol. Genet. *11*, 253-261.

Bérubé, N. G., Mangelsdorf, M., Jagla, M., Vanderluit, J., Garrick, D., Gibbons, R. J., Higgs, D. R., Slack, R. S., and Picketts, D. J. (2005). The chromatin-remodeling protein ATRX is critical for neuronal survival during corticogenesis. J. Clin. Invest. *115*, 258-267.

Biswas, S. (2012). Role of protocadherins in the zebrafish neural development. Diss. Defense. (The Ohio State University.) Accessed on 8/28/2014 from http://gradworks.umi.com/35/35/3535233.html

Blaschke, R. J., Monaghan, A. P., Bock, D., and Rappold, G. A. (2000). A novel murine PKA-related protein kinase involved in neuronal differentiation. Genomics *64*, 187-194.

Boehm, M., and Bonifacino, J. S. (2002). Genetic analyses of adaptin function from yeast to mammals. Gene *286*, 175-186.

Bracko, O., Singer, T., Aigner, S., Knoblock, M., Winner, B., Ray, J., Clemenson, G. D., Jr., Suh, H., Couillard-Despres, S., Aigner, L., Gage, F. H., et al. (2012). Gene expression profiling of neural stem cells and their neuronal progeny reveals IGF2 as a regulator of adult hippocampal neurogenesis. J. Neurosci. *32*, 3376-3387.

Braissant, O., Henry, H., Loup, M., Eilers, B. and Bachmann, C. (2001). Endogenous synthesis and transport of creatine in the rat brain: an in situ hybridization study. Brain Res. Mol. Brain Res. *86*, 193-201.

Brown, J. P., Couilard, Després, S., Cooper-Kuhn, C. M., Winkler, J., Aigner, L. and Kuhn, H. G. (2003). Transient expression of doublecortin during adult neurogenesis. J. Comp. Neurol. *467*, 1-10.

Brunet, I., Weinl, C., Piper, M., Trembleau, A., Volovitch, M., Harris, W., Prochiantz, A., and Holt, C. (2005). The transcription factor Engrailed-2 guides retinal axons. Nature *438*, 94-98.

Budisteanu, M., Barca, D., Chirieac, S. M., and Magureanu, S. (2010). Cohen syndrome – a rare genetic cause of hypotonia in children. Maedica *5*, 56-61.

Budreck, E. C., and Scheiffele, P. (2007). Neuroligin-3 is a neuronal adhesion protein at GABAergic and glutamatergic synapses. Eur. J., Neurosci. *26*, 1738-1748.

Cai, X., Dong, F., Edelmann, R. E., and Makaroff, C. A. (2003). The *Arabidopsis* SYN1 cohesin protein is required for sister chromatid arm cohesion and homologous chromosome pairing. J. Cell Sci. *116*, 2999-3007.

Camarena, V., Cao, L., Abad, C., Abrams, A., Toledo, Y., Araki, K., Araki, M., Walz, K., and Young, J. I. (2014). Disruption of Mbd5 in mice causes neuronal functional deficits and neurobehavioral abnormalities consistent with 2q23.1 microdeletion syndrome. EMBO Mol. Med. *6*, 1003-1015.

Cappello, S., Attardo, A., Wu, X., Iwasato, T., Itohara, S., Wilsch-Bräuninger, M., Eilken, H. M., Rieger, M. A., Schroeder, T. T., Huttner, W. B., et al. (2006). The Rho-GTPase cdc42 regulates neural progenitor fate at the apical surface. Nat. Neurosci. *9*, 1099-1107.

Castrén, M., Tervonen, T., Kärkkäinen, V., Heinonen, S., Castrén, E., Larsson, K. Bakker, C. E., Oostra, B. A., and Akerman, K. (2005). Altered differentiation of neural stem cells in fragile X syndrome. Proc. Natl. Acad. Sci. USA *102*, 17834-17839.

Chaerkady, R., Letzen, B., Renuse, S., Sahasrabuddhe, N. A., Kumar, P., All, A. H., Thakor, N. V., Delanghe, B., Gearhart, J. D., Pandey, A., et al. (2011). Quantitative temporal proteomic analysis of human embryonic stem cell differentiation into oligodendrocyte progenitor cells. Proteomics *11*, 4007-4020.

Chattopadhyaya, B., Di, Cristo, G., Wu, C. Z., Knott, G., Kuhlman, S., Fu, Y., Palmiter, R. D., and Huang, Z. J. (2007). GAD67-mediated GABA synthesis and signaling regulate inhibitory synaptic innervation in the visual cortex. Neuron *54*, 889-903.

Chemin, J., Nargeot, J., and Lory, P. (2002). Neuronal T-type alpha 1H calcium channels induce neuritogenesis and expression of high-voltage-activated calcium channels in the NG108-15 cell line. J. Neurosci. *22*, 6856-6862.

Chen, S. Y., and Cheng, H. J. (2009). Functions of axon guidance molecules in synapse formation. Curr. Opin. Neurobiol. *19*, 471-478.

Chiocchetti, A., Pakalapati, G., Duketis, E., Wieman, S., Poustka, A., Poustka, F., and Klauck, S. M. (2011). Mutation and expression analyses of the ribosomal protein gene RPL10 in an extended German sample of patients with autism spectrum disorder. Am. J. Med. Genet. *155A*, 1472-1475.

Chizhikov, V. V., Davenport, J., Zhang, Q., Shih, E. K., Cabello, O. A., Fuchs, J. L., Yoder, B. K., and Millen, K. J. (2007). Cilia proteins control cerebellar morphogenesis by promoting expansion of the granule progenitor pool. J. Neurosci. *27*, 9780-9789.

Cho, Y. Y. (2012). A novel role of brain-type ACS4 isotype in neuronal differentiation. Biochem. Biophys. Res. Commun. *419*, 505-510.

Cohen, A., Bray, G. M., and Aguayo, A. J. (1994). Neurotrophin-4/5 (NT-4/5) increases adult rat retinal ganglion cell survival and neurite outgrowth in vitro. J. Neurobiol. *25*, 953-959.

Coppieters, F., Lefever, S., Leroy, B. P., and De Baere, E. (2010). CEP290, a gene with many faces: mutation overview and presentation of CEP290base. Hum. Mutat. *31*, 1097-1108.

Corda, D., and Di Girolamo, M. (2003). Functional aspects of protein mono-ADP-ribosylation. EMBO J. *22*, 1953-1958.

Cosgaya, J. M., Aranda, A., Cruces, J., and Martín-Blanco, E. (1998). Neuronal differentiation of PC12 cells induced by engrailed homeodomain is DNA-binding specific and independent of MAP kinases. J. Cell Sci. *111*, 2377-2384.

Craig, A. M., and Kang, Y. (2007). Neurexin-neuroligin signaling in synapse development. Curr. Opin. Neurobiol. *17*, 43-52.

Dal Zotto, L., Quaderia, N. A., Elliott, R., Lingerfelter, P. A., Carrel, L., Valsecchi, V., Montini, E., Yen, C. H., Chapman, V., Kalcheva, I., et al. (1998). The mouse Mid1 gene: implications for the pathogenesis of Opitz syndrome and the evolution of the mammalian pseudoautosomal region. Hum. Mol. Genet. *7*, 489-499.

Daubert, E. A., and Condron, B. G. (2010). Serotonin: a regulator of neuronal morphology and circuitry. Trends Neurosci. *33*, 424-434.

Degano, A. L., Pasterkamp, R. J., and Ronnett, G. V. (2009). MeCP2 deficiency disrupts axonal guidance, fasciculation, and targeting by altering Semaphorin 3F function. Mol. Cell Neurosci. *42*, 243-254.

Deisseroth, K., Singla, S., Toda, H., Monje, M., Palmer, T. D., and Malenka, R. C. (2004). Excitation-neurogenesis coupling in adult neural stem/progenitor cells. Neuron *42*, 535-552.

del Toro, E. D., Borday, V., Davenne, M., Neun, R., Rijli, F. M., and Champagnat, J. (2001). Generation of a novel functional neuronal circuit in Hoxa1 mutant mice. J. Neurosci. *21*, 5637-5642.

Deng, B., Glanzman, D., and Tidball, J. G. (2009). Nitric oxide generated by muscle corrects defects in hippocampal neurogenesis and neural differentiation caused by muscular dystrophy. J. Physiol. *587*, 1769-1778.

Didier, M., Bix, G., Berman, S. A., and Bursztajn, S. (1995). Expression of the alpha 4 neuronal nicotinic acetylcholine receptor subunit in the developing mouse hippocampus. Int. J. Dev. Neurosci. *13*, 703-713.

Dijkhuizen, P. A., Hermens, W. T., Teunis, M. A., and Verhaagen, J. (1997). Adenoviral vector-directed expression of neurotrophin-3 in rat dorsal root ganglion explants results in a robust neurite outgrowth response. J. Neurobiol. *33*, 172-184.

Dimassi, S., Andrieux, J., Labalme, A., Lesca, G., Cordier, M. P., Boute, O., Neut, D., Edery, P., Sanlaville, D.,and Schluth-Bolard, C. (2013). Interstitial 12p13.1 deletion involving GRIN2B in three patients with intellectual disability. Am. J. Med. Genet. *161*, 2564-2569.

Doering, J. E., Kane, K., Hsiao, Y. C., Yao, C., Shi, B., Slowik, A. D., Dhagat, B., Scott, D. D., Ault, J. G., Page-McCaw, P. S., and Ferland, R. J. (2008). Species differences in the expression of Ahi1, a protein implicated in the neurodevelopmental disorder Joubert syndrome, with preferential accumulation to stigmoid bodies. J. Comp. Neurol. *511*, 238-256.

Douglas, J., Cilliers, D., Coleman, K., Tatton-Brown, K., Barker, K., Bernhard, B., Burn, J., Huson, S., Josifova, D., et al. (2007). Mutations in RNF135, a gene within the NF1 microdeletion region, cause phenotypic abnormalities including overgrowth. Nat. Genet. *39*, 963-965.

Ducray, A. D., Schläppi, J. A., Qualls, R., Andres, R. H., Seiler, R. W., Schlattner, U., Wallimann, T., and Widmer, H. R. (2007). Creatine treatment promotes differentiation of GABA-ergic neuronal precursors in cultured fetal rat spinal cord. J. Neurosci. Res. *85*, 1863-1875.

Dugani, C. B., Paquin, A., Kaplan, D. R., and Miller, F. D. (2010). Coffin-Lowry syndrome: a rolle for RSK2 in mammalian neurogenesis. Dev. Biol. *347*, 348-359.

Duveau, V., Laustela, S., Barth, L., Gianolini, F., Vogt, K. E., Keist, R., Chandra, D., Homanics, G. E., Rudolph, U., and Fritschy, J. M. (2011). Spatiotemporal specificity of $GABA_A$ receptor-mediated regulation of adult hippocampal neurogenesis. Eur. J. Neurosci. *34*, 362-373.

Eley, L., Moochhala, S. H., Simms, R., Hildebrandt, F., and Sayer, J. A. (2008). Nephrocystin-1 interacts directly with Ack1 and is expressed in human collecting duct. Biochem. Biophys. Res. Commun. *371*, 877-882. Cell *90*, 169-180.

Ericson, J., Rashbass, P., Schedl, A., Brenner-Morton, S., Kawakami, A., van Heyningen, V., Jessell, T. M., and Briscoe, J. (1997). Pax6 controls progenitor cell identity and neuronal fate in response to graded Shh signaling.

Estrach, S., Schmidt, S., Diriong, S., Penna, A., Blangy, A., Fort, P., and Debant, A. (2002). The human Rho-GEF trio and its target GTPase RhoG are involved in the NGF pathway, leading to neurite outgrowth. Curr. Biol. *12*, 307-312.

Fallet-Bianco, C., Loeuillet, L., Poirier, K., Loget, P., Chapon, F., Pasquier, L., Saillour, Y., Beldjord, C., Chelly, J., and Francis, F. (2008). Neuropathological phenotype of a distinct form of lissencephaly associated with mutations in TUBA1A. Brain *131*, 2304-2320.

Fariñas, I., Yoshida, C. K., Backus, C., and Reichardt, L. F. (1996). Lack of neurotrophin-3 results in death of spinal sensory neurons and premature differentiation of their precursors. Neuron *17*, 1065-1078.

Fauchereau, F., Herbrand, U., Chafey, P., Eberth, A., Koulakoff, A., Vinet, M. C., Admadian, M. R., Chelly, J., and Billuart, P. (2003). The RhoGAP activity of OPHN1, a new F-actin-binding protein, is negatively controlled by its amino-terminal domain. Mol. Cell Neurosci. *23*, 574-586.

Fivaz, M., Bandara, S., Inoue, T., and Meyer, T. (2008). Robust neuronal symmetry breaking by Ras-triggered local positive feedback. Curr. Biol. *18*, 44-50.

Firestein, R., and Cleary, M. L. (2001). Pseudo-phosphatase Sbf1 contains an N-terminal GEF homology domain that modulates its growth regulatory properties. J. Cell. Sci. *114*, 2921-2927.

Floricel, F., Higaki, K., Maki, H., Nanba, E., Ninomiya, H., and Ohno, K. (2007). Antisense suppression of TSC1 gene product, hamartin, enhances neurite outgrowth in NGF-treated PC12h cells. Brain Dev. *29*, 502-529.

Fogel, B. L., Wexler, E., Wahnich, A., Friedrich, T., Vijayendran, C., Gao, F., Parikshak, N., Konopka, G., and Geschwind, D. H. (2012). RBFOX1 regulates both splicing and transcriptional networks in human neuronal development. Hum. Mol. Genet. *21*, 4171-4186.

Forero, D. A., Benítez, B., Arboleda, G., Yunis, J. J., Pardo, R., and Arboleda, H. (2006). Analysis of functional polymorphisms in three synaptic plasticited-related genes (BDNF, COMT and UCHL1) in Alzheimer's disease in Colombia. Neurosci. Res. *55*, 334-341.

Förster, E. (2014). Reelin, neuronal polarity and process orientation of cortical neurons. Neuroscience *269*, 102-111.

Forstner, A. J., Degenhardt, F., Schratt, G., and Nöthen, M. M. (2013). MicroRNAs as the cause of schizophrenia in 22q11.2 deletion carriers, and possible implications for idiopathic disease: a mini-review. Front. Mol. Neurosci. *6*, 47.

Frank, C. L., and Tsai, L. H. (2009). Alternative functions of core cell cycle regulators in neuronal migration, neuronal maturation, and synaptic plasticity. Neuron *62*, 312-326.

Frantz, G. D., Weimann, J. M., Levin, M. E., and McConnell, S. K. (1994). Otx1 and Otx2 define layers and regions in developing cerebral cortex and cerebellum. J. Neurosci. *14*, 5725-5740.

Freude, K., Hoffman, K., Jensen, L. R., Delatycki, M. B., des Portes, V., Moser, B., Hamel, B., van Bokhoven, H., Moraine, C., Fryns, J. P., et al. (2004). Mutations in the FTSJ1 gene coding for a novel S-adenosylmethionine-binding protein cause nonsyndromic X-linked mental retardation. Am. J. Hum. Genet. *75*, 305-309.

Friocourt, G., Kanatani, S., Tabata, H., Yozu, M., Takahashi, T., Antypa, M., Raguénès, O., Chelly, J., Férec, C., Nakajima, K., et al. (2008). Cell-autonomous roles of ARX in cell proliferation and neuronal migration during corticogenesis. J. Neurosci. *28*, 5794-5805.

Fujita, E., Tanabe, Y., Momoi, M. Y., and Momoi, T. (2012). Cntnap2 expression in the cerebellum of Foxp2(R552H) mice, with a mutation related to speech-language disorder. Neurosci. Lett. *506*, 277-280.

Gambino, F., Pavlowsky, A., Béglé, A., Dupont, J. L., Bahi, N., Courjaret, R., Gardette, R., Hadjkacem, H., Skala, H., Poulain, B., et al. (2007). IL1-receptor accessory protein-like 1 (IL1RAPL1), a protein involved in cognitive functions, regulates N-types Ca2+-channel and neurite elongation. Proc. Natl. Acad. Sci. USA *104*, 9063-9068.

Gao, X., Bian, W., Yang, J., Tang, K., Kitani, H., Atsumi, T., and King, N. (2001). A role of N-cadherin in neuronal differentiation of embryonic carcinoma P19 cells. Biochem. Biophys. Res. Commun. *284*, 1098-1103.

Garrett, A. (2009). Control of synaptogenesis and dendritic arborization by the ϒ-protocadherin family of adhesion molecules. Diss. Defense. (The University of Iowa). Accessed on 8/28/2014 from http://ir.uiowa.edu/etd/362/

Gauthier, A. S., Furstoss, O., Araki, T., Chan, R., Neel, B. G., Kaplan, D. R., and Miller F. D. (2007). Control of CNS cell-date decisions by SHP-2 and its dysregulation in Noonan syndrome. Neuron *54*, 245-262.

Gehman, L. T., Stoilov, P., Maguire, J., Damianov, A., Lin, C. H., Shiue, L., Ares, M., Jr., Mody, I., and Black, D. L. (2011). The splicing regulator Rbfox1 (A2BP1) controls neuronal excitation in the mammalian brain. Nat. Genet. *43*, 706-711.

GeneCards. (2014a). AT Rich Interactive Domain 1B (SWI1-Like). Accessed on 8/26/2014 from http://www.genecards.org/cgi-bin/carddisp.pl?gene=ARID1B&search=ARID1B

GeneCards. (2014b). Arginine Vasopressin Receptor 1A. Accessed on 8/26/2014 from http://www.genecards.org/cgi-bin/carddisp.pl?gene=AVPR1A&search=AVPR1A

Genecards. (2014c). Euchromatic Histone-Lysine N-Methyltransferase 1. Accessed on 8/27/2014 from http://www.genecards.org/cgi-bin/carddisp.pl?gene=EHMT1&search=EHMT1

GeneCards. (2014d). Eukaryotic Translation Initiation Factor 4E. Accessed on 8/27/2014 from http://www.genecards.org/cgi-bin/carddisp.pl?gene=EIF4E&search=EIF4E

GeneCards. (2014e). IQ Motif and Sec7 Domain 2. Accessed on 8/27/2014 from http://www.genecards.org/cgi-bin/carddisp.pl?gene=IQSEC2&search=IQSEC2

GeneCards. (2014f). Leucine Rich Repeat and Fibronectin Type III Domain Containing… Accessed on 8/27/2014 from http://www.genecards.org/cgi-bin/carddisp.pl?gene=LRFN5&search=LRFN5

GeneCards. (2014g). Mitogen-activated Protein Kinase Kinase 1. Accessed on 8/27/2014 from http://www.genecards.org/cgi-bin/carddisp.pl?gene=MAP2K1&search=5d645617d4626ea19e99af87bce79f87

GeneCards. (2014h). Pogo Transposable Element with ZNF Domain. Accessed on 8/28/2014 from http://www.genecards.org/cgi-bin/carddisp.pl?gene=POGZ&search=POGZ

GeneCards. (2014j). Uridine Phosphorylase 2. Accessed on 9/6/2014 from http://www.genecards.org/cgi-bin/carddisp.pl?gene=UPP2&search=3665af89ad8056ac5436a59892fd395b

Ghosh, A., and Greenberg, M. E (1995). Distinct roles for bFGF and NT-3 in the regulation of cortical neurogenesis. Neuron *15*, 89-103.

Giannandrea, M., Bianchi, V., Mignogna, M. L., Sirri, A., Carrabino, S., D'Elia, E., Vecellio, M., Russo, S., Cogliati, F., Larizza, L., et al. (2010). Mutations in the small GTPase gene RAB39B are responsible for X-linked mental retardation associated with autism, epilepsy, and macrocephaly. Am. J. Hum. Genet. *86*, 185-195.

Grabrucker, A. M., Knight, M. J., Proepper, C., Bockman, J., Joubert, M., Rowan, M., Nienhaus, G. U., Garner, C. C., Bowie, J. U., Kreutz, M. R., et al. (2011). Concerted action of zinc and ProSAP/Shank in synaptogenesis and synapse maturation. EMBO J. *30*, 569-581.

Greer, P. L., Hanayama, R., Bloodgood, B. L., Mardinly, A. R., Lipton, D. M., Flavell, S. W., Kim, T.-K., Griffith, E. C., Waldon, Z., Maehr, R., et al. (2010). The Angelman syndrome protein Ube3A regulates synapse development by ubiquitinating Arc. Cell *140*, 704-716.

Gu, Y., and Nelson, D. L. (2003). FMR2 function: insight from a mouse knockout model. Cytogenet. Genome Res. *100*, 129-139.

Gu, X., and Spitzer, N. C. (1997). Breaking the code: regulation of neuronal differentiation by spontaneous calcium transients. Dev. Neurosci. *19*, 33-41.

Guo, J., North, B. J., Tron, A. E., Inuzuka, H., and Wei, W. (2014). The role of FBXO subfamily of F-box proteins in tumorigenesis. In SCF and APC E3 Ubiquitin Ligases in Tumorigenesis. H. Inuzuka and W. Wei, eds. (Springer International Publishing), pp. 73-87.

Gustafson, E. L., and Moore, R. Y. (1987). Noradrenaline neuron plasticity in developing rat brain: effects of neonatal 6-hydroxydopamine demonstrated by dopamine-beta-hydroxylase immunocytochemistry. Brain Res. *465*, 143-155.

Gyorgy, A. B., Szemes, M., de Juan Romero, C., Tarabykin, V., and Agoston, D. V. (2008). SATB2 interacts with chromatin-remodeling molecules in differentiating cortical neurons. Eur. J. Neurosci. *27*, 865-873.

Hagiyama, M., Ichiyanagi, N., Kimura, K. B., Murakami, Y., and Ito, A. (2009). Expression of a soluble isoform of cell adhesion molecule 1 in the brain and its involvement in directional neurite outgrowth. Am. J. Pathol. *174*, 2278-2289.

Hanashima, C., Li, S. C., Shen, L., Lai, E., and Fishell, G. (2004). Foxg1 suppresses early cortical cell fate. Science *303*, 56-59.

Hansford, L. M., Smith, S. A., Haber, M., Norris, M. D., Cheung, B., and Marshall, G. M. (2003). Cloning and characterization of the human neural cell adhesion molecule, CNTN4 (alias BIG-2). Cytogenet. Genome Res. *101*, 17-23.

Hantaz-Ambroise, D., Vigny, M., and Koenig, J. (1987). Heparan sulfate proteoglycan and laminin mediate two different types of neurite outgrowth. J. Neurosci. *7*, 2293-2304.

Hashimoto, K., and Ishima, T. (2010). A novel target of action of minocycline in NGF-induced neurite outgrowth in PC12 cells: translation inititation factor eIF4AI. PLoS One *5*, e15430.

Hebert, B., Pietropaolo, S., Même, S., Laudier, B., Laugeray, A., Doisne, N., Quartier, A., Lefeuvre, S., Got, L., Cahard, D., et al. (2014). Rescue of fragile X syndrome phenotypes in Fmr1 KO mice by the small-molecule PAK inhibitor FRAX486. Orphanet. J., Rare Dis. *9*, 124.

Hehr, U., Uyanik, G., Gross, C., Walter, M. C., Bohring, A., Cohen, M., Oehl-Jaschkowitz, B., Bird, L. M., Shamdeen, G. M., Bogdahn, U., et al. (2007). Novel POMGnT1 mutations define broader phenotypic spectrum of muscle-eye-brain disease. Neurogenetics *8*, 279-288.

Heldring, N., Joseph, B., Hermanson, O., and Kioussi, C. (2012). Pitx2 expression promotes p21 expression and cell cycle exit in neural stem cells. CNS Neurol. Disord. Drug Targets *11*, 884-892.

Helsmoortel, C., Vulto-van Silfhout, A. T., Coe, B. P., Vandeweyer, G., Rooms, L., van den Ende, J., Schuurs-Hoeijmakers, J. H.,Marcelis, C. L., Willemsen, M. H., Vissers, L. E., et al. (2014). A SWI/SNF-related autism syndrome caused by de novo mutations in ADNP. Nat. Genet. *46*, 380-384.

Heng, Y. H., McLeay, R. C., Harvey, T. J., Smith, A. G., Barry, G., Cato, K. Plachez, C., Little, E., Mason, S., Dixon, C., et al. (2014). NFIX regulates neural progenitor cell differentiation during hippocampal morphogenesis. Cereb. Cortex *24*, 261-279.

Henson, M. A., Roberts, A. C., Pérez-Otaño, I., and Philpot, B. D. (2010). Influence of the NR3A subunit on NMDA receptor functions. Prog. Neurobiol. *91*, 23-37.

Hirotsune, S., Fleck, M. W., Gambello, M. J., Bix, G. J., Chen, A., Clark, G. D., Ledbetter, D. H., McBain, C. J., and Wynshaw-Boris, A. (1998). Graded reduction of Pafah1b1 (Lis1) activity results in neuronal migration defects and early embryonic lethality. Nat. Genet. *19*, 333-339.

Honegger, P., and Lenoir, D. (1980). Triodothyronine enhancement of neuronal differentiation in aggregating fetal rat brain cells cultured in a chemically defined medium. Brain Res. *199*, 425-434.

Hoogenraad, C. C., Milstein, A. D., Ethell, I. M., Henkemeyer, M., and Sheng, M. (2005). GRIP1 controls dendrite morphogenesis by regulating EphB receptor trafficking. Nat. Neurosci. *8*, 906-915.

Horsfield, J. A., Anagnostou, S. H., Hu, J. K., Cho, K. H., Geisler, R., Lieschke, G., Crosier, K. E., and Crosier, P. S. (2007). Cohesin-dependent regulation of Runx genes. Development *134*, 2639-2649.

Hörster, F., Schwab, M. A., Sauer, S. W., Pietz, J., Hoffman, G. F., Okun, J. G., Kölker, S., and Kins, S. (2006). Phenylalanine reduces synaptic density in mixed cortical cultures from mice. Pediatr. Res. *59*, 544-548.

Huang, T. N., Chuang, H. C., Chou, W. H., Chen, C. Y., Wang, H. F., Chou, S. J., and Hsueh, Y. P. (2014). Tbr1 haploinsufficiency impairs amygdalar axonal projections and results in cognitive abnormality. Nat. Neurosci. *17*, 240-247.

Huber, O., and Weiske, J. (2008). Beta-catenin takes a HIT. Cell Cycle *7*, 1326-1331.

Israsena, N., Hu, M., Fu, W., Kan, L., and Kessler, J. A. (2004). The presence of FGF2 signaling determines whether beta-catenin exerts effects on proliferation or neuronal differentiation of neural stem cells. Dev Biol. *268*, 220-231.

Ito, T., Williams-Nate, Y., Iwai, M., Tsuboi, Y., Hagiyama, M., Ito, A., Sakurai-Yageta, M., and Murakami, Y. (2011). Transcriptional regulation of the CADM1 gene by retinoic acid during the neural differentiation of murine embryonal carcinoma P19 cells. Genes Cells *16*, 791-802.

Jafarzadeh, N., Javeri, A., Khaleghi, M., and Taha, M. F. (2014). Oxytocin improves proliferation and neural differentiation of adipose tissue-derived stem cells. Neurosci. Lett. *564*, 105-110.

Jauregui, A. R., Nguyen, K. C., Hall, D. H. and Barr, M. M. (2008). The Caenorhabditis elegans nephrocystins act as global modifiers of cilium structure. J. Cell Biol. *180*, 973-988.

Jin, K., Wang, X., Xie, L., Mao, X. O., and Greenberg, D. A. (2010). Transgenic ablataion of doublecortin-expressing cells suppresses adult neurogenesis and worsens stroke outcome in mice. Proc. Natl. Acad. Sci. USA *107*, 7993-7998.

Jolly, L. A., Homan, C. C., Jacob, R., Barry, S., and Gecz, J. (2013). The *UPF3B* gene, implicated in intellectual disability, autism, ADHD and childhood onset schizophrenia regulates neural progenitor cell behaviour and neuronal outgrowth. Hum. Mol. Genet. *22*, 4673-4687.

Joo, J. Y., Kim, B. W., Lee, J. S., Park, J. Y., Kim, S., Yun, Y. J., Lee, S. H., Lee, S. H., Rhim, H., and Son, H. (2007). Activation of NDMA receptors increases proliferation and differentiation of hippocampal neural progenitor cells. J. Cell Sci. *120*, 1358-1370.

Jori, F. P., Napolitano, M. A., Melone, M. A., Cipollaro, M., Cascino, A., Altucci, L., Peluso, G., Giordano, A., and Galderisi, U. (2005). Molecular pathways involved in neural in vitro differentiation of marrow stromal stem cells. J. Cell. Biochem. *94*, 645-655.

Judas, M., Sedmak, G., Rados, M., Sarnavka, V., Fumić, K., Willer, T., Gross, C., Hehr, U., Strahl, S., Cuk, M., et al. (2009). POMT1-associated Walker-Warburg syndrome: a disorder of dendritic development of neocortical neurons. Neuropediatrics *40*, 6-14.

Kametaka, S., Kametaka, A., Yonekura, S., Haruta, M., Takenoshita, S., Goto, S., and Waguri, S. (2012). AP-1 clathrin adaptor and CG8538/Aftiphilin are involved in Notch signaling during eye development in Drosophila melanogaster. J. Cell. Sci. *125*, 634-648.

Kamiya, A., Tomoda, T., Chang, J., Takaki, M., Zhan, C., Morita, M., Cascio, M. B., Elashvili, S., Koizum, H., Takanezawa, Y., et al. (2006). DISC1-NDEL1/NUDEL protein interaction, an essential component for neurite outgrowth, is modulated by genetic variations of DISC1. Hum. Mol. Genet. *15*, 3313-3323.

Kato, M., Yoshimura, S., Kohuzawa, J., Kitajima, H., Kaku, Y., Iwama, T., Shinoda, J., Kunisada, T., and Sakai, N. (2004). Hepatocyte growth factor promotes neuronal differentiation of neural stem cells derived from embryonic stem cells. Neuroreport *15*, 5-8.

Kay, J. N., Chu, M. W., and Sanes, J. R. (2012). MEGF10 and MEGF11 mediate homotypic interactions required for mosaic spacing of retinal neurons. Nature *483*, 465-469.

Kerin, T., Ramanathan, A., Rivas, K., Grepo, N., Coetzee, G. A., and Campbell, D. B. (2012). A noncoding RNA antisense to moesin at 5p14.1 in autism. Sci. Transl. Med. *4*, 128ra40.

Kim, E., and Hoffman, D. A. (2012). Dynamic regulation of synaptic maturation state by voltage-gated A-type K+ channels in CA1 hippocampal pyramidal neurons. J. Neurosci. *32*, 14427-14432.

Kim, J. H., Kim, J. H., Yang, E., Park, J. H., Yu, Y. S., and Kim, K. W. (2009). Shank2 expression coincides with neuronal differentiation in the developing retina. Exp. Mol. Med. *41*, 236-242.

Kim, H. M., Qu, T., Kriho, V., Lacor, P., Smalheiser, N., Pappas, G. D., Guidotti, A., Costa, E., and Sugaya, K. (2002). Reelin function in neural stem cell biology. Proc. Natl. Acad. Sci. USA *99*, 4020-4025.

Kim, J. C., Ou, Y. Y., Badano, J. L., Esmail, M. A., Leitch, C. C., Fiedrich, E., Beales, P. L., Archibald, J. M., Katsanis, N., Rattner, J. B., et al. (2005). MKKS/BBS6, a divergent chaperonin-like protein linked to the obesity disorder Bardet-Biedl syndrome, is a novel centrosomal component required for cytokinesis. J. Cell Sci. *118*, 1007-1020.

Koenekoop, R. K. (2003). Abnormal retinal architecture in a 33-week-old fetus with LCA and a homozygous C330Y mutation in RPE65. Ophthalmic Genet. *24*, 125-126.

Kramer, J. M., Kochinke, K., Oortveld, M. A., Marks, H., Kramer, D., de Jong, E. K., Asztalos, Z., Westwood, J. T., Stunnenberg, H. G., Sokolowski, M. B., et al. (2011). Epigenetic regulation of learning and memory by Drosophila EHMT/G9a. PLoS Biol. *9*, e1000569.

Krell, D., Assoku, M., Galloway, M., Mulholland, P., Tomlinson, I., and Bardella, C. (2011). Screen for IDH1, IDH2, IDH3, D2HGDH and L2HGDH mutations in glioblastoma. PLoS One *6*, e19868.

Kumar, R. A., KaraMohamed, S., Sudi, J., Conrad, D. F., Brune, C., Badner, J. A., Gilliam, T. C., Nowak, N. J., Cook, E. H., Jr., Dobyns, W. B., et al. (2008). Recurrent 16p11.2 microdeletions in autism. Hum. Mol. Genet. *17*, 628-638.

Kumar, R. A., Sudi, J., Babatz, T. D., Brune, C. W., Oswald, D., Yen, M., Nowak, N. J., Cook, E. H., Christian, S. L., and Dobyns, W. B. (2010). A de novo 1p34.2 microdeletion identifies the synaptic vesicle gene RIMS3 as a novel candidate for autism. J. Med. Genet. *47*, 81-90.

Kwon, O. B., Paredes, D., Gonzalez, C. M., Neddens, J., Hernandez, L., Vullhorst, D., and Buonanno, A. (2008). Neuregulin-1 regulates LTP at CA1 hippocampal synapses through activation of dopamine D4 receptors. Proc. Natl. Acad. Sci. USA *105*, 15587-15592.

Lachyankar, M. B., Sultana, N., Schonhoff, C. M., Mitra, P., Poluha, W., Lambert, S., Quesenberry, P. J., Litofsky, N. S., Recht, L. D., Nabi, R., et al. (2000). A role for nuclear PTEN in neuronal differentiation. J. Neurosci. *20*, 1404-1413.

Laifenfeld, D., Klein, E., and Ben-Schachar, D. (2002). Norepinephrine alters the expression of genes involved in neuronal sprouting and differentiation: relevance for major depression and antidepressant mechanisms. J. Neurochem. *83*, 1054-1064.

Layman, W. S., McEwen, D. P., Beyer, L. A., Lalani, S. R., Fernbach, S. D., Oh, E., Swaroop, A., Hegg, C. C., Raphael, Y., Martens, J. R., et al. (2009). Defects in neural stem cell proliferation and olfaction in Chd7 deficient mice indicate a mechanism for hyposmia in human CHARGE syndrome. Hum. Mol. Genet. *18*, 1909-1923.

Lee, M. T., Chen, C. H., Lee, C. S., Chen, C. C., Chong, M. Y., Ouyang, W. C., Chiu, N. Y., Chuo, L. J., Chen, C. Y., Tan, H. K., et al. (2011). Genome-wide association study of bipolar I disorder in the Han Chinese population. Mol. Psychiatry *16*, 548-556.

Lee, L. J., Lo, F. S., and Erzurumlu, R. S. (2005). NMDA receptor-dependent regulation of axonal and dendritic branching. J. Neurosci. *25*, 2304-2311.

Leivo, I., Jee, K. J., Heikinheimo, K., Laine, M., Ollila, J., Nagy, B., and Knuutila, S. (2005). Characterization of gene expression in major types of salivary gland carcinomas with epithelial differentiation. Cancer Genet. Cytogenet. *156*, 104-113.

Leonard, J. L., and Farwell, A. P. (1997). Thyroid hormone-regulated actin polymerization in brain. Thyroid *7*, 147-151.

Lepagnol-Bestel, A. M., Maussion, G., Boda, B., Cardona, A., Iwayama, Y., Delezoide, A. L., Moalic, J. M., Muller, D., Dean, B., Yoshikawa, T., et al. (2008). SLC25A12 expression is associated with neurite outgrowth and is upregulated in the prefrontal cortex of autistic subjects. Mol. Psychiatry *13*, 385-397.

Li, D., Gu, X., Lu, L., and Liang, L. (2010). Effects of phenylalanine on the survival and neurite outgrowth of rat cortical neurons in primary cultures: possible involvement of brain-derived neurotrophic factor. Mol. Cell Biochem. *339*, 1-7.

Li, C., Ito, H., Fujita, K., Shiwaku, H., Qi, Y., Tagawa, K., Tamura, T., and Okazawa, H. (2013). Sox2 transcriptionally regulates PQBP1, an intellectual disability-microcephaly causative gene, in neural stem progenitor cells. PLoS One *8*, e68627.

Li, J. M., Mogi, M., Tsukuda, K., Tomochika, H., Iwanami, J., Min, L. J., Nahmias, C., Iwai, and M., Horiuchi, M. (2007). Angiotensin II-induced neural differentiation via angiotensin II type 2 (AT2) receptor-MMS2 cascade involving interaction between AT2 receptor-interacting protein and Src homology 2 domain-containing protein-tyrosine phosphatase 1. Mol. Endocrinol. *21*, 499-511.

Li, H., Radford, J. C., Ragusa, M. J., Shea, K. L., McKercher, S. R., Zaremba J. D., Soussou, W., Nie, Z., Kang, Y. J., Nakanishi, N., et al. (2008). Transcription factor MEF2C influences neural stem/progenitor cell differentiation and maturation in vivo. Proc. Natl. Acad. Sci. USA *105*, 9397-9402.

Liss, L., and Grümer, H. D. (1966), Effect of L-phenylalanine on central nervous system elements in tissue culture. J. Neurol. Neurosurg. Psychiatry *29*, 371-374.

Lu, T. Chen, R., Cox, T. C., Moldrich, R. X., Kurniawan, N., Tan, G., Perry, J. K., Ashworth, A., Barlett, P. F., Xu, L., et al. (2013). X-linked microtubule-associated protein, Mid1, regulates axon development. Proc. Natl. Acad. Sci. USA *110*, 19131-19136.

Luo, Y., Shan, G., Gui, W., Smrt, R. D., Johnson, E. B., Li, X., Pfeiffer, R. L., Szulwach, K. E., Duan, R., Barkho, B. Z., et al. (2010). Fragile X mental retardation protein regulates proliferation and differentiation of adult neural stem/progenitor cells. PLoS Genet. *6*, e1000898.

Ma, D. Q., Rabionet, R., Konidari, I., Jaworski, J., Cukier, H. N., Wright, H. H., Abramson, R. K., Gilbert, J. R., Cuccaro, M. L., Pericak-Vance, M. A., et al. (2010). Association and gene-gene interaction of SLC6A4 and ITGB3 in autism. Am J. Med. Genet. B Neuropsychiatr. Genet. *153B*, 477-483.

Maddodi, N., Bhat, K. M., Devi, S., Zhang, S. C., and Setaluri, V. (2010). Oncogenic BRAFV600E induces expression of neuronal differentiation marker MAP2 in melanoma cells by promoter demethylation and down-regulation of transcription repressor HES1. J. Biol. Chem. *285*, 242-254.

Maestrini, E., Pagnamenta, A. T., Lamb, J. A., Bacchelli, E., Sykes, N. H., Sousa, I., Toma, C., Barnby, G., Butler, H., Winchester, L., et al. (2010). High-density SNP association study and copy number variation analysis of the AUTS1 and AUTS5 loci implicate the IMMP2L-DOCK4 gene region in autism susceptibility. Mol. Psychiatry *15*, 954-968.

Magome, T., Hattori, T., Taniguchi, M., Ishikawa, T., Miyata, S., Yamada, K., Takamura, H., Matsuzaki, Ito, A., Tohyama, M., et al. (2013). XLMR protein related to neurite extension (Xpn/KIAA2022) regulates cell-cell and cell-matrix adhesion and migration. Neurochem. Int. *63*, 561-569.

Mah, W., Ko, J., Nam, J., Han, K., Chung, W. S., and Kim, E. (2010). Selected SALM (synaptic adhesion-like molecule) family proteins regulate synapse formation. J. Neurosci. *30*, 5559-5568.

Maina, F., Hilton, M. C., Ponzetto, C., Davies, A. M., and Klein, R. (1997). Met receptor signaling is required for sensory nerve development and HGF promotes axonal growth and survival of sensory neurons. Genes Dev. *11*, 3341-3350.

Mao, Y., Ge, X, Frank, C. L., Madison, J. M., Koehler, A. N., Doud, M. K., Tassa, C., Berry, E. M., Soda, T., Singh, K. K., et al. (2009). Disrupted in schizophrenia 1 regulates neuronal progenitor proliferation via modulation of GSK3beta/beta-catenin signaling. Cell *136*, 1017-1031.

Mardirossian, S., Rampon, C., Salvert, D., Fort, P., and Sarda, N. (2009). Impaired hippocampal plasticity and altered neurogenesis in adult *Ube3a* maternal deficient mouse model for Angelman syndrome. Exp. Neurol. *220*, 341-348.

Maric, D., Fiorio Pla, A., Chang, Y. H., and Barker, J. L. (2007). Self-renewing and differentiating properties of cortical neural stem cells are selectively regulated by basic

fibroblast growth factor (FGF) signaling via specific FGF receptors. J. Neurosci. *27*, 1836-1852.

Maric, D., Liu, Q. Y., Grant, G. M., Andreadis, J. D., Hu, Q., Chang, Y. H., Barker, J. L., Joseph, J., Stenger, D. A., and Ma, W. (2000). Functional ionotropic glutamate receptors emerge during terminal cell division and early neuronal differentiation of rat neuroepithelial cells. J. Neurosci. Res. *61*, 652-662.

Marteyn, A., Maury, Y., Gauthier, M. M., Lecuyer, C., Vernet, R., Denis, J. A., Pietu, G., Peschanski, M., and Martinat, C. (2011). Mutant human embryonic stem cells reveal neurite and synapse formation defects in type 1 myotonic dystrophy. Cell Stem Cell *8*, 434-444.

Martinez-Ceballos, E., and Gudas, L. J. (2008). Hoxa1 is required for the retinoice acid-induced differentiation of embryonic stem cells into neurons. J. Neurosci. Res. *86*, 2809-2819.

Martinez de Lagran, M., Benavides-Piccione, R., Ballesteros-Yañez, I., Calvo, M., Morales, M., Fillat, C., Defelipe, J., Ramakers, G. J., and Dierssen, M. (2012). Dyrk1A influences neuronal morphogenesis through regulation of cytoskeletal dynamics in mammalian cortical neurons. Cereb. Cortex *22*, 2867-2877.

Maul, B., von Bohlen und Halbach, O., Becker, A., Sterner-Kock, A., Voigt, J. P., Siems, W. E., Grecksch, G., and Walther, T. (2008). Impaired spatial memory and altered dendritic spine morphology in angiotensin II receptor-deficient mice. J. Mol. Med. (Berl). *86*, 563-571.

McNeill, B., Mazerolle, C., Bassett, E. A., Mears, A. J., Ringuette, R., Lagali, P., Picketts, D. J., Paes, K., Rice, D., and Wallace, V. A. (2013). Hedgehog regulates Norrie disease protein to drive neural progenitor self-renewal. Hum. Mol. Genet. *22*, 1005-1016.

Meikle, L., Talos, D. M., Onda, H., Pollizzi, K., Rotenberg, A., Sahin, M., Jensen, F. E., and Kwiatkowski, D. J. (2007). A mouse model of tuberous sclerosis: neuronal loss of Tsc1 causes dysplastic and ectopic neurons, reduced myelination, seizure activity, and limited survival. J. Neurosci. *27*, 5546-5558.

Melicharek, D. J., Ramirez, L. C., Singh, S., Thompson, R., and Marenda, D. R. (2010). Kismet/CHD7 regulates axon morphology, memory and locomotion in Drosphila model of CHARGE syndrome. Hum. Mol. Genet. *19*, 4253-4264.

Melke, J., Goubrain Botros, H., Chaste, P., Betancur, C., Nygren, G., Anckarsäter, H., Rastam, M., Ståhlberg, O., Gilberg, I. C., Delorme, R., et al. (2008). Abnormal melatonin synthesis in autism spectrum disorders. Mol. Psychiatry *13*, 90-98.

Meloni, I., Parri, V., De Filippis, R., Ariani, F., Artuso, R., Bruttini, M., Katzaki, E., Longo, I., Mari, F., Bellan, C., et al. (2009). The XLMR gene ACSL4 plays a role in dendritic spine architecture. Neuroscience *159*, 657-669.

Mercati, O., Danckaert, A., André-Leroux, G., Bellinzoni, M., Gouder, L., Watanade, K., Shimoda, Y., Grailhe, R., De Chaumont, F., Bourgeron, T., et al. (2013). Contaction 4, -5 and -6 differentially regulate neuritogenesis while they display identical PTPRG binding sites. Biol. Open *2*, 324-334.

Métrailler, S., Emery, M., Schorderet, D. F., Cottet, S., and Roduit, R. (2013). ERK1/2 pathway is activated in degenerated Rpe65-deficient mice. Exp. Eye Res. *116*, 86-95.

Mikulak, J., Negrini, S., Klajn, A., D'Alessandro, R., Mavilio, D., and Meldolesi, J. (2012). Dual REST-dependence of L1CAM: from gene expression to alternative splicing governed by Nova2 in neural cells. J. Neurochem. *120*, 699-709.

Mills, P. B., Footitt, E. J., Mills, K. A., Tuschl, K., Aylett, S., Varadkar, S., Hemingway, C., Marlow, N., Rennie, J., Baxter, P., et al. (2010). Genotypic and phenotypic spectrum of pyridoxine-dependent epilepsy (ALDH7A1 deficiency). Brain *133*, 2148-2159.

Mishra, A., Godavarthi, S. K., and Jana, N. R. (2009). *UBE3A*/E6-AP regulates cell proliferation by promoting proteasomal degradation of p27. Neurobiol. Dis. *36*, 26-34.

Mitsui, S., Hidaka, C., Furihata, M., Osako, Y., and Yuri, K. (2013). A mental retardation gene, motopsin/prss12, modulates cell morphology by interaction with seizure-related gene 6. Biochem. Biophys. Res. Commun. *436*, 638-644.

Mohapel, P., Leanza, G., Kokaia, M., and Lindvall, O. (2005). Forebrain acetylcholine regulates adult hippocampal neurogenesis and learning. Neurobiol. Aging *26*, 939-946.

Montpetit, A., Côté, S., Brustein, E., Drouin, C. A., Lapointe, L., Boudreau, M., Meloche, C., Drouin, R., Hudson, T. J., Drapeau, P., et al. (2008). Disruption of AP1S1, causing a novel neurocutaneous syndrome, perturbs development of the skin and spinal cord. PLoS Genet. *4*, e10000296.

Moosmang, S., Haider, N., Klugbauer, N., Adelsberger, H., Langweiser, N., Müller, J., Stiess, M., Marais, E., Schulla, V., Lacinova, L., et al. (2005). Role of hippocampal Cav1.2 Ca2+ channels in NDMA receptor-independent synaptic plasticity and spatial memory. J. Neurosci. *25*, 9883-9892.

Moreb, J. S. (2008). Aldehyde dehydrogenase as a marker for stem cells. Curr. Stem Cell Res. T. *3*, 237-246.

Moro, F., Pisano, T., Bernardina, B. D., Polli, R., Murgia, A., Zoccante, L., Darra, F., Battaglia, A.,Pramparo, T., Zuffardi, O., et al. (2006). Periventricular heterotopia in fragile X syndrome. Neurology *67*, 713-715.

Moroni, R. F., De Biasi, S., Colapietro, P., Larizza, L., and Beghini, A. (2006). Distinct expression pattern of microtubule-associated protein/microtubule affinity-regulating kinase 4 in differentiated neurons. Neuroscience *143*, 83-94.

Motazacker, M. M., Rost, B. R., Hucho, T., Garshasbi, M., Kahrizi, K., Ullman, R., Abedini, S. S., Nieh, S. E., Amini, S. H., Goswami, C., et al. (2007). A defect in the ionotropic glutamate receptor 6 gene (GRIK2) is associated with autosomal recessive mental retardation. Am. J. Hum. Genet. *81*, 792-798.

Mnayer, L., Khuri, S., Merheby, H. A, Meroni, G., and Elsas, L. J. (2006). A structure-function study of MID1 mutations associated with a mild Opitz phenotype. Mol. Genet. Metab. *8*, 198-203.

Najm, J., Horn, D., Wimplinger, I., Golden, J. A., Chizhikov, V. V., Sudi, J., Christian, S. L., Ullmann, R., Kuechler, A., Haas, C. A., et al. (2008). Mutations of CASK cause an X-linked brain malformation phenotype with microcephaly and hypoplasia of the brainstem and cerebellum. Nat. Genet. *40*, 1065-1067.

Napoli, I., Mercaldo, V., Boyl, P. P., Eleuteri, B., Zalfa, F., De Rubeis, S., Di Marino, D., Mohr, E., Massimi, M., Falconi, M., et al. (2008). The fragile X syndrome protein represses activity-dependent translation through CYFIP1, a new 4E-BP. Cell *134*, 1042-1054.

NCBI. (2014). PRKCB protein kinase C, beta [*Homo sapiens* (human)]. Accessed on 8/28/2014 from http://www.ncbi.nlm.nih.gov/gene/5579

Ng, A., Uribe, R. A., Yieh, L., Nuckels, R., and Gross, J. M. (2009). Zebrafish mutations in gart and paics identify crucial roles for de novo purine synthesis in vertebrate pigmentation and ocular development. Development *136*, 2601-2611.

Nichols, A. J., and Olson, E. C. (2010). Reelin promotes neuronal orientation and dendritogenesis during preplate splitting. Cereb. Cortex *20*, 2213-2223.

Nie, D., Di Nardo, A., Han, J. M., Baharanyi, H., Kramvis, I., Huynh, T., Dabora, S., Codeluppi, S., Pandolfi, P. P., Pasquale, E. B., et al. (2010). Nat. Neurosci. *13*, 163-172.

Nishi, T., Lee, P. S., Oka, K., Levin, V. A., Tanase, S., Morino, Y., and Saya, H. (1991). Differential expression of two types of the neurofibromatosis type 1 (NF1) gene transcripts related to neuronal differentiation. Oncogene *6*, 1555-1559.

Nishiyama, M., Skoultchi, A. I., and Nakayama, K. I. (2012). Histone H1 recruitment by CHD8 is essential for suppression of the Wnt—β-catenin signaling pathway. Mol. Cell. Biol. *32*, 501-512.

Nodé-Langlois, R., Muller, D., and Boda, B. (2006). Sequential implication of the mental retardation proteins ARHGEF6 and PAK3 in spine morphogenesis. J. Cell Sci. *119*, 4986-4993.

Nurcombe, V., Ford, M. D., Wildschut, J. A., and Barlett, P. F. (1993). Developmental regulation of neural responses to FGF-1 and FGF-2 by heparin sulfate proteoglycan. Science *260*, 103-106.

Nussbaum, J., Xu, Q., Payne, T. J., Ma, J. Z., Huang, W., Gelernter, J., and Li, M. D. (2008). Significant association of the neurexin-1 gene (NRXN1) with nicotine dependence in European- and African-American smokers. Hum. Mol. Genet. *17*, 1569-1577.

Okamura, Y., and Shidara, M. (1990). Changes in sodium channels during neural differentiation in the isolated blastomere of the ascidian embryo. J. Physiol. *431*, 39-74.

Oksenberg, N., Stevison, L., Wall, J. D., and Ahituv, N. (2013). Function and regulation of AUTS2, a gene implicated in autism and human evolution. PLoS Genet. *9*, e1003221.

Olbrich, H., Häffner, K., Kispert, A., Völkel, A., Volz, A., Sasmaz, G., Reinhardt, R., Hennig, S., Lehrach, H., Konietzko, N., et al. (2002). Mutations in DNAH5 cause primary ciliary dyskinesia and randomization of left-right asymmetry. Nat. Genet. *30*, 143-144.

Otero, J. J., Fu, W., Kan, L., Cuadra, A. E., and Kessler, J. A. (2004). Beta-catenin signaling is required for neural differentiation of embryonic stem cells. Development *131*, 3545-3557.

Ouyang, Q., Lizarraga, S. B., Schmidt, M., Yang, U., Gong, J., Ellisor, D., Kauer, J. A., and Morrow, E. M. (2013). Christianson syndrome protein NHE6 modulates TrkB endosomal signaling required for neuronal circuit development. Neuron *80*, 97-112.

Pagani, J. H., Zhao, M., Cui, Z., Williams Avram, S. K., Caruana, D. A., Dudek, S. M., and Young, W. S. (2014). Role of the vasopressin 1b receptor in rodent aggressive behavior and synaptic plasticity in hippocampal area CA2. Mol. Psychiatry, doi: 10.1038/mp.2014.47.

Page, D. T., Kuti, O. J., Prestia, C., and Sur, M. (2009). Haploinsufficiency for Pten and serotonin transporter cooperatively influences brain size and social behavior. Proc. Natl. Acad. Sci. USA *106*, 1989-1994.

Paglini, G., Kunda, P., Quiroga, S., Kosik, K., and Cáceres, A. (1998). Suppression of radixin and moesin alters growth cone morphology, motility, and process formation in primary cultured neurons. J. Cell Biol. *143*, 443-455.

Palmesino, E., Rousso, D. L., Kao, T. J., Klar, A., Laufer, E., Uemura, O., Okamoto, H., Novitch, B. G., and Kania, A. (2010). Foxp1 and Ihx1 coordinate motor neuron migration with axon trajectory choice by gating Reeling signaling. PLoS Biol. *8*, e1000446.

Pang, L., Sawada, T., Decker, S. J., and Saltiel, A. R. (1995). Inhibition of MAP kinase kinase blocks the differentiation of PC-12 cells induced by nerve growth factor. J. Biol. Chem. *270*, 13585-13588.

Paonessa, F., Latifi, S., Scarongella, H., Cesca, F., and Benfenati, F. (2013). Specificity protein 1 (Sp1)-dependent activation of the synapsin I gene (SYN1) is modulated by RE1-silencing transcription factor (REST) and 5'-cytosine-phosphoguanine (CpG) methylation. J. Biol. Chem. *288*, 3227-3239.

Paquin, A., Hordo, C., Kaplan, D. R., and Miller, F. D. (2009). Costello syndrome H-Ras alleles regulate cortical development. Dev. Biol. *330*, 440-451.

Park, T. S., and Zambidis, E. T. (2009). A role for the renin-angiotensin system in hematopoiesis. Haematologica *94*, 745-747.

Paşca, S. P., Portmann, T., Voineagu, I., Yazawa, M., Shcheglovitov, A., Paşca, A. M., Cord, B., Palmer, T. D., Chikahisa, S., Nishino, S., et al. (2011). Using iPSC-derived neurons to uncover cellular phenotypes associated with Timothy syndrome. Nat. Med. *17*, 1657-1662.

Pascual, M., and Guerri, C. (2007). The peptide NAP promotes neuronal growth and differentiation through extracellular signal-regulated protein kinase and Akt pathways, and protects neurons co-cultured with astrocytes damaged by ethanol. J. Neurochem. *103*, 557-568.

Pasteris, N. G., and Gorski, J. L. (1999). Isolation, characterization, and mapping of the mouse and human Fgd2 genes, faciogenital dysplasia (FGD1; Aarskog syndrome) gene homologues. Genomics *60*, 57-66.

Patrakitkomjom, S., Kobayashi, D., Morikawa, T., Wilson, M. M., Tsubota, N., Irie, A., Ozawa, T., Aoki, M., Arimura, N., Kaibuchi, K., et al. (2008). Neurofibromatosis type 1 (NF1) tumor suppressor, neurofibromin, regulates the neuronal differentiation of PC12 cells via its associating protein, CRMP-2. J. Biol. Chem. *283*, 9399-9413.

Pawlisz, A. S., Mutch, C., Wynshaw-Boris, A., Chenn, A., Walsh, C. A., and Feng, Y. (2008). Lis1-Nde1-dependent neuronal fate control determines cerebral cortical size and lamination. Hum. Mol. Genet. *17*, 2441-2455.

Peddada, S., Yasui, D. H., and LaSalle, J. M. (2006). Inhibitors of differentiation (ID1, ID2, ID3 and ID4) genes are neuronal targets of MeCP2 that are elevated in Rett syndrome. Hum. Mol. Genet. *15*, 2003-2014.

Peñagarikano, O., Abrahams, B. S., Herman, E. I., Winden, K. D., Gdalyahu, A., Dong, H., Sonnenblick, L. I., Gruver, R., Almajano, J., Bragin, A., et al. (2011). Absence of CNTNAP2 leads to epilepsy, neuronal migration abnormalities, and core autism-related deficits. Cell *147*, 235-246.

Penzes, P., Woolfrey, K. M., and Srivastava, D. P. (2011). Epac2-mediated dendritic spine remodeling: implications for disease. Mol. Cell. Neurosci. *46*, 368-380.

Pfeiffer, V., Götz, R., Xiang, C., Camarero, G., Braun, A., Zhang, Y., Blum, R., Heinsen, H., Nieswandt, B., and Rapp, U. R. (2013). Ablation of BRaf impairs neuronal differentiation in the postnatal hippocampus and cerebellum. PLoS One *8*, e58259.

Pietri, T., Easley-Neal, C., Wilson, C., and Washbourne, P. (2008). Six cadm/SynCAM genes are expressed in the nervous system of developing zebrafish. Dev. Dyn. *237*, 233-246.

Pincus, D. W., Dicicco-Bloom, E. M., and Black, I. B. (1990). Vasoactive intestinal peptide regulates mitosis, differentiation and survival of cultured sympathetic neuroblasts. Nature *343*, 564-567.

Pindolia, K., Chen, J., Cardwell, C., Cui, X., Chopp, M., and Wolf, B. (2012). Neurological deficits in mice with profound biotinidase deficiency are associated with demyelination and axonal degeneration. Neurobiol. Dis. *47*, 428-435.

Pistocchi, A., Fazio, G., Cereda, A., Ferrari, L., Bettini, L. R., Messina, G., Cotelli, F., Biondi, A., Selicorni, A., and Massa, V. (2013). Cornelia de Lange syndrome: NIPBL haploinsufficiency downregulates canonical Wnt pathway in zebrafish embryos and patients' fibroblasts. Cell Death Dis. *4*, e866.

Poeta, L., Fusco, F., Drongitis, D., Shoubridge, C., Manganelli, G., Filosa, S., Paciolla, M., Courtney, M., Collombat, P., Lioi, M. B., et al. (2013). A regulatory path associated with X-linked intellectual disability and epilepsy links KDM5C to the polyalanine expansions in ARX. Am. J. Hum. Genet. *92*, 114-125.

Poirier, K., Keays, D. A., Francis, F., Saillour, Y., Bahi, N., Manouvrier, S., Fallet-Bianco, C., Pasquier, L., Toutain, A., Tuy, F. P., et al. (2007). Large spectrum of lissencephaly and pachygyria phenotypes resulting from de novo missense mutations in tubulin alpha 1A (TUBA1A). Hum. Mutat. *28*, 1055-1064.

Polleux, F., Ince-Dunn, G., and Ghosh, A. (2007). Transcriptional regulation of vertebrate axon guidance and synapse formation. Nat. Rev. Neurosci. *8*, 331-340.

Qi, J., Chi, L., Labeit, S., and Banes, A. J. (2008). Am. J. Physiol. Cell. Physiol. *295*, C975-985.

Qiu, M. S., and Green, S. H. (1992). PC12 cell neuronal differentiation is associated with prolonged p21ras activity and consequent prolonged ERK activity. Neuron *9*, 705-717.

Qiu, J., Shi, G., Jia, Y., Li, J., Wu, M., Li, J., Dong, S., and Wong, J. (2010). The X-linked mental retardation gene PHF8 is a histone demethylase involved in neuronal differentiation. Cell Res. *20*, 908-918.

Qureshi, K., Rao, K. V., and Qureshi, I. A. (1998). Differential inhibition by hyperammonemia of the electron transport chain enzymes in synaptosomes and non-synaptic mitochondria in ornithine transcarbamylase-deficient spf-mice: restoration by acetyl-L-carnitine. Neurochem. Res. *23*, 855-861.

Raabe, E. H., Lim, K. S., Kim, J. M., Meeker, A., Mao, X. G., Nikkhah, G., Maciaczyk, J., Kahlert, U., Jain, D., Bar, E., et al. (2011). BRAF activation induces transformation and then senescence in human neural stem cells: a pilocytic astrocytoma model. Clin. Cancer Res. *17*, 3590-3599.

Raft, S., Nowotschin, S., Liao, J., and Morrow, B. E. (2004). Suppression of neural fate and control of inner ear morphogenesis by Tbx1. Development *131*, 1801-1812.

Ramirez, I. B., Pietka, G., Jones, D. R., Divecha, N., Alia, A., Baraban, S. C., Hurlstone, A. F., and Lowe, M. (2012). Impaired neural development in a zebrafish model for Lowe syndrome. Hum. Mol. Genet. *21*, 1744-1759.

Reid, C. A., Leaw, B., Richards, K. L., Richardson, R., Wimmer, V., Yu, C., Hill-Yardin, E. L., Lerche, H., Scheffer, I. E., et al. (2014). Reduced dendritic arborization and hyperexcitability of pyramidal neurons in a Scn1b-based model of Dravet syndrome. Brain *137*, 1701-1715.

Richter, C., and Kass, G. E. (1991). Oxidative stress in mitochondria: its relationship to cellular Ca2+ homeostasis, cell death, proliferation, and differentiation. Chem. Bio. Interact. *77*, 1-23.

Rocas, D., Alix, E., Michel, J., Cordier, M. P., Labalme, A., Guilbert, H., Till, M., Schluth-Bolard, C., de Haas, P., Massardiet, J., Portes, V. D., et al. (2013). Neuropathological features in a female fetus with OPHN1 deletion and cerebellar hypoplasia. Eur. J. Med. Genet. *56*, 270-273.

Rolland, S. G., and Conradt, B. (2010). New role of the BCL2 family of proteins in the regulation of mitochondrial dynamics. Curr. Opin. Biol. *852*, 852-858.

Rooslid, T., Castronovo, S., Villoso, A., Ziemba, A., and Pizzorno, G. (2011). A novel structural mechanism for redox regulation of uridine phosphorylase 2 activity. J. Struct. Biol. *176*, 229-237.

Rosário, M., Franke, R., Bednarski, C., and Birchmeier, W. (2007). The neurite outgrowth multiadaptor RhoGAP, NOMA-GAP, regulates neurite extension through SHP2 and Cdc42. J. Cell Biol. *178*, 503-516.

Rosenfelder, L. M. (2009). Effects of silencing the mental retardation gene Jarid1c on neuronal differentiation of pluripotent P19 cells. PhD diss., Worcester Polytechnic Institute.

Rouaux, C., and Arlotta, P. (2010). Fezf2 directs the differentiation of corticofugal neurons from striatal progenitors in vivo. Nat. Neurosci. *13*, 1345-1347.

Rousso, D. L., Pearson, C. A., Gaber, Z. B., Miquelajauregui, A., Li, S., Portera-Cailliau, C., Morrisey, E. E., and Novitch, B. G. (2012). Foxp-mediated suppression of N-cadherin regulates neuroepithelial character and progenitor maintenance in the CNS. Neuron *74*, 314-330.

Rowe, S. J., Messenger, N. J., and Warner, A. E. (1993). The role of noradrenaline in the differentiation of amphibian embryonic neurons. Development *119*, 1343-1357.

Sakai, T., Furuyama, T., Ohoka, Y., Miyazaki, N., Fujioka, S., Sugimoto, H., Amasaki, M., Hattori, S., and Matsuya, T. (1999). Mouse semaphoring H induces PC12 cell neurite outgrowth activating Ras-mitogen-activated protein kinase signaling pathway via Ca(2+) influx. J. Biol. Chem. *274*, 29666-29671.

Sala, M., Braida, D., Lentini, D., Busnelli, M., Bulgheroni, E., Capurro, V., Finardi, A., Donzelli, A., Pattini, L., Rubino, T., et al. (2011). Biol. Psychiatry *69*, 875-882.

Salim, K., Kehoe, L., Minkoff, M. S., Bilsland, J. G., Munoz-Sanjuan, I., and Guest, P. C. (2006). Identification of differentiating neural progenitor cell markers using shotgun isobaric tagging mass spectrometry. Stem Cells Dev. *15*, 461-470.

Sarnat, H. B., Benjamin, D. R., Siebert, J. R., Kletter, G. B., and Cheyette, S. R. (2002). Agenesis of the mesencephalon and metencephalon with cerebellar hypoplasia: putative mutation in the EN2 gene—report of 2 cases in early infancy. Pediatr. Dev. Pathol. *5*, 54-68.

Satin, C., and Kitajima, K. (2013). Impact of structural aberrancy of polysialic acid and its synthetic enzyme ST8SIA2 in schizophrenia. Front. Cell Neurosci. *7*, 61.

Sautin, Y. Y., Nakagawa, T., Zharikov, S., and Johnson, R. J. (2007). Adverse effects of the classic antioxidant uric acid in adipocytes: NADPH oxidase-mediated oxidative/nitrosative stress. Am. J. Physiol. Cell Physiol. *293*, C584-596.

Savarese, F., Dávila, A., Nechanitzky, R., De La Rossa-Velazquez, I., Pereira, C. F., Engelka, R., Takahashi, K., Jenuwein, T., Kohwi-Shigematsu, T., Fisher, A. G., et al.

(2009). Satb1 and Satb2 regulate embryonic stem cell differentiation and Nanog expression. Genes Dev. *23*, 2625-2638.

Schaarschmidt, G., Wegner, F., Schwarz, S. C., Schmidt, H., and Schwarz, J. (2009). Characterization of voltage-gated potassium channels in human neural progenitor cells. PLoS One *4*, e6168.

Schäfer, M. K., and Frotscher, M. (2012). Role of L1CAM for axon sprouting and branching. Cell Tissue Res. *349*, 39-48.

Schlosser, G., Koyano-Nakagawa, N., and Kintner, C. (2002). Thyroid hormone promotes neurogenesis in the *Xenopus* spinal cord. Dev. Dyn. *225*, 485-498.

Schmeisser, M. J., Baumann, B., Johannsen, S., Vindedal, G. F., Jensen, V., Hvalby, Ø. C., Sprengel, R., Seither, J., Magbool, A., Magnutzki, A., et al. (2012). IκB kinase/nuclear factor κB-dependent insulin-like growth factor 2 (Igf2) expression regulates synapse formation and spine maturation via Igf2 receptor signaling. J. Neurosci. *32*, 5688-5703.

Schneider, R. A., Hu, D., Rubenstein, J. L. R., Maden, M., and Helms, J. A. (2001). Local retinoid signaling coordinates forebrain and facial morphogenesis by maintaining FGF8 and SHH. Development *128*, 2755-2767.

Schuetz, G., Rosário, M., Grimm, J., Boeckers, T. M., Gundelfinger, E. D., and Birchmeier, W. (2004). The neuronal scaffold protein Shank3 mediates signaling and biological function of the receptor tyrosine kinase Ret in epithelial cells. J. Cell. Biol. *167*, 945-952.

Schüller, A. C., Ahmed, Z., and Ladbury, J. E. (2008). Extracellular point mutations in FGFR2 result in elevated ERK1/2 activation and perturbation of neuronal differentiation. Biochem. J. *410*, 205-211.

Schulz, P. E., McIntosh, A. D., Kasten, M. R., Wieringa, B., and Epstein, H. F. (2003). A role for myotonic dystrophy protein kinase in synaptic plasticity. J. Neurophysiol. *89*, 1177-1186.

Schwamborn, J. C., Fiore, R., Bagnard, D., Kappler, J., Kalschmidt, C., and Püschel, A. W. (2004). Semaphorin 3A stimulates neurite extension and regulates gene expression in PC12 cells. J. Biol. Chem. *279*, 30923-30926.

Seipel, K., Medley, Q. G., Kedersha, N. L., Zhang, X. A., O'Brien, S. P., Serra-Pages, C., Hemler, M. E., and Streuli, M. (1999). Trio amino-terminal guanine nucleotide exchange factor domain expression promotes actin cytoskeleton reorganization, cell migration and anchorage-independent cell growth. J. Cell. Sci. *112*, 1825-1834.

Senocak, E. U., Oğuz, K. K., Haliloğlu, G., Topçu, M., and Cila, A. (2010). Structural abnormalities of the brain other than molar tooth sign in Joubert syndrome-related disorders. Diagn. Interv. Radiol. *16*, 3-6.

Seranski, P. Hoff, C., Radelof, U., Hennig, S., Reinhardt, R., Schwartz, C. E., Heiss, N. S., and Poustka, A. (2001). RAI1 is a novel polyglutamine encoding gene that is deleted in Smith-Magenis syndrome patients. Gene *270*, 69-76.

Sharifi, R., Morra, R., Appel, C. D., Tallis, M., Chioza, B., Jankevicius, G., Simpson, M. A., Matic, I., Ozkan, E., Golia, B., et al. (2013). Deficiency of terminal ADP-ribose protein glycohydrolase TARG1/C6orf130 in neurodegenerative disease. EMBO J. *32*, 1225-1237.

Sharma, N., Jadhav, S. P. and Bapat, S. A. (2010). CREBBP re-arrangements affect protein function and lead to aberrant neuronal differentiation. Differentiation *79*, 218-231.

Sharma, S., Koh, K. S., Collin, C., Dave, A., McMellon, A., Sugiyama, Y., McAvoy, J. W., Voss, A. K., Gécz, J., and Craig, J. E. (2009). NHS-A isoform of the NHS gene is a novel interactor of ZO-1. Exp. Cell. Res. *315*, 2358-2372.

Sheen, V. L., Torres, A. R., Du, X., Barry, B., Walsh, C. A., and Kimonis, V. E. (2010). Mutation in PQBP1 is associated with periventricular heterotopia. Am. J. Med. Genet. *152A*, 2888-2889.

Shen, Y., Inoue, N., and Heese, K. (2010). Neurotrophin-4 (ntf4) mediates neurogenesis in mouse embryonic neural stem cells through the inhibition of the signal transducer and activator of transcription-3 (stat3) and the modulation of the activity of protein kinase B. Cell Mol. Neurobiol. *30*, 909-916.

Shi, L., Chang, X., Zhang, P., Coda, M. P., Lu, W., and Wang, K. (2013). The functional genetic link of NLGN4X knockdown and neurodevelopment in neural stem cells. Hum. Mol. Genet. *22*, 3749-3760.

Shi, G.-X., Rehmann, H., and Andres, D. A. (2006). A novel cyclic AMP-dependent Epac-Rit signaling pathway contributes to PACAP38-mediated neuronal differentiation. Mol. Cell. Biol. *26*, 9136-9147.

Shimizu, T., Nakazawa, M., Kani, S., Bae, Y. K., Shimizu, T., Kageyama, R., and Hibi, M. (2010). Zinc finger genes Fezf1 and Fezf2 control neuronal differentiation by repressing Hes5 expression in the forebrain. Development *137*, 1875-1885.

Shinjyo, N., Ståhlberg, A., Dragunow, M., Pekny, M., and Pekna, M. (2009). Complement-derived anaphylatoxin C3a regulates in vitro differentiation and migration of neural progenitor cells. Stem Cells *27*, 2824-2832.

Shioda, N., Beppu, H., Fukuda, T., Li, E., Kitjima, I., and Fukunaga, K. (2011). Aberrant calcium/calmodulin-dependent protein kinase II (CaMKII) activity is associated with abnormal dendritic spine morphology in the ATRX mutant mouse brain. J. Neurosci. *31*, 346-358.

Shmueli, O., Gdalyahu, A., Sorokina, K., Nevo, E., Avivi, A., and Reiner, O. (2001). Hum. Mol. Genet. *10*, 1061-1070.

Shoubridge, C., Walikonis, R. S., Gécz, J., and Harvey, R. J. (2010). Subtle functional defects in the Arf-specific guanine nucleotide exchange factor IQSEC2 cause non-syndromic X-linked intellectual disability. Small GTPases *1*, 98-103.

Silei, V., Politi, V., and Lauro, G. M. (2000). Uridine induces differentiation in human neuroblastoma cells via Protein Kinase C episilon. J. Neurosci. Res. *61*, 206-211.

So, P.-L., Yip, P. K., Bunting, S., Wong, L.-F., Mazarakis, N. D., Hall, S., McMahon, S., Maden, M., and Corcoran, J. P. T. (2006). Interactions between retinoic acid, nerve growth factor and sonic hedgehog signaling in neurite outgrowth. Dev. Biol. *298*, 167-175.

Sobczak, M., Boczek, T., Kowalski, A., Wiktorska, M., Niewiarowska, J., and Zylinska, L. (2014). Downregulation of microsomal glutathione-S-transferase 1 modulates protective mechanisms in differentiated PC12 cells. J. Physiol. Biochem. *70*, 375-383.

Song, J. H., Wang, C. X., Song, D. K., Wang, P., Shuaib, A., and Hao, C. (2005). Interferon gamma induces neurite outgrowth by up-regulation of p35 neuron-specific cyclin-dependent kinase 5 activator via activation of ERK1/2 pathway. J. Biol. Chem. *280*, 12896-12901.

Soucek, T., Hölzl, G., Bernaschek, G., and Hengstschläger, M. (1998). A role of the tuberous sclerosis gene-2 product during neuronal differentiation. Oncogene *16*, 2197-2204.

Spassky, N., Han, Y. G., Aguilar, A., Strehl, L., Besse, L., Laclef, C., Ros, M. R., Garcia-Verdugo, J. M., and Alvarez-Buylla, A. (2008). Primary cilia are required for cerebellar development and Shh-dependent expansion of progenitor pool. Dev. Biol. *317*, 246-259.

Sprengel, R. (2006). Role of AMPA receptors in synaptic plasticity. Cell Tissue Res. *326*, 447-455.

Stornetta, R. L., and Zhu, J. J. (2011). Ras and Rap signaling in synaptic plasticity and mental disorders. Neuroscient *17*, 54-78.

Strauss, K. A., Puffenberger, E. G., Huentelman, M. J., Gottlieb, S., Dobrin, S. E., Parod, J. M., Stephan, D. A., and Morton, D. H. (2006). Recessive symptomatic focal epilepsy and mutant contactin-associated protein-like 2. N. Engl. J. Med. *354*, 1370-1377.

Sun, P., Quan, Z., Zhang, B., Wu, T., and Xi, R. (2010). TSC1/2 tumour suppressor complex maintains *Drosophila* germline stem cells by preventing differentiation. Development *137*, 2461-2469.

Sun, C., Warland, D. K., Ballesteros, J. M., van der List, D., and Chalupa, L. M. (2008). Retional waves in mice lacking the beta2 subunit of the nicotinic acetylcholine receptor. Proc. Natl. Acad. Sci. USA *105*, 13638-13643.

Suzuki, M., Nelson, A. D., Eickstaedt, J. B., Wallace, K., Wright, L. S., and Svendsen, C. N. (2006). Glutamate enhances proliferation and neurogenesis in human neural progenitor cell cultures derived from the fetal cortex. Eur. J. Neurosci. *24*, 645-653.

Tabata, H., and Nakajima, K. (2002). Neurons tend to stop migration and differentiate along the cortical internal plexiform zones in the Reelin signal-deficient mice. J. Neurosci. Res. *69*, 723-730.

Takeichi, M. (1991). Cadherin cell adhesion receptors as a morphogenetic regulator. Science *251*, 1451-1455.

Talkowski, M. E., Mullegama, S. V., Rosenfeld, J. A., van Bon, B. W., Shen, Y., Repnikova, E. A., Gastier-Foster, J., Thrush, D. L., Kathiresan, S., Ruderfer, D. M. (2011). Assessment of 2q23.1 microdeletion syndrome implicates MBD5 as a single causal locus of intellectual disability, epilepsy, and autism spectrum disorder. Am. J. Hum. Genet. *89*, 551-563.

Tang, Z. Z., Yarotskyy, V., Wei, L., Sobczak, K., Nakamori, M., Eichinger, K., Moxley, R. T., Dirksen, R. T., and Thornton, C. A. (2012). Muscle weakness is myotonic dystrophy associated with misregulated splicing and altered gating of Ca(V)1.1 calcium channel. Hum. Mol. Genet. *21*, 1312-1324.

Thambirajah, A. A., Ng, M. K., Frehlick, L. J., Li, A., Serpa, J. J., Petrotchenko, E. V., Silva-Moreno, B., Missiaen, K. K., Borchers, C. H., Adam Hall, J., et al. (2012). MeCP2 binds to nucleosome free (linker DNA) regions and to H3K9/H3K27 methylated nucleosomes in the brain. Nucleic Acids Res. *40*, 2884-2897.

Thibault, O., Hadley, R., and Landfield, P. W. (2001). Elevated postsynaptic [Ca2+]I and L-type calcium channel activity in aged hippocampal neurons: relationship to impaired synaptic plasticity. J. Neurosci. *21*, 9744-9756.

Toyo-oka, K., Shionoya, A., Gambello, M. J., Cardoso, C., Leventer, R., Ward, H. L., Ayala, R., Tsai, L.-H., Dobyns, W., Ledbetter, D., et al. (2003). 14-3-3-ε is important for neuronal migration by binding to NUDEL: a molecular explanation for Miller-Dieker syndrome. Nat. Genet. *34*, 274-285.

Tozuka, Y., Fukuda, S., Namba, T., Seki, T., and Hisatsune, T. (2005). GABAergic excitation promotes neuronal differentiation in adult hippocampal progenitor cells. Neuron *47*, 803-815.

Tsang, H. T., Edwards, T. L., Wang, X., Connell, J. W., Davies, R. J., Durrington, H. J., O'Kane, C. J., Luzio, J. P., and Reid, E. (2009). The hereditary spastic paraplegia proteins NIPA1, spastin and spartin are inhibitors of mammalian BMP signaling. Hum. Mol. Genet. *18*, 3805-3821.

Tucker, B., Richards, R. I., and Lardelli, M. (2006). Contribution of mGluR and Fmr1 functional pathways to neurite morphogenesis, craniofacial development and fragile X syndrome. Hum. Mol. Genet. *15*, 3446-3458.

Ueberham, U., and Arendt, T. (2013). The role of Smad proteins for development, differentiation and dedifferentiation of neurons. Accessed on 8/28/2014 from http://www.intechopen.com/books/trends-in-cell-signaling-pathways-in-neuronal-fate-decision/the-role-of-smad-proteins-for-development-differentiation-and-dedifferentiation-of-neurons

Uhrbom, L., Dai, C., Celestino, J. C., Rosenblum, M. K., Fuller, G. N., and Holland, E. C. (2002). Ink4a-Arf loss cooperates with KRas activation in astrocytes and neural progenitors to generate glioblastomas of various morphologies dependeing on activated Akt. Cancer Res. *62*, 5551-5558.

Valenciano, A. I., Mayordomo, R., de La Rosa, E. J., and Hallböök, F. (2002). Biotin decreases retinal apoptosis and induces eye malformations in the early chick embryo. Neuroreport *13*, 297-299.

Valli, E., Trazzi, S., Fuchs, C., Erriquez, D., Bartesaghi, R., Perini, G., and Ciani, E. (2012). CDKL5, a novel MYCN-repressed gene, blocks cell cycle and promotes differentiation of neuronal cells. Biochim. Biophys. Acta *1819*, 1173-1185.

Vallipuram, J., Grenville, J., and Crawford, D. A. (2010). The E646D-ATP13A4 mutation associated with autism reveals a defect in calcium regulation. Cell. Mol. Neurobiol. *30*, 233-246.

Valnegri, P., Montrasio, C., Brambilla, D., Ko, J., Passafaro, and Sala, C. (2011). The X-linked intellectual disability protein IL1RAPL1 regulates excitatory synapse formation by binding PTPδ and RhoGAP2. Hum. Mol. Genet. *20*, 4797-4809.

Van Maldergem, L., Hou, Q., Kalscheuer, V. M., Rio, M., Doco-Fenzy, M., Medeira, A., de Brouwer, A. P., Cabrol, C., Haas, S. A., Cacciagli, P., et al. (2013). Loss of function of KIAA2022 causes mild to severe intellectual disability with an autism spectrum disorder and impairs neurite outgrowth. Hum. Mol. Genet. *22*, 3306-3314.

Vardya, I., Drasbek, K. R., Gibson, K. M., and Jensen, K. (2010). Plasticity of postsynaptic, but not presynaptic, $GABA_B$ receptors in SSADH deficient mice. Exp. Neurol. *225*, 114-122.

Veldman, M. B., Bemben, M. A., and Goldman, D. (2010). Tuba1a gene expression is regulated by KLF6/7 and is necessary for CNS development and regeneration in zebrafish. Mol. Cell. Neurosci. *43*, 370-383.

Vernes, S. C., Oliver, P. L., Spiteri, E., Lockstone, H. E., Puliyadi, R., Taylor, J. M., Ho, J., Mombereau, C., Brewer, A., Lowy, E., et al. (2011). PLoS Genet. *7*, e1002145.

Verrotti, A., Spalice, A., Ursitti, F., Papetti, L., Mariani, R., Castronovo, A., Mastrangelo, M., and Iannetti, P. (2010). New trends in neuronal migration disorders. Eur. J. Paediatr. Neurol. *14*, 1-12.

Vogel-Ciernia, A., Matheos, D. P., Barrett, R. M., Kramár, E. A., Azzawi, S., Chen, Y., Magnan, C. N., Zeller, M., Sylvain, A., Haetig, J., et al. (2013). The neuron-specific chromatin regulatory subunit BAF53b is necessary for synaptic plasticity and memory. Nat. Neurosci. *16*, 552-561. Wiley Interdis. Rev. Membr. Transp. Sig. *3*, 1-13.

Volk, C. (2014). OCTs, OATs, and OCTNs: structure and function of the polyspecific organic ion transporters of the SLC22 family.

Völkel, P., and Angrand, P. O. (2007). The control of histone lysine methylation in epigenetic regulation. Biochimie *89*, 1-20.

Walton, N. M., de Koning, A., Xie, X., Shin, R., Chen, Q., Miyake, S., Tajinda, K., Gross, A. K., Kogan, J. H., Heusner, C. L., et al. (2014). Gastrin-releasing peptide contributes to the regulation of adult hippocampal neurogenesis and neuronal development. Stem Cells *32*, 2454-2466.

Wang, W., Crandall, J. E., Litwack, E. D., Gronostajski, R. M., and Kilpatrick, D. L. (2010). Targets of the nuclear factor I regulon involved in early and late development of postmitotic cerebellar granule neurons. J. Neurosci. Res. *88*, 258-265.

Wang, J., Leung, J. W., Gong, Z., Feng, L., Shi, X., and Chen, J. (2013a). PHF6 regulates cell cycle progression by suppressing ribosomal RNA synthesis. J. Biol. Chem. *288*, 3174-3183.

Wang, Q., Moore, M. J., Adelmant, G., Marto, J. A., and Silver, P. A. (2013b). PQBP1, a factor linked to intellectual disability, affects alternative splicing associated with neurite outgrowth. Genes Dev. *27*, 615-626.

Wang, P. Y., Seabold, G. K., and Wenthold, R. J. (2008). Synaptic adhesion-like molecules (SALMs) promote neurite outgrowth. Mol. Cell. Neurosci. *39*, 83-94.

Wang, X., Xu, Q., Bey, A. L., Lee, Y., and Jiang, Y. H. (2014). Transcriptional and functional complexity of Shank3 provides a molecular framework to understand the phenotypic heterogeneity of SHANK3 causing autism and Shank3 mutant mice. Mol. Autism *5*, 30.

Wang, X., Yang, N., Uno, E., Roeder, R. G., and Guo, S. (2006). A subunit of the mediator complex regulates vertebrate neuronal development. Proc. Natl. Acad. Sci. USA *103*, 17284-17289.

Watanabe, Y., Inoue, K., Okuyama-Yamamoto, A., Nakai, N., Nakatani, J., Nibu, K., Sato, N., Iiboshi, Y., Yusa, K., Kondoh, G., et al. (2009). Fezf1 is required for penetration of the basal lamina by olfactory axons to promote olfactory development. J. Comp. Neurol. *515*, 565-584.

Weimann, J. M., Zhang, Y. A., Levin, M. E., Devine, W. P., Brûlet, W. P., and McConnell, S. K. (1999). Cortical neurons require Oxt1 for the refinement of exuberant axonal projections to subcortical targets. Neuron *24*, 819-831.

Weng, L., Lin, Y. F., Li, A. L., Wang, C. E., Yan, S., Sun, M., Gaertig, M. A., Mitha, N., Kosaka, J., Wakabayashi, T., et al. (2013). Loss of Ahi1 affects early development by impairing BM88/Cend1-mediated neuronal differentiation. J. Neurosci. *33*, 8172-8184.

White, D. M., Walker, S., Brenneman, D. E., and Gozes, I. (2000). CREB contributes to the increased neurite outgrowth of sensory neurons induced by vasoactive intestinal polypeptide and activity-dependent neurotrophic factor. Brain Res. *868*, 31-38.

Whitehead, J., and Cobo, E. (2008). Stopping a trial early in oncology: for patients or for industry? Ann. Oncol. *19*, 1512-1513.

Whyte, A. J. (2014). Genetic and functional interactions between Itgb3 and Slc6a4 in mouse brain. Hosted by Vanderbilt University. Accessed on 8/27/2014 from http://etd.library.vanderbilt.edu/available/etd-03212013-135948/

Williams, M. E., Wilke, S. A., Daggett, A., Davis, E., Otto, S., Ravi, D., Ripley, B., Bushong, E. A., Ellisman, M. H., Klein, G., et al. (2011). Cadherin-9 regulates synapse-specific differentiation in the developing hippocampus. Neuron *71*, 640-655.

Wittwer, F., van der Straten, A., Keleman, K., Dickson, B. J., and Hafen, E. (2001). Lilliputian: an AF4/FMR2-related protein that controls cell identity and cell growth. Development *128*, 791-800.

Wolfer, D. P., Lang, R., Cinelli, P., Madani, R., and Sonderegger, P. (2001). Multiple roles of neurotrypsin in tissue morphogenesis and nervous system development suggested by the mRNA expression pattern. Mol. Cell. Neurosci. *18*, 407-433.

Wu, P. R., Tsai, P. I., Chen, G. C., Chou, H. J., Huang, Y. P., Chen, Y. H., Lin, M. Y., Kimchi, A., Chien, C. T., and Chen, R. H. (2011). DAPK activates MARK1/2 to regulate microtubule assembly, neuronal differentiation, and tau toxicity. Cell Death Differ. *18*, 1507-1520.

Xiao, Y., Peng, Y., Wan, J., Tang, G., Chen, Y., Tang, J., Ye, W. C., Ip, N. Y., and Shi, L. (2013). The atypical guanine nucleotide exchange factor Dock4 regulates neurite differentiation through modulation of Rac1 GTPase and actin dynamics. J. Biol. Chem. *288*, 20034-20045.

Yabut, O., Domagauer, J., and D'Arcangelo, G. (2010). Dyrk1A overexpression inhibits proliferation and induces premature neuronal differentiation of neural progenitor cells. J. Neurosci. *30*, 4004-4014.

Yasin, S. A., Ali, A. M., Tata, M., Picker, S. R., Anderson, G. W., Latimer-Bowman, E., Nicholson, S. L., Harkness, W., Cross, J. H., Paine, S. M., et al. (2013). mTOR-dependent abnormalities in autophagy characterize human malformations of cortical development: evidence from focal cortical dysplasia and tuberous sclerosis. Acta Neuropathol. *126*, 207-218.

Yoon, C., Van Niekerk, E. A., Henry, K., Ishikawa, T., Orita, S., Tuszynski, M. H., and Campana, W. M. (2013). Low-density lipoprotein receptor-related protein 1 (LRP1)-dependent cell signaling promotes axonal regeneration. J. Biol. Chem. *288*, 26557-26568.

Yoshiga, D., Taketomi, T., Muratsu, D., Onimura, T., Shiraishi, H., Sansada, T., Kobayashi, T., and Nakamura, S. (2013). RNF135, a RING finger protein, negatively regulates RAS/MAPK signaling. IADR General Session. Accessed on 8/29/2014 from https://iadr.confex.com/iadr/2010barce/webprogram/Paper136847.html

Yoshihara, S., Omichi, K., Yanazawa, M., Kitamura, K., and Yoshihara, Y. (2005). Arx homeobox gene is essential for development of mouse olfactory system. Development *132*, 751-762.

Yu, I. T., Park, J. K., Kim, S. H., Lee, J. S., Kim, Y. S., and Son, H. (2009). Valproic acid promotes neuronal differentiation by induction of proneural factors in association with H4 acetylation. Neuropharmacology *56*, 472-480.

Yu, H., and Patel, S. B. (2005). Recent insights into the Smith-Lemli-Opitz syndrome. Clin. Genet. *68*, 383-391.

Yu, H., Wessels, A., Tint, G. S., and Patel, S. B. (2005). Partial rescue of neonatal lethality of Dhcr7 null mice by a nestin promoter-driven DHCR7 transgene expression. Brain Res. Dev. Brain Res. *156*, 46-60.

Zeng, L., Zhang, P., Shi, L., Yamamoto, V., Lu, W., and Wang, K. (2013). Functional impacts of NRXN1 knockdown on neurodevelopment in stem cell models. PLoS One *8*, e59685.

Zhang, Y., Chen, D., and Wang, Z. (2009). Analyses of mental dysfunction-related ACSL4 in drosophila reveal its requirement for Dpp/BMP production and visual wiring in the brain. Hum. Mol. Genet. *18*, 3894-3905.

Zhang, C., Mejia, L. A., Huang, J., Valnegri, P., Bennett, E. J., Anckar, J., Jahani-Asl, A., Gallardo, G., Ikeuchi, Y., Yamada, T., et al. (2013). The X-linked intellectual disability protein PHF6 associates with the PAF1 complex and regulates neuronal migration in the mammalian brain. Neuron *78*, 986-993.

Zhang, L., Song, N. N., Chen, J. Y., Huang, Y., Li, H., and Ding, Y. Q. (2012). Satb2 is required for dendritic arborization and soma spacing in mouse cerebral cortex. Cereb. Cortex *22*, 1510-1519.

Zhang, K. Z., Westberg, J. A., Hölttä, E., and Andersson, L. C. (1996). BCL2 regulates neural differentiation. Proc. Natl. Acad. Sci. USA *93*, 4504-4508.

Zhang, Y. Y., Yue, J., Che, H., Sun, H. Y., Tse, H. F., and Li, G. R. (2014). BKCa and hEag1 channels regulate cell proliferation and differentiation in human bone marrow-derived mesenchymal stem cells. J. Cell Physiol. *229*, 202-212.

Zhang-James, Y., DasBanerjee, T., Sagvolden, T., Middleton, F. A., and Faraone S. V. (2011). SLC9A9 mutations, gene expression, and protein-protein interactions in rat models of attention-deficit/hyperactivity disorder. Am. J. Med. Genet. B Neuropsychiatr. Genet. *156B*, 835-843.

Zhu, B., Chen, C., Xue, G., Moyzis, R. K., Dong, Q., Chen, C., He, Q., Lei, X., Wang, Y., et al. (2013a). The SEMA5A gene is associated with hippocampal volume, and their interaction is associated with performance on Raven's Progressive Matrices. Neuroimage *88C*, 181-187.

Zhu, Y. C., Li, D., Wang, L., Lu, B., Zheng, J., Zhao, S. L., Zeng, R., and Xiong, Z. Q. (2013b). Palmitoylation-dependent CDKL5-PSD-95 interaction regulates synaptic targeting of CDKL5 and dendritic spine development. Proc. Natl. Acad. Sci. USA *110*, 9118-9123.

Zhu, X., Morales, F. C., Agarwal, N. K., Dogruluk, T., Gagea, M., Georgescu, M. M. (2013c). Moesin is a glioma progression marker that induces proliferation and Wnt/beta-catenin pathway activation via interaction with CD44. Cancer Res. *73*, 1142-1155.

Zilberberg, A., Yaniv, A., and Gazit, A. (2004). The low density lipoprotein receptor-1, LRP1, interacts with the human frizzled-1 (HFz1) and down-regulates the canonical Wnt signaling pathway. J. Biol. Chem. *279*, 17535-17542.

Supplementary Table 2. Gene indications in epilepsy and schizophrenia for each high-risk autism gene. Available citations for epilepsy indicate a positive "hit" in the literature. "Yes/No" indicates whether a gene is contained within the *SzGene* Database.

Aut Gene	Indicated in Epilepsy	Contained in SzGene Database
ACSL4		Yes
ADNP	Cosgrave et al. (2008)	No
ADSL	Marie et al. (1999)	Yes
AFF2	Coffee et al. (2008)	No
AGTR2	Ylisaukko-oja et al. (2004)	No
AHI1	Dixon-Salazar	Yes
ALDH5A1	Knerr et al. (2007)	Yes
ALDH7A1	Plecko et al. (2007)	No
AP1S2	Tarpey et al. (2006)	No
ARHGEF6		No
ARID1B		No
ARX	Kato et al. (2007)	No
ASMT		No
ATP13A4		No
ATRX	Akahoshi et al. (2005)	No
AUTS2	Mefford et al. (2010)	No
AVPR1A		No
BCL2		No
BDNF	Scharfman et al. (2002)	Yes
BRAF	Adachi et al. (2012)	No
BTD	Salbert et al. (1993)	No
C4B		Yes
CACNA1C		Yes
CACNA1E	Rijkers et al. (2010)	No
CACNA1F		Yes
CACNA1H	Chen et al. (2003)	No
CADM1		No
CASK	Saitsu et al. (2012)	No
CDH10		No
CDH9		No
CDKL5	Archer et al. (2006)	No
CEP290	Şenocak et al. (2010)	No
CHD7	Lalani et al. (2009)	No
CHD8		No
CHRNA4	Hirose et al. (1999)	Yes

CHRNB2	Phillips et al. (2001)	Yes
CNTN4	Roohi et al. (2009)	No
CNTNAP2	Mefford et al. (2010)	No
COMT	Doyle & Sellinger (1980)	Yes
CREBBP	Seltzer & Paciorkowski (2014)	No
CYFIP1		No
DBH	Browning et al. (1989)	Yes
DCX	Kerjan et al. (2009)	No
DHCR7	Johnson (1975)	No
DISC1		Yes
DMD	De Sarro et al. (2004)	No
DMPK		No
DNAH5		No
DRD3		Yes
DRD4	Rubenstein et al. (2001)	Yes
DYRK1A	Courcet et al. (2012)	No
EHMT1	Kleefstra et al. (2006)	No
EIF4E		No
EN2	Tripathi et al. (2008)	No
FBXO33	Flood et al. (2004)	No
FEZF1		No
FEZF2	Lodato et al. (2011)	No
FGD1		No
FGFR2		Yes
FHIT		No
FMR1	Musemeci et al. (2007)	No
FOXG1	Brunetti-Pierri et al. (2011)	No
FOXP1	Carr et al. (2010)	No
FOXP2	Turner et al. (2013)	Yes
FTSJ1	Takano et al. (2008)	No
GABRA4	Roberts et al. (2005)	No
GABRA5	Di Rocco et al. (2013)	Yes
GABRB1		No
GABRB3	DeLorey & Olsen (1999)	No
GABRG3	Pelc et al. (2008)	No
GAD1	Darrah et al. (2013)	Yes
GAMT	Mercimek-Mahmutoglu et al. (2006)	No
GATM	Stöckler et al. (2014)	No
GRIA3	Bonnet et al. (2012)	Yes
GRIK2	Mulle et al. (1998)	Yes
GRIN2B	Lemke et al. (2014)	Yes

GRIN3B		No
GRIP1		Yes
GRPR		No
GSE1		No
GSTM1	Liu & Tsai (2002)	Yes
GUCY2D		No
HLA-DRB1	Jain et al. (1999)	No
HOXA1		No
HRAS	Della Marca et al. (2011)	No
IGF2	Dikkes et al. (2007)	Yes
IL1RAPL1	Dinopoulos et al. (2014)	No
IMMP2L		No
IQSEC2	Gandomi et al. (2014)	No
ITGB3		No
KCND2	Lee et al. (2014)	No
KCNMA1	Ermolinsky et al. (2008)	No
KDM5C	Poeta et al. (2013)	No
KIAA0100		No
KIAA2022	Cantagrel et al. (2004)	No
KRAS	Adachi et al. (2012)	No
L1CAM	Maruta et al. (1996)	Yes
L2HGDH	Vilarinho et al. (2010)	No
LAMP2		No
LRFN5	de Bruijn et al. (2010)	No
LRP1		No
MACROD2		No
MAOA	Teskey et al. (2004)	Yes
MAP2K1	Nateri et al. (2007)	No
MAPK1	Nateri et al. (2007)	No
MAPK3		No
MARK1		No
MBD5	Talkowski et al. (2011)	No
MECP2	Jian et al. (2007)	No
MED12	Van Buggenhout & Fryns (2006)	Yes
MEF2C	Nowakowska et al. (2010)	No
MEGF11		No
MET	Bae et al (2010)	Yes
MID1		No
MKKS		No
MSNP1AS		No
NDP	Lev et al. (2007)	No

NF1	Kulkantrakorn & Geller (1998)	No
NFIX	Yoneda et al. (2012)	No
NHS		No
NIPA1	Svenstrup et al. (2011)	No
NIPBL	Pavlidis et al. (2014)	No
NLGN3		No
NLGN4X		No
NPHP1	Bartnik et al. (2012)	No
NRXN1	Harrison et al. (2011)	Yes
NRXN2		No
NSD1	Tatton-Brown & Nazneen (2004)	No
NTF3	Risbud et al. (2011)	Yes
NTF4		No
OCRL	Ramirez et al. (2012)	No
OPHN1	Santos-Rebouças et al. (2014)	No
OTC	Bogdanovic et al. (2000)	No
OTX1	Acampora et al. (1996)	No
OXTR	Sala et al. (2011)	Yes
PAFAH1B1	Greenwood et al. (2009)	Yes
PAH	Martynyuk et al. (2007)	Yes
PCDH19	Depienne et al. (20009)	No
PHF6	Lower et al. (2002)	No
PHF8		No
PITX1		No
POGZ		No
POMGNT1	Teber et al. (2008)	No
POMT1	Mochida (2008)	No
PQBP1	Stevenson et al. (1998)	No
PRKCB	Szepetowski & Monaco (1998)	No
PRKX		No
PRSS12		No
PTCHD1		No
PTEN	Backman et al. (2001)	Yes
PTPN11	de Jong et al. (2011)	No
RAB39B	Giannandrea et al. (2010)	No
RAI1	Goldman (2006)	No
RAPGEF4	Zhao et al. (2013)	No
RBFOX1	Lal et a. (2013)	No
RELN	Gong et al. (2007)	Yes
RIMS3		No
RNF135		No

RPE65		No
RPGRIP1L	Şenocak et al. (2010)	Yes
RPL10		No
RPS6KA3	Matsumoto et al. (2013)	No
SATB2	Leoyklang et al. (2007)	No
SBF1		No
SCN1A	Claes et al. (2001)	No
SCN2A	Herlenius et al. (2007)	No
SEMA5A	Yang et al. (2005)	No
SEZ6L2	Guerra (2011)	No
SGSH	OMIM (2014)	No
SHANK2		No
SHANK3	Han et al. (2013)	Yes
SLC22A9		No
SLC25A12	Falk et al. (2014)	Yes
SLC6A4	Hrvoje et al. (2010)	Yes
SLC6A8	Fons et al. (2009)	No
SLC9A6	Gilfillan et al. (2008)	No
SLCO1C1		No
SMC1A	Baquero-Montoya et al. (2014)	No
ST8SIA2	Kamien et al. (2014)	Yes
SUV420H1		No
SYN1	Fassio et al. (2011)	No
TBR1	Traylor et al. (2012)	No
TBX1		Yes
TRIO		No
TSC1	Meikle et al. (2007)	No
TSC2	Chu-Shore et al. (2009)	Yes
TTN		No
TUBA1A	Sohal et al. (2012)	Yes
TUBGCP5		No
UBE3A	Minassian et al. (1998)	No
UPF3B		No
UPP2		No
VIP	Marksteiner et al. (1989)	No
VPS13B		No
YWHAE		Yes
ZNF674		No
ZNF81		No

References for Supplementary Table 2

Acampora, D., Mazan, S., Avantaggiato, V., Barone, P., Tuorto, F., Lallemand, Y., Brûlet, P., and Simeone, A. (1996). Epilepsy and brain abnormalities in mice lacking the *Otx1* gene. Nat. Genet. *14*, 218-222.

Adachi, M., Abe, Y., Aoki, Y., and Matsubara, Y. (2012). Epilepsy in RAS/MAPK syndrome: two cases of cardio-facio-cutaneous syndrome with epileptic encephalopathy and a literature review. Seizure *21*, 55-60.

Akahoshi, K., Ohashi, H., Hattori, Y., Saitoh, S., Fukushima, Y., and Wada, T. (2005). A woman with 46,XX,dup(16)(p13.11-p13.3) and the ATR-X phenotype. Am. J. Med. Genet. Part A *132A*, 414-418.

Archer, H. L., Evans, J., Edwards, S., Colley, J., Newbury-Ecob, R., O'Callaghan, F., Huyton, M., O'Regan, M., Tolmie, J., et al. (2006). J. Med. Genet. *43*, 729-734.

Backman, S. A., Stambolic, V., Suzuki, A., Haight, J., Elia, A., Pretorius, J., Tsao, M.-S., Shannon, P., Bolon, B., Ivy, G. O., et al. (2001). Deletion of *Pten* in mouse brain causes seizures, ataxia and defects in soma size resembling Lhermitte-Duclos disease. Nat. Genet. *29*, 396-403.

Bae, M. H., Bissonette, G. B., Mars, W. M., Michalopoulos, G. K., Achim, C. L., Depireux, D. A., and Powell, E. M. (2010). Hepatocyte growth factor (HGF) modulates GABAergic inhibition and seizure susceptibility. Exp. Neurol. *221*, 129-135.

Baquero-Montoya, C., Gil-Rodríquez, M. C., Teresa-Rodrigo, M. E., Hernández-Marcos, M., Bueno-Lozano, G., Bueno-Martínez, I., Remeseiro, S., Fernández-Hernández, R., Bassecourt-Serra, M., et al. (2014). Could a patient with *SMC1A* duplication be classified as a human cohesinopathy? Clinical Genet. *85*, 446-451.

Bartnik, M., Szczepanik, E., Derwińska, K., Wiśniowiecka-Kowalnik, B., Gambin, T., Sykulski, M., Ziemkiewicz, K., Kędzio, M., Gos, M., Hoffman-Zacharska, D., et al. (2012). Application of array comparative genomic hybridization in 102 patients with epilepsy and additional neurodevelopmental disorders. Am. J. Med. Genet. B Neuropsychiatr. Genet. *159B*, 760-771.

Bogdanovic, M. D., Kidd, D., Briddon, A., Duncan, J. S., and Land, J. M. (2000). Late onset heterozygous ornithine transcarbamylase deficiency mimicking complex partial status epilepticus. J. Neurol. Neurosurg. Psychiatry *69*, 813-815.

Bonnet, C., Masurel-Paulet, A., Khan, Béri-Dexheimer, Callier, P., Mugneret, F., Philippe, C., Thauvin-Robinet, C., Faivre, L., et al. (2012). Exploring the potential role of disease-causing mutation in a gene desert: duplication of noncoding elements 5' of *GRIA3* is associated with *GRIA3* silencing and X-linked intellectual disability. Hum. Mutat. *33*, 355-358.

Browning, R. A., Wade, D. R., Marcinczyk, M., Long, G. L., and Jobe, P. C. (1989). Regional brain abnormalities in norepinephrine uptake and dopamine beta-hydroxylase activity in the genetically epilepsy-prone rat. J. Pharmacol. Exp. Ther. *249*, 229-235.

Brunetti-Pierri, N., Paciokowski, A. R., Ciccone, R., Mina, E. D., Bonaglia, M. C., Borgatti, R., Schaaf, C. P., Sutton, V. R., Xia, Z., Jelluma, N., et al. (2011). Duplications of *FOXG1* in 14q12 are associated with developmental epilepsy, mental retardation, and severe speech impairment. Euro. J. Hum. Genet. *19*, 102-107.

Cantagrel, V., Lossi, A. M., Boulanger, S., Depetris, D., Mattei, M. G., Gecz, J., Schwartz, C. E., Van Maldergem, L., and Villard, L. (2004). Disruption of a new X linked gene highly expressed in brain in a family with two mentally retarded males. J. Med. Genet. *41*, 736-742.

Carr, C. W., Moreno-De-Luca, D., Parker, C., Zimmerman, H. H., Ledbetter, N., Martin, C. L., Dobyns, W. B., and Abdul-Rahman, O. A. (2010). Chiari I malformation, delayed gross motor skills, severe speech delay, and epileptiform discharges in a child with *FOXP1* haploinsufficiency. Euro. J. Hum. Genet. *18*, 1216-1220.

Chen, Y., Lu, J., Pan, H., Zhang, Y., Wu, H., Xu, K., Liu, X., Jiang, Y., Bao, X., Yao, Z., et al. (2003). Association between genetic variation of *CACNA1H* and childhood absence epilepsy. Ann. Neurol. *54*, 239-243.

Chu-Shore, C., J., Major, P., Montenegro, M., and Thiele, E. (2009). Cyst-like tubers are associated with TSC2 and epilepsy in tuberous sclerosis complex. Neurology *72*, 1165-1169.

Claes, L., Del-Favero, D., Ceulemans, B., Lagae, L., Van Broeckhoven, C., and De Jonghe, P. (2001). De novo mutations in the sodium-channel gene *SCN1A* cause severe myoclonic epilepsy of infancy. Am. J. Hum. Genet. *68*, 1327-1332.

Coffee, B., Ikeda, M., Budimirovic, D. B., Hjelm, L. N., Kaufman, W. E., and Warren, S. T. (2008). Mosaic *FMR1* deletion causes fragile X syndrome and can lead to molecular misdiagnosis: a case report and review of the literature. Am. J. Med. Genet. *146A*, 1358-1367.

Cosgrave, A. S., McKay, J. S., Bubb, V., Morris, R., Quinn, J. P., and Thippeswamy T. (2008). Regulation of activity-dependent neuroprotective protein (ADNP) by the NO-cGMP pathway in the hippocampus during kainic acid-induced seizure. Neurobiol. Dis. *30*, 281-292.

Courcet, J.-B., Faivre, L., Malzac, P., Masurel-Paulet, A., Lopez, E., Callier, P., Lambert, L., Lemesle, M., Thevenon, J., Gigot, N., et al. (2012). The *DYRK1A* gene is a cause of syndromic intellectual disability with severe microcephaly and epilepsy. J. Med. Genet. *49*, 731-736.

Darrah, S. D., Miller, M. A., Ren, D., Hoh, N. Z., Scanlon, J. M., Conley, Y. P., and Wagner, A. K. (2013). Epilep. Res. *103*, 180-194.

de Bruijn, D. R., van Dijk, A. H., Pfundt, R., Hoischen, A., Merkx, G. F., Gradek, G. A., Lybæk, H., Stray-Pedersen, A., Brunner, H. G., and Houge, G. (2010). Severe progressive autism associated with two de novo changes: a 2.6-Mb 2q31.1 deletion and a balanced t(14;21)(q21.1;p11.2) translocation with long-range epigenetic silencing of LRFN5 expression. Mol. Syndromol. *1*, 46-57.

de Jong, M., Schieving, J., and Goraj, B. (2011). Remarkable intra-cerebral lesions on MRI in a patient with Noonan syndrome. Europ. J. Radiol. Extra *78*, e17-e19.

Della Marca, G., Leoni, C., Dittoni, S., Battaglia, D., Losurdo, A., Testani, E., Colicchio, S., Gnoni, V., Gambardella, M. L., Alfieri, P., et al. (2011). Increased sleep spindle activity in patients with Costello syndrome (HRAS gene mutation). J. Clin. Neurophysiol. *28*, 314-318.

DeLorey, T. M., and Olsen, R. W. (1999). GABA and epileptogenesis: comparing gabrb3 gene-deficient mice with Angelman syndrome in man. Epilep. Res. *36*, 123-132.

Depienne, C., Bouteiller, D., Keren, B., Cheuret, E., Poirier, K., Trouillard, O., Benyahio, B., Quelin, C., Carpentier, W., Julia, S., et al. (2009). Sporadic infantile epileptic encephalopathy caused by mutations in *PCDH19* resembles Dravet syndrome but mainly affects females. PLoS Genet. *5*, e1000381.

De Sarro, G., Ibbadu, G. F., Marra, R., Rotiroti, D., Loiacono, A., Di Paola, E. D., and Russo, E. (2004). Seizure susceptibility to various convulsant stimuli in dystrophin-deficient *mdx* mice. Neurosci. Res. *50*, 37-44.

Dikkes, P., Jaffe, D. B., Guo, W. H., Chao, C., Hemond, P., Yoon, K., Zurakowski, D., and Lopez, M. F. (2007). Brain Res. *1175*, 85-95.

Dinopoulos, A., Stefanou, M. I., Attilakos, A., Tsirouda, M., and Papaevangelou, V. (2014). A case of startle epilepsy associated with IL1RAPL1 gene deletion. Pediatr. Neurol. *51*, 271-274.

Dixon-Salazar, T., Silhavy, J. L., Marsh, S. E., Louie, C. M., Scott, L. C., Gururaj, A., Al-Gazali, L., Al-Tawari, A., Kayserili, H., Sztriha, L., et al. (2004). Mutations in the *AHI1* gene, encoding Joubering, cause Joubert syndrome with cortical polymicrogyria. Am. J. Hum. Genet. *75*, 979-987.

Di Rocco, A., Loggini, A., Di Rocco, M., Di Rocco, P., Rossi, R. P., Gimelli, G., and Bazil, C. (2013). Paradoxical worsening of seizure activity with pregabalin in an adult with isodicentric 15 (IDIC-15) syndrome involving duplications of the *GABRB3*, *GABRA5* and *GABRG3* genes. BMC Neurol. *13*, 43.

Doyle, R. L., and Sellinger, O. Z. (1980). Differences in activity in cerebral methyltransferases and monoamine oxidases between audiogenic seizure susceptible and resistant mice and deermice. Pharm. Biochem. Beh. *13*, 589-591.

Ermolinsky, B., Arshadmansab, M. F., Pacheco Otalora, L. F., Zarei, M. M., and Garrido-Sanabria, E. R. (2008). Deficit of Kcnma1 mRNA expression in the dentate gyrus of epileptic rats. Neuroreport *19*, 1291-1294.

Falk, M. J., Li, D., Gai, X., McCormick, E., Place, E., Lasorsa, F. M., Otieno, F. G., Hou, C., Kim, C. E., et al. (2014). AGC1 deficiency causes infantile epilepsy, abnormal myelination, and reduced *N*-acetylaspartate. JIMD Reports, doi: 10.1007/8904_2013_287

Fassio, A., Patry, L., Congia, S., Onofri, F., Piton, A., Gauthier, J. Pozzi, D., Messa, M., Defranchi, E., Fadda, M., et al. (2011). *SYN1* loss-of-function mutations in autism and partial epilepsy cause impaired synaptic function. Hum. Mol. Genet. *20*, 2297-2307.

Flood, W. D., Moyer, R. W., Tsykin, A., Sutherland, G. R., and Koblar, S. A. (2004). *Nxf* and *Fbxo33*: novel seizure-responsive genes in mice. Euro. J. Neurosci. *20*, 1819-1826.

Fons, C., Sempere, Á., Sanmarti, F. X., Arias, Á, Póo, Pineda, M., Ribes, A., Merinero, B., Vilaseca, M. A., Salomons, G. S., et al. (2009). Epilepsy spectrum in cerebral creatine transporter deficiency. Epilepsia *50*, 2168-2170.

Gandomi, S. K., Farwell Gonzalez, K. D., Parra, M., Shahmirzadi, L., Mancuso, J., Pinchurin, P., Temme, R., Dugan, S., Zeng, W., and Tang, S. (2014). Diagnostic exome sequencing identifies two novel IQSEC2 mutations associated with X-linked intellectual disability with seizures: implications for genetic counseling and clinical diagnosis. J. Genet. Couns. *23*, 289-298.

Giannandrea, M., Bianchi, V., Mignogna, M. L., Sirri, A., Carrabino, S., D'Elia, E., Vecellio, M., Russo, S., Cogliati, F., Larizza, L. Ropers, H.-H., et al. (2010). Mutations in the small GTPase gene *RAB39B* are responsible for X-linked mental retardation associated with autism, epilepsy, and macrocephaly. Am. J. Hum. Genet. *86*, 185-195.

Gilfillan, G. D., Selmer, K. K., Roxrud, I., Smith, R., Kyllerman, M., Eiklid, K., Kroken, M., Mattingsdal, M., Egeland, T., Stenmark, H., et al. (2008). *SLC9A6* mutations caused X-linked mental retardation, microcephaly, epilepsy, and ataxia, a phenotype mimicking Angelman syndrome. Am. J. Hum. Genet. *82*, 1003-1010.

Gong, C., Wang, T.-W., Huang, H. S., and Parent, J. M. (2007). Reelin regulates neuronal progenitor migration in intact and epileptic hippocampus. J. Neurosci. *27*, 1803-1811.

Goldman, A. M. (2006). Topical review: epilepsy and chromosomal rearrangements in Smith-Magenis syndrome [del(17)(p11.2p11.2)]. J. Child Neurol. *21*, 93-98.

Greenwood, J. S. F., Wang, Y., Estrada, R. C., Ackerman, L., Ohara, P. T., and Baraban, S. C. (2009). Seizures, enhanced excitation, and increased vesicle number of *Lis1* mutant mice. Ann. Neurol. *66*, 644-653.

Guerra, D. J. (2011). The molecular genetics of autism spectrum disorders: genomic mechanisms, neuroimmunopathology, and clinical implications. Autism Res. Treat. *2011*, 398636.

Han, K., Holder, J. L., Schaaf, C. P., Lu, H., Chen, H., Kang, H., Tang, J., Wu, Z., Hao, S., Cheung, S. W., et al. (2013). *SHANK3* overexpression causes manic-like behavior with unique pharmacogenetic properties. Nature *503*, 72-77.

Harrison, V., Connell, L., Hayesmoore, J., McParland, J., Pike, M. G., and Blair, E. (2011). Compound heterozygous deletion of *NRXN1* causing severe developmental delay with early onset epilepsy in two sisters. Am. J. Med. Genet. A *155A*, 2826-2831.

Herlenius, E., Heron, S. E., Grinton, B. E., Keay, D., Scheffer, I. E., Mulley, J. C., and Berkovic, S. (2007). *SCN2A* mutations and benign familial neonatal-infantile seizures: the phenotypic spectrum. Epilesia *48*, 1138-1142.

Hirose, S., Iwata, H., Akiyoshi, H., Kobayashi, K., Ito, M., Wada, K., Kaneko, S., and Mitsundome, A. (1999). A novel mutation of *CHRNA4* responsible for autosomal dominant nocturnal frontal lobe epilepsy. Neurology *53*, 1749-1753.

Hrvoje, H., Jasminka, S., Lipa, C.-S., Vida, D., and Branimir, J. (2010). Associatedion of serotonin transporter promoter (5-HTTLPR) and intron 2 (VNTR-2) polymorphisms with treatment response in temporal lobe epilepsy. Epilepy Res. *91*, 35-38.

Jain, S., Padma, M. V., Kanga, U., Mehra, N. K., Puri, A., and Maheshwari, M. C. (1999). Family studies and human leukocyte antigen class II typing in Indian probands with seizures in association with single small enhancing computer tomography lesions. Epilepsia *40*, 232-238.

Jian, L., Nagarajan, L., de Klerk, N., Ravine, D., Christodoulou, J., and Leonard, H. (2007). Seizures in Rett syndrome: an overview from a one-year calendar study. Eur. J. Paediatr. Neurol. *11*, 310-317.

Johnson, V. P. (1975). Smith-Lemli-Opitz syndrome: review and report of two affected siblings. Euro. J. Pediatr. *119*, 221-234.

Kamien, B., Harraway, J., Lundie, B., Smallhorne, L., Gibbs, V., Heath, A., and Fillerton, J. M. (2014). Characterization of a 520 kb deletion on chromosome 15q26.1 including *ST8SIA2* in a patient with behavioral disturbance, autism spectrum disorder, and epilepsy. Am. J. Hum. Genet. *164A*, 782-788.

Kato, M., Saitoh, S., Kamei, A., Shiraishi, H., Ueda, Y., Akasaka, M., Tohyama, J., Akasaka, N., and Hayasaka, K. (2007). A longer polyalanine expansion mutation in the *ARX* gene causes early infantile epileptic encephalopathy with suppression-burst pattern (Ohtahara syndrome). Am. J. Hum. Genet. *81*, 361-366.

Kerjan, G., Koizumi, H., Han, E. B., Dubé, C. M., Djakovic, S. N., Patrick, G. N., Baram, T. Z., Heinemann, S. F., and Gleeson, J. G. (2009). Mice lacking doublecortin and doublecortin-like kinase 2 display altered hippocampal neuronal maturation and spontaneous seizures. Proc. Natl. Acad. Sci. USA *106*, 6766-6771.

Kleefstra, T., Brunner, H. G., Amiel, J., Oudakker, A. R., Nillesen, W. M., Magee, A., Geneviève, D., Cormier-Daire, V., van Esch, H., Fryns, J.-P., et al. (2006). Loss-of-function mutations in *Euchromatin Histone Methyltransferase 1* (*EHMT1*) cause the 9q34 subtelomeric deletion syndrome. Am. J. Hum. Genet. *79*, 370-377.

Knerr, I., Pearl, P. L., Bottiglieri, T., Snead, O. C., Jakobs, C., and Gibson, K. M. (2007). Therapeutic concepts in succinate semialdehyde dehydrogenase (SSADH; ALDH5a1) defieciency (γ-hydroxybutyric aciduria). Hypotheses evolved from 25 years of patient evaluation, studies in *Aldh5a1*$^{-/-}$ mice and characterization of γ-hydroxybutyric acid pharmacology. J. Inher. Metab. Dis. *30*, 279-294.

Kulkantrakorn, K., and Geller, T. J. (1998). Seizures in neurofibromatosis 1. Pediatr. Neurol. *19*, 347-350.

Lal, D., Reinthaler, E. M., Altmüller, J., Toliat, M. R., Thiele, H., Nürnberg, P., Lerche, H., Hahn, A., Møller, R. S., Muhle, H., et al. (2013). *RBFOX1* and *RBFOX3* mutations in Rolandic epilepsy. PLoS One *8*, e73323.

Lalani, S. R., and Belmont, J. W. (2009). CHARGE syndrome. In Encyclopedia of Molecular Mechanisms of Disease. F. Lang, ed. (Berlin: Springer), pp. 312-313.

Lee, H., Lin, M. C., Kornblum, H. I., Papazian, D. M., and Nelson, S. F. (2014). Exome sequencing identifies de novo gain of function missense mutation in KCND2 in identical twins with autism and seizures that slows potassium channel inactivation. Hum. Mol. Genet. *23*, 3481-3489.

Lemke, J. R., Hendrickx, R., Geider, K., Laube, B., Schwake, M., Harvey, R. J., James, V. M., MBiol, A. P., Neidhardt, J., et al. (2014). *GRIN2B* mutations in west syndrome and intellectual disability with focal epilepsy. Ann. Neurol. *75*, 147-154.

Leoyklang, P., Suphapeetiporn, K., Siriwan, P., Desudchit, T., Chaowanapanja, P., Gahl, W. A., and Shotelersuk, V.(2007). Heterozygous nonsense mutation *SATB2* associated with cleft palate, osteoporosis, and cognitive defects. Hum. Mutat. *28*, 732-738.

Lev, D., Weigl, Hasan, M., Gak, E., Davidovich, M., Vinkler, C., Leshinsky-Silver, E., Lerman-Sagie, T., and Watemberg, N. (2007). A novel missense mutation in the *NDP*

gene in a child with Norrie disease and severe neurological involvement including infantile spasms. Am. J. Med. Genet. A *143A*, 921-924.

Liu, C.-S., and Tsai, C.-S. (2002). Enhanced lipid peroxidation in epileptics with null genotype of glutathione *S*-transferase M1 and intractable seizure. Japan. J. Pharmacol. *90*, 291-294.

Lodato, S., Rouaux, C., Quast, K. B., Jantrachotechatchawan, C., Studer, M., Hensch, T. K., and Arlotta, P. (2011). Excitatory projection neuron subtypes control the distribution of local inhibitory interneurons in the cerebral cortex. Neuron *69*, 763-779.

Lower, K. M., Turner, G., Kerr, B. A., Mathews, K. D., Shaw, M. A., Gedeon, Á. K., Schelley, S., Hoyme, H. E., White, S. M., Delatycki, M. B., et al. (2002). Mutations in *PHF6* are associated with Börjeson-Forssman-Lehmann syndrome. Nat. Genet. *32*, 661-665.

Marie, S., Cuppens, H., Heuterspreute, M., Jaspers, M., Tola, E. Z., Gu, X. X., Leguis, E., Vincent M.-F., Jaeken, J., Cassiman, J.-J., et al. (1999). Mutation analysis in adenylosuccinate lyase deficiency: eight novel mutations in the re-evaluated full ADSL coding sequence. Hum. Mutat. *13*, 197-202.

Marksteiner, J., Sperk, G., and Maas, D. (1989). Differential increases in brain levels of neuropeptide Y and vasoactive intestinal polypeptide after kainic acid-induced seizures in the rat. Naunyn Schmiedebergs Arch. Pharmacol. *339*, 173-177.

Martynyuk, A. E., Ucar, D. A., Yang, D. D., Norman, W. M., Carney, P. R., Dennis, D. M., and Laipis, P. J. (2007). Epilepsy in phenylketonuria: a complex dependence on serum phenylalanine levels. Epilepsia *48*, 1143-1150.

Maruta, K., Ohi, T., Ohdo, S., Takechi, T., Sakuragawa, N., and Matsukura, S. (1996). [A family with X-linked hydrocephalus resulting from mutations in the neural cell adhesion molecule L1]. Rinsho Shinkeigaku *36*, 462.-466.

Matsumoto, A., Kuwajima, M., Miyake, K., Kojima, K., Nakashima, N., Jimbo, E. F., Kubota, T., Momoi, M. Y., and Yamagata, T. (2013). An Xp22.12 microduplication including *RPS6KA3* identified in a family with variably affected intellectual and behavioral disabilities. J. Hum. Genet. *58*, 755-757.

Mefford, H. C., Muhle, H., Ostertag, P., von Spiczak, S., Buysse, K., Baker, C., Franke, A., Malafosse, A., Genton, P., Thomas, P., et al. (2010). Genome-wide copy number variation in epilepsy: novel susceptibility loci in idiopathic generalized and focal epilepsies. PLoS Genet. *6*, e1000962.

Meikle, L., Talos, D. M., Onda, H., Pollizzi, K., Rotenberg, A., Sahin, M., Jensen, F. E., and Kwiatkowski, D. J. (2007). A mouse model of tuberous sclerosis: neuronal loss of

Tsc1 causes dysplastic and ectopic neurons, reduced myelination, seizure activity, and limited survival. J. Neurosci. *27*, 5546-5558.

Mercimek-Mahmutoglu, S., Stoeckler-Ipsiroglu, S., Adami, A., Appleton, R., Araújo, H. C., Duran, M., Ensenauer, R., Fernandez-Alvarez, E., Garcia, P., Grolik, C., et al. (2006). GAMT deficiency. Neurology *67*, 480-484.

Minassian, B., Delorey, T. M., Olsen, R. W., Philppart, M., Bronstein, Y., Zhang, Q., Guerrini, R., van Ness, Paul, Livet, M. O., et al. (1998). Angelman syndrome: correlations between epilepsy phenotypes and genotypes. Ann. Neurol. *43*, 485-493.

Mochida, G. H. (2008). Genetic malformations of the cerebral cortex and seizure susceptibility during infancy and childhood. In Biology of Seizure Susceptibility in Developing Brain. T. Takahashi and Y. Fukuyama, eds. (Esher: John Libbey Eurotext Ltd.), pp. 175-184.

Mulle, C., Sailer, A., Pérez-Otaño, I., Dickinson-Anson, H., Castillo, P. E., Bureau, H., Maron, C., Gage, F. H., Mann, et al. (1998). Altered synaptic physiology and reduced susceptibility to kainite-induced seizures in GluR6-deficient mice. Nature *392*, 601-605.

Musemeci, S. A., Calabrese, G., Bonaccorso, C. M., D'Antoni, S., Brouwer, J. R., Bakker, C. E., Elia, M., Ferri, R., Nelson, D. L., Oostra, B. A., et al. (2007). Audiogenic seizure susceptibility is reduced in fragile X knockout mice after introduction of *FMR1* transgenes. Exper. Neurol. *203*, 233-240.

Nateri, A. S., Raivich, G., Gebhardt, C., Da Costa, C., Naumann, H., Vreugdenhil, M., Makwana, M., Brandner, S., Adams, R. H., Jefferys, J. G., et al. (2007). ERK activation causes epilepsy by stimulating NMDA receptor activity. EMBO J. *26*, 4891-4901.

Nowakowska, B. A., Obersztyn, E., Szymañska, K., Bekiesiñka-Figatowska, M., Xia, Z., Ricks, C. B., Bocian, E., Stockton, D. W., Szczałuba, K., Nawara, M., et al. (2010). Severe mental retardation, seizures, and hypotonia due to deletions of MEF2C. Am. J. Med. Genet. B Neuropsychiatr. Genet. *153B*, 1042-1051.

Online Mendelian Inheritance in Man (OMIM). (2014). Mucopolysaccharidosis, type IIIA; MPS3A. Accessed on 9/9/2014 from http://www.omim.org/entry/252900

Pavlidis, E., Cantalup, G., Bianchi, S., Piccolo, B., and Pisani, F. (2014). Epileptic features in Cornelia de Lange syndrome: case report and literature review. Brain Dev. Doi:10.1016/j.braindev.2013.12.008.

Pelc, K., Boyd, S. G., Cheron, G., and Dan, B. (2008). Epilepsy in Angelman syndrome. Seizure. *17*, 211-217.

Phillips, H. A., Favre, I., Kirkpatrick, M., Zuberia, S. M., Goudie, D., Heron, S. E., Scheffer, I. E., Sutherland, G. R., Berkovic, S. F., Bertrand, D., et al. (2001). *CHRNB2* is

the second acetylcholine receptor subunit associated with autosomal dominant nocturnal frontal lobe epilepsy. Am. J. Hum. Genet. *68*, 225-231.

Plecko, B., Paul, K., Paschke, E., Stoeckler-Ipsiroglu, S., Struys, E., Jakobs, C., Hartmann, H., Luecke, T., di Capua, M., Korenke, C., et al. (2007). Biochemical and molecular characterization of 18 patients with pyridoxine-dependent epilepsy and mutations of the antiquitin (*ALDH7A1*) gene. Hum. Mutat. *28*, 19-26.

Poeta, L., Fusco, F., Drongitis, D., Shoubridge, C., Manganelli, G., Filosa, S., Paciolla, M., Courtney, M., Collombat, P., Lioi, M. B., et al. (2013). A regulatory path associated with X-linked intellectual disability and epilepsy links KDM5C to the polyalanine expansions in ARX. Am. J. Hum. Genet. *92*, 114-125.

Ramirez, I. B.-R., Pietka, G., Jones, D. R., Divecha, N., Alia, A., Baraban, S. C., Hurlstone, A. F., and Lowe, M. (2012). Hum. Mol. Genet. *21*, 1744-1759.

Rijkers, K., Mescheriakova, J., Majoie, M., Lemmens, E., van Wijk, X., Philippens, M., Van Kranen-Mastenbroek, V., Schijns, O., Vles, J., and Hoogland, G. (2010). Polymorphisms in CACNA1E and Camk2d are associated with seizure susceptibility of Sprague-Dawley rats. Epilep. Res. *91*, 28-34.

Risbud, R. M., Lee, C., and Porter, B. E. (2011). Neurotrophin-3 mRNA a putative target of miR21 following status epilepticus. Brain Res. *1424*, 53-59.

Roberts, D. S., Raol, Y. H., Bandyopadhyay, S., Lund, I. V., Budreck, E. C., Passini, M. J., Wolfe, J. H., Brooks-Kayal, A. R., and Russek, S. J. (2005). Egr3 stimulation of *GABRA4* promoter activity as a mechanism for seizure-induced up-regulation of $GABA_A$ receptor α4 subunit expression. Proc. Natl. Acad. Sci. USA *102*, 11894-11899.

Roohi, J., Montagna, C., Tegay, D. H., Palmer, L. E., DeVincent, C., Pomeroy, J. C., Christian, S. L., Nowak, N., and Hatchwell, E. (2009). Disruption of contactin 4 in three subjects with autism spectrum disorder. J. Med. Genet. *46*, 176-182.

Rubenstein, M., Cepeda, C., Hurst, R. S., Flores-Hernandez, J., Ariano, M. A., Falzone, T. L., Kozell, L. B., Meshul, C. K., Bunzow, J. R., Low, M. J., et al. (2001). Dopamine D_4 receptor-deficient mice display cortical hyperexcitability. J. Neurosci. *21*, 3756-3763.

Saitsu, H., Kato, M., Osaka, H., Moriyama, N., Horita, H., Nishiyama, K., Yoneda, Y., Kondo, Y., Tsurusaki, Y., Doi, H., et al. (2012). *CASK* aberrations in male patients with Ohtahara syndrome and cerebellar hypoplasia. Epilepsia *53*, 1441-1449.

Sala, M., Braida, D., Lentini, D., Busnelli, M., Bulgheroni, E., Capurro, V., Finardi, A., Donzelli, A., Pattini, L., Rubino, L., et al. (2011). Biol. Psychiatry *69*, 875-882.

Salbert, B. A., Pellock, J. M., and Wolf, B. (1993). Characterization of seizures associated with biotinidase deficiency. Neurology *43*, 1351-1355.

Santos-Rebouças, C. B., Belet, S., de Almeida, L. G., Ribeiro, M. G., Medina-Acosta, E., Bahia, P. R. V., da Silva, A. F. A., dos Santos, F. L., de Lacerda, G. C. B., Pimentel, M. M., G., et al. (2014). A novel in-frame deletion affecting the BAR domain of *OPHN1* in a family with intellectual disability and hippocampal alterations. Euro. J. Hum. Genet. *22*, 644-651.

Scharfman, H. E., Goodman, J. H., Sollas, A. L., and Croll, S. D. (2002). Spontaneous limbic seizures after intrahippocampal infusion of brain-derived neurotrophic factor. Exp. Neurol. *174*, 201-214.

Seltzer, L. E., and Paciorkowski, A. R. (2014). Genetic disorders associated with postnatal microcephaly. Am. J. Med. Genet. *166*, 140-155.

Şenocak, E. U., Oğuz, K. K., Haliloğlu, G., Topçu, M., and Cila, A. (2010). Structural abnormalities of the brain other than molar tooth sign in Joubert syndrome-related disorders. Diagn. Interv. Radiol. *16*, 3-6.

Sohal, A. P. S., Montgomery, T., Mitra, D., and Ramesh, V. (2012). *TUBA1A* mutation-associated lissencephaly: case report and review of the literature. Pediatr. Neurol. *46*, 127-131.

Stevenson, R. E., Arena, J. F., Ouzts, E., Gibson, A., Shokeir, M. H. K., Vnencak-Jones, C., Lubs, H. A., May, M., and Schwartz, C. E. (1998). Renpenning syndrome maps to Xp11. Am. J. Hum. Genet. *62*, 1092-1101.

Stöckler, S., Braissant, O., and Schulze, A. (2014). Creatine disorders. In Physician's Guide to the Diagnosis, Treatment, and Follow-up of Inherited Metabolic Diseases.

Svenstrup, K., Møller, R. S., Christensen, J., Budtz-Jørgensen, E., Gilling, M., and Nielsen, J. E. (2011). Eur. J. Neurol. *18*, 1197-1199.

Szepetowski, P., and Monaco, A. P. (1998). Recent progress in the genetics of human epilepsies. Neurogenetics *1*, 153-163.

Takano, K., Nakagawam E., Inoue, K., Kamada, F., Kure, S., and Goto, Y. (2008). A loss-of-function mutation in the *FTSJ1* gene causes nonsyndromic X-linked mental retardation in a Japanese family. Am. J. Med. Genet Part B Neuropsych. Genet. *147B*, 479-484.

Talkowski, M. E., Mullegama, S. V., Rosenfeld, J. A., van Bon, B. W., Shen, Y., Repnikova, E. A., Gastier-Foster, J., Thrush, D. L., Kathiresan, S., Ruderfer, D. M., et al. (2011). Assessment of 2q23.1 microdeletion syndrome implicates MBD5 as a single causal locus of intellectual disability, epilepsy, and autism spectrum disorder. Am. J. Hum. Genet. *89*, 551-563.

Tarpey, P. S., Stevens, C., Teague, J., Edkins, S., O'Meara, S., Avis, T., Barthorpe, S., Buck, G., Butler, A., Cole, J., et al. (2006). Mutations in the gene encoding the sigma 2 subunit of the adaptor protein 1 complex, *AP1S2*, cause X-linked mental retardation. Am. J. Hum. Genet. *79*, 1119-1124.

Tatton-Brown, K., and Nazneen, R. (2004). Clinical features of NSD1-positive Sotos syndrome. Clin. Dysmorph. *13*, 199-204.

Teber, S., Sezer, T., Kafalı, M., Manzini, C., Yüksel, B. K., Tekin, M., Fitöz, S., Walsh, C. A., and Deda, G. (2008). Severe muscle-eye-brain disease is associated with a homozygous mutation in the *POMGnT1* gene. Europ. J. Paediatr. Neurol. *12*, 133-136.

Teskey, G. C., Radford, K. S., Seif, I., and Dyck, R. H. (2004). MAO(A) knockout mice are more susceptible to seizures but show reduced epileptogenesis. Epilepsy Res. *59*, 25-34.

Traylor, R. N., Dobyns. Rosenfeld, J. A., Wheeler, P., Spence, J. E., Bandholz, A. M., Bawle, E. V., Carmany, E. P., Powell, C. M., et al. (2012). Investigation of *TBR1* hemizygosity: four individuals with 2q24 microdeletions. Mol. Sydromol. *3*, 102-112.

Tripathi, P. P., Sgadò, P., Scali, M., Viaggi, C., Casarosa, S., Simon, H. H., Vaglini, F., Corsini, G. U., and Bozzi, Y. (2008). Increased susceptibility to kainic acid-induced seizures in *Engrailed-2* knockout mice. Neuroscience *159*, 842-849.

Turner, S. J., Hildebrand, M. S., Block, S., Damiano, J., Fahey, M., Reilly, S., Bahlo, M., Scheffer, I. E., and Morgan, A. T. (2013). Small intragenic deletion in FOXP2 associated with childhood apraxia of speech and dysarthria. Am. J. Med. Genet. A *161A*, 2321-2326.

Van Buggenhout, G., and Fryns, J. P. (2006). Lujan-Fryns syndrome (mental retardation, X-linked, marfanoid habitus). Orphanet J. Rare Dis. *1*, 26.

Vilarinho, L., Tafulo, S., Sibilio, M., Kok, F., Fontana, F., Diogo, L., Venâncio, M., Ferreira, M., Nogueira, C., Valongo, C., et al. (2010). Identification of novel L2HGDH gene mutations and update of the pathological spectrum. J. Hum. Genet. *55*, 55-58.

Yang, J., Houk, B., Hauser, K. F., Luo, Y., Smith, G., Schauwecker, E., and Barnes, G. N. (2005). Genetic background regulates semaphorin gene expression and epileptogenesis in mouse brain after kainic acid status epilepticus. Neuroscience *131*, 853-869.

Ylisaukko-oja, T., Rehnström, K., Vanhala, R., Tengström, Lähdetie, J., Järvelä, I. (2003). Identification of two AGTR2 mutations in male patients with non-syndromic mental retardation. Hum. Genet. *114*, 211-213.

Yoneda, Y., Saitsu, H., Touyama, M., Makita, Y., Miyamoto, A., Hamada, K., Kurotaki, N., Tomita, H., Nishiyama, K., Tsurusaki, Y., et al. (2012). Missense mutations in the

DNA-binding/dimerization domain of NFIX cause Sotos-like features. J. Hum. Genet. *57*, 207-211.

Zhao, K., Wen, R., Wang, X., Pei, L., Shang, Y., Bazan, N., Zhu, L.-Q., Tian, Q., and Lu, Y. (2013). EPAC inhibition of SUR1 receptor increases glutamate release and seizure vulnerability. J. Neurosci. *33*, 8861-8865.

Supplementary Table 3. General gene product function for each high-risk autism gene.

Aut Gene	Function
ACSL4	fatty acid metabolism; lipid metabolism
ADNP	transcription factor; transcription regulation
ADSL	purine biosynthesis
AFF2	mRNA processing; mRNA splicing
AGTR2	cell membrane; membrane; angiotensin II receptor
AHI1	cilium biogenesis/degradation
ALDH5A1	mitochondrion; GABA degradation
ALDH7A1	mitochondrion; detoxification
AP1S2	protein transport; transport
ARHGEF6	GTPase; guanine nucleotide exchange factor
ARID1B	transcription regulation; chromatin remodeling
ARX	transcription factor; transcription regulation
ASMT	melatonin biosynthesis
ATP13A4	membrane; cation-transporting ATPase activity
ATRX	DNA damage; DNA repair; transcription regulation; chromatin remodeling; ATPase
AUTS2	unknown
AVPR1A	vasopressin receptor
BCL2	apoptosis; mitochondrion
BDNF	growth factor
BRAF	kinase; transferase; intracellular signal transduction
BTD	hydrolase; biotin metabolism
C4B	immune regulation; complement pathway
CACNA1C	calcium transport; ion transport
CACNA1E	calcium transport; ion transport
CACNA1F	calcium transport; ion transport
CACNA1H	calcium transport; ion transport
CADM1	cell adhesion
CASK	scaffolding protein; ion channel trafficking; cytoskeleton; cell adhesion; calcium-dependent
CDH10	cell adhesion; calcium-dependent
CDH9	cell adhesion; calcium-dependent
CDKL5	kinase; methylation regulation
CEP290	cilium biogenesis/degradation; protein transport
CHD7	rRNA processing; transcription regulation; chromatin remodeling
CHD8	transcription regulation; chromatin remodeling
CHRNA4	acetylcholine receptor; ion transport
CHRNB2	acetylcholine receptor; ion transport; sodium and potassium transport
CNTN4	cell adhesion
CNTNAP2	cell adhesion

COMT	catecholamine metabolism; neurotransmitter degradation
CREBBP	histone acetylation; transcription regulation
CYFIP1	translation regulation; cytoskeleton
DBH	catecholamine biosynthesis
DCX	cytoskeleton
DHCR7	lipid biosynthesis/metabolism
DISC1	intracellular signaling; canonical Wnt pathway
DMD	cytoskeleton; ligand for dystroglycan
DMPK	kinase; transferase
DNAH5	cytoskeleton; maintenance of cilial integrity; ATPase
DRD3	dopamine receptor; G-protein coupled receptor
DRD4	dopamine receptor; G-protein coupled receptor
DYRK1A	kinase; transferase; nuclear signaling
EHMT1	histone methyltransferase; chromatin remodeling
EIF4E	translation regulation
EN2	transcription factor; transcription regulation
FBXO33	ubiquitination regulation; proteasomal degradation
FEZF1	transcription factor; transcription regulation
FEZF2	transcription factor; transcription regulation
FGD1	GTPase; cytoskeletal regulation
FGFR2	growth factor receptor
FHIT	FGF receptor; receptor kinase/transferase
FMR1	translation regulation; mRNA transport
FOXG1	transcription factor; transcription regulation
FOXP1	transcription factor; transcription regulation
FOXP2	transcription factor; transcription regulation
FTSJ1	tRNA processing; translation regulation; methyltransferase
GABRA4	GABA receptor subunit; ion transport; chloride regulation
GABRA5	GABA receptor subunit; ion transport; chloride regulation
GABRB1	GABA receptor subunit; ion transport; chloride regulation
GABRB3	GABA receptor subunit; ion transport; chloride regulation
GABRG3	GABA receptor subunit; ion transport; chloride regulation
GAD1	neurotransmitter biosynthesis
GAMT	creatine metabolism; methyltransferase
GATM	creatine metabolism; methyltransferase
GRIA3	glutamate receptor; ion transport; calcium regulation
GRIK2	glutamate receptor; ion transport
GRIN2B	glutamate receptor; ion transport; calcium regulation
GRIN3B	glutamate receptor; ion transport; calcium regulation
GRIP1	glutamate receptor interacting protein; scaffold protein; intracellular signal transduction
GRPR	gastrin releasing peptide receptor; g-protein coupled receptor

GSE1	unknown
GSTM1	transferase; glutathione conjugation; detoxification
GUCY2D	cGMP biosynthesis
HLA-DRB1	immune regulation
HOXA1	transcription factor; transcription regulation
HRAS	GTPase; intracellular signal transduction
IGF2	growth factor
IL1RAPL1	interleukin receptor; calcium regulation
IMMP2L	hydrolase; protease; mitochondrion
IQSEC2	guanine nucleotide exchange factor; GTPase regulation
ITGB3	cell adhesion; multi-ligand receptor; calcium-dependent
KCND2	voltage-gated potassium channel; ion transport; calcium activated
KCNMA1	voltage-gated potassium channel; ion transport; calcium activated
KDM5C	histone demethylase; chromatin remodeling; transcription regulation
KIAA0100	unknown
KIAA2022	cytoskeletal regulation; cell adhesion regulation
KRAS	GTPase
L1CAM	cell-cell adhesion/cytoskeleton
L2HGDH	L-2-hydroxyglutarate dehydrogenase; mitochondrion
LAMP2	lysosome regulation; extracellular signaling transduction; intracellular signaling transduction; detox
LRFN5	cell adhesion
LRP1	endocytic receptor; endocytosis & phagocytosis regulation; lipid metabolism
MACROD2	glutamate regulation; ADP ribosolation
MAOA	catecholamine metabolism; neurotransmitter degradation; mitochondrion
MAP2K1	kinase; transferase; intracellular signal transduction
MAPK1	kinase; transferase; intracellular signal transduction
MAPK3	kinase; transferase; intracellular signal transduction
MARK1	kinase; cytoskeleton; cell polarity
MBD5	chromatin remodeling
MECP2	methylation; chromatin remodeling; transcription regulation
MED12	transcription regulation
MEF2C	transcription regulation
MEGF11	cell adhesion
MET	hepatocyte growth factor receptor; intracellular signal transduction
MID1	ubiquitination regulation; cytoskeleton
MKKS	chaperone; cilium biogenesis; vesicular transport
MSNP1AS	regulation of moesin; cytoskeletal regulation
NDP	ligand; Wnt activation
NF1	GTPase; intracellular signal transduction
NFIX	transcription factor; transcription regulation
NHS	cytoskeleton

NIPA1	magnesium transport; ion transport
NIPBL	enhancer regulation; chromatin remodeling; chromatid cohesion
NLGN3	cell adhesion
NLGN4X	cell adhesion
NPHP1	cytoskeleton; cell polarity; cilium biogenesis/degradation
NRXN1	cell adhesion; calcium regulation
NRXN2	cell adhesion; calcium regulation
NSD1	histone methyltransferase; chromatin remodeling; transcription regulation
NTF3	growth factor
NTF4	growth factor
OCRL	cilium biogenesis/degradation; hydrolase; cytoskeleton; lysosome regulation
OPHN1	GTPase; cytoskeleton; intracellular signal trandsduction
OTC	transferase; mitochondrion; amino acid biosynthesis; arginine biosynthesis; urea cycle
OTX1	transcription factor; transcription regulation
OXTR	oxytocin receptor; g-protein coupled receptor; calcium-dependent
PAFAH1B1	GTPase regulation; cytoskeleton
PAH	phenylalanine catabolism; monooxygenase; oxidoreductase
PCDH19	cell adhesion; calcium-dependent
PHF6	transcription regulation
PHF8	histone regulation; histone lysine demethylase
PITX1	transcription factor; transcription regulation
POGZ	cytoskeleton; cell cycle progression; chromatid adhesion; kinetochore assembly
POMGNT1	O-mannosyl glycosylation; glycosyltransferase
POMT1	O-mannosyltransferase; glycosyltransferase
PQBP1	transcription factor; transcription regulation
PRKCB	kinase; intracellular signal transduction; calcium-dependent
PRKX	kinase; intracellular signal transduction
PRSS12	hydrolase; protease; cytoskeleton
PTCHD1	Shh receptor; intracellular signal transduction
PTEN	phosphatase; hydrolase; intracellular signal transduction; tumor suppressor
PTPN11	phosphatase; hydrolase; intracellular signal transduction
RAB39B	GTPase; vesicular regulation
RAI1	transcription regulation; chromatin remodeling
RAPGEF4	guanine nucleotide exchange factor; GTPase regulation; exocytosis regulation
RBFOX1	mRNA processing; alternative splicing regulation
RELN	cytoskeleton; cell adhesion; calcium-sensitive
RIMS3	cell adhesion; exocytosis regulation
RNF135	ubiquitination regulation; innate immunity
RPE65	hydrolase; isomeras
RPGRIP1L	cytoskeleton; cell adhesion; cilium regulation
RPL10	translation regulation

RPS6KA3	kinase; transferase; intracellular signal transduction
SATB2	transcription regulation; chromatin remodeling
SBF1	pseudophosphatase; guanine nucleotide exchange factor; GTPase
SCN1A	sodium transport; ion transport
SCN2A	sodium transport; ion transport
SEMA5A	cytoskeleton
SEZ6L2	endoplasmic reticulum regulation
SGSH	hydrolase; heparan sulfate degradation; lysosomal regulation
SHANK2	cell adhesion
SHANK3	cell adhesion
SLC22A9	transport
SLC25A12	glutamate-aspartate exchange; mitochondrion; transport; calcium-dependent
SLC6A4	serotonin transport; sodium- and chloride-dependent
SLC6A8	creatine regulation; creatine transport; sodium- & chloride-dependent
SLC9A6	sodium-hydrogen exchange; endosome regulation
SLCO1C1	anion transport; thyroid hormone transport; estradiol transport
SMC1A	cytoskeleton; chromatide cohesion; cell cycle progression; DNA repair
ST8SIA2	sialic acid transfer to oligosaccharides & glycoproteins; polysialic acid production; cell adhesion
SUV420H1	histone methyltransferase; chromatin remodeling; transcription regulation
SYN1	vesicular regulation;cytoskeleton; neurotransmitter release
TBR1	transcription factor; transcription regulation
TBX1	transcription factor; transcription regulation
TRIO	guanine nucleotide exchange factor; GTPase regulation; kinase; transferase; cytoskeleton regulation
TSC1	tumor suppressor; intracellular signal transduction
TSC2	tumor suppressor; intracellular signal transduction; cytoskeletal-mediated transport; GTPase
TTN	cytoskeleton; chromosome condensation; chromosome segregation
TUBA1A	cytoskeleton; chromosome segregation
TUBGCP5	cytoskeleton; centromere formation
UBE3A	ubiquitination regulation
UPF3B	mRNA export; mRNA surveillance; translation regulation
UPP2	glycosyltransferase; production of carbon & energy sources; nucleotide synthesis; detoxification
VIP	ligand; hormone
VPS13B	vesicular regulation
YWHAE	adaptor protein; intracellular signal transduction
ZNF674	transcription factor; transcription regulation
ZNF81	transcription factor; transcription regulation
